# Arginase 1 drives mitochondrial cristae remodeling and PANoptosis in ischemia/hypoxia-induced vascular dysfunction

**DOI:** 10.1038/s41392-025-02255-2

**Published:** 2025-05-28

**Authors:** Han She, Jie Zheng, Guozhi Zhao, Yunxia Du, Lei Tan, Zhe-Sheng Chen, Yinyu Wu, Yong Li, Yiyan Liu, Yue Sun, Yi Hu, Deyu Zuo, Qingxiang Mao, Liangming Liu, Tao Li

**Affiliations:** 1 Department of Anesthesiology, Daping Hospital, Army Medical University, Chongqing, 400042 China; 2 Shock and Transfusion Department, Daping Hospital, Army Medical University, Chongqing, 400042 China; 3 Department of Respiratory Disease, Daping Hospital, Army Medical University, Chongqing, 400042 China; 4 Department of Urology Surgery, The First Affiliated Hospital of Chongqing Medical University, Chongqing, 400016 China; 5 Department of Pharmaceutical Sciences, College of Pharmacy and Health Sciences, St. John’s University, Queens, NY 11439 USA; 6 Department of Rehabilitation Medicine, The First Affiliated Hospital of Chongqing University of Chinese Medicine, Chongqing Traditional Chinese Medicine Hospital, Chongqing, 400021 China; 7 Department of Research and Development, Chongqing Precision Medical Industry Technology Research Institute, Chongqing, 400000 China

**Keywords:** Molecular biology, Cardiology

## Abstract

Ischemic/hypoxic injury significantly damages vascular function, detrimentally impacting patient outcomes. Changes in mitochondrial structure and function are closely associated with ischemia/hypoxia-induced vascular dysfunction. The mechanism of this process remains elusive. Using rat models of ischemia and hypoxic vascular smooth muscle cells (VSMCs), we combined transmission electron microscopy, super-resolution microscopy, and metabolic analysis to analyze the structure and function change of mitochondrial cristae. Multi-omics approaches revealed arginase 1 (Arg1) upregulation in ischemic VSMCs, confirmed by in vivo and in vitro knockout models showing Arg1’s protective effects on mitochondrial cristae, mitochondrial and vascular function, and limited the release of mtDNA. Mechanistically, Arg1 interacting with Mic10 led to mitochondrial cristae remodeling, together with hypoxia-induced VDAC1 lactylation resulting in the opening of MPTP and release of mtDNA of VSMCs. The released mtDNA led to PANoptosis of VSMCs via activation of the cGAS-STING pathway. ChIP-qPCR results demonstrated that lactate-mediated Arg1 up-regulation was due to H3K18la upregulation. VSMCs targeted nano-material PLGA-PEI-siRNA@PM-α-SMA (NP-siArg1) significantly improved vascular dysfunction. This study uncovers a new mechanism of vascular dysfunction following ischemic/hypoxic injury: a damaging positive feedback loop mediated by lactate-regulated Arg1 expression between the nucleus and mitochondria, leading to mitochondria cristae disorder and mtDNA release, culminating in VSMCs PANoptosis. Targeting VSMCs Arg1 inhibition offers a potential therapeutic strategy to alleviate ischemia/hypoxia-induced vascular impairments.

## Introduction

Ischemic/hypoxic injury, such as severe traumatic shock and sepsis, might lead to vascular dysfunction, which is characterized by diminished or absent responsiveness to vasoactive agents, severely affecting the prognosis of patients.^1–3^ Emerging research indicates that ischemic/hypoxic injury-induced vascular dysfunction is mainly attributed to the irregularities of membrane ion channels and receptors of vascular smooth muscle cells (VSMCs), and the excessive generation of oxygen free radicals.^3–5^ Despite of the proposition of multiple targeted interventions, notable advancements have been limited. Thus, delving into the novel mechanisms of vascular dysfunction subsequent to ischemia/hypoxia and identifying efficacious therapeutic approaches is of paramount importance.

Mitochondria are critical organelles that orchestrate a variety of cellular functions.^6–8^ Evidence suggests a close link between mitochondria and vascular dysfunction. Our prior studies have demonstrated the involvement of mitochondrial dynamic imbalance in VSMCs under ischemic/hypoxic conditions, which in turn impairs vascular function.^9^ Furthermore, deregulated mitochondrial metabolism in endothelial cells has been implicated in the onset of ferroptosis.^10^ Emerging research shows the significance of mitochondrial cristae integrity for mitochondrial functionality.^11,12^ Mitochondrial cristae are invaginations of the inner membrane where electron transport chain complexes and ATP synthase reside, which play an essential role in oxidative phosphorylation and ATP generation.^13^ The Mitochondrial Contact Site and Cristae Organizing System (MICOS) is integral to the architecture and stability of cristae. Comprising principally two subcomplexes centered around Mic10 and Mic60, MICOS malfunction due to the absence of these core elements reveals marked cristae malformations, culminating in widespread junctional loss, cristae detachment from the inner membrane, and enlarged inner membrane stack formation.^14,15^ Notably, Mic10-mediated membrane curvature is required for cristae junction assembly.^16^ The interplay among alterations of mitochondrial function in VSMCs under ischemic/hypoxic injury, cristae morphological changes, and the contributory role of Mic10 remains to be elucidated.

Arginase 1 (Arg1), a binuclear manganese metalloenzyme, is involved in various metabolic pathways in both normal and pathological states, including catalyzing the last step of the ornithine-urea cycle, converting L-arginine into L-ornithine and urea.^17^ Arg1 is primarily localized in the lysosomes, cytoplasm, and nucleus. Recent studies have also found that it can be located in the mitochondria, but its function in the mitochondria is unclear.^18^ Arg1 is predominantly expressed in the liver, myocardium, vascular smooth muscle, and endothelial cells.^19^ Arginase activity has been linked to vascular dysfunction in the pathogenesis of essential hypertension, and it also contributes to atherosclerosis (AS) progression by amplifying leukocyte response in inflammation.^20,21^ Reducing Arg1 markedly decreases reactive oxygen species (ROS) production and improves endothelial function.^22^ However, it is not clear whether Arg1 is involved in regulating mitochondrial function and whether the effect of Arg1 on vascular function is related to mitochondria.

Mitochondrial DNA (mtDNA) is a kind of self-replicating double-stranded DNA, that typically exists in circular or linear forms inside the mitochondrial matrix. Prior research has established that mtDNA release into the cytoplasm can initiate innate immune responses and inflammation, yet the mechanisms of this process remain elusive.^23^ Studies have demonstrated that mitochondrial permeability transition pore (mPTP) opening can promote mtDNA release to activate inflammatory pathways.^24^ Moreover, mitochondria experiencing oxidative stress can emit short mtDNA fragments via channels formed by the oligomerization of the Voltage-Dependent Anion Channel (VDAC) in their outer membrane.^25^ Recent research has clarified that structural modifications in the inner mitochondrial membrane, particularly the cristae, can trigger the translocation of Bax proteins to the mitochondria, resulting in the release of mtDNA.^26,27^ The cytosolic cyclic GMP-AMP Synthase (cGAS) acts as a dsDNA sensor, binding to extramitochondrial mtDNA and inducing a conformational shift that activates the STimulator of INterferon Genes (STING) signaling pathway via cGAMP.^28^ This cGAS-STING axis is pivotal in vascular function regulation, as seen in AS, where oxidative stress inflicts damage on VSMCs, releasing mtDNA that activates the cGAS-STING pathway and fosters the inflammatory transformation of VSMCs.^29^ However, the association of ischemic/hypoxic injury-induced vascular dysfunction, mtDNA release and cGAS-STING pathway activation remains to be clarified.

In recent years, a novel form of cell death known as PANoptosis has emerged as a significant focus in scientific research. PANoptosis represents a unique programmed cell death modality that integrates characteristic features of pyroptosis, apoptosis, and necroptosis.^30,31^ PANoptosis can be activated by diverse cellular stress signals and plays crucial roles in various pathological processes, including infection, inflammation, and cancer.^32–34^ The hallmark of PANoptosis lies in its sophisticated regulatory network, which involves the activation of inflammasomes, participation of caspase family proteins, and modulation of RIPK signaling pathways.^33,35,36^ However, the potential involvement of PANoptosis in ischemic/hypoxic injury-induced vascular dysfunction remains to be elucidated.

This study aims to elucidate the pivotal role of Arg1 in mediating mitochondrial cristae remodeling, mtDNA release, and subsequent activation of the cGAS-STING signaling pathway leading to PANoptosis during ischemic/hypoxic injury-induced vascular dysfunction. Through the identification of novel therapeutic targets for vascular impairment, we investigate a nanotechnology-based targeted strategy for the treatment of ischemic/hypoxic injury.

## Results

### Mitochondrial cristae damage is related to ischemia/hypoxia-induced vascular dysfunction

**Fig. 1:**
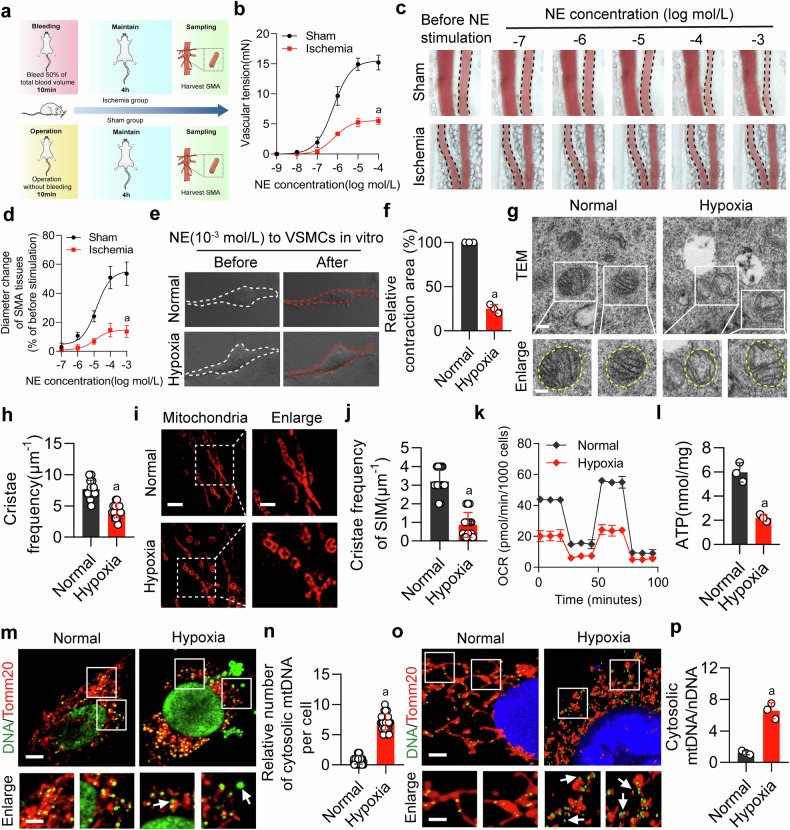
Ischemic/hypoxic injury results in vascular dysfunction characterized by impaired mitochondrial cristae architecture and mitochondrial dysfunction in VSMCs. **a** Schematic diagram of the rat ischemic injury model. **b** Quantification of vascular responsiveness in SMA tissues to NE stimulation (n = 6 rats in each group). **c** Changes in the SMA diameter were observed for varying NE concentrations (10^−7^ to 10^−3^ mol/L), with artery perimeters delineated by dotted lines, while the others without outlines were veins (n = 6 rats in each group). **d** The statistics of diameter change in SMA reacting to NE stimulation (n = 6 rats in each group). **e** The response of VSMCs to 10^−3^ mol/L NE was measured, with cellular boundaries pre- and post-stimulation marked in white and red dotted curves, respectively (n = 3 independent experiments). **f** Statistical results of VSMCs relative contraction area (n = 3 independent experiments). **g** The mitochondrial structure of VSMCs examined via transmission electron microscopy, scale bars correspond to 0.25 μm for low-magnification images and 0.15 μm for high-magnification views (n = 3 independent experiments). **h** The cristae frequency within mitochondria was assessed statistically by examining 15 randomly chosen mitochondria, calculating cristae frequency as the number of cristae per mitochondrial length unit. Data represent three independent experiments. **i** Representative images of mitochondria cristae of VSMCs stained with 200 nM Mitotracker Red for 15 min. The images were captured by Hessian-SIM super resolution microscope, scale bars correspond to 2.5 μm for low-magnification images and 1 μm for high-magnification views (n = 3 independent experiments). **j** The cristae frequency was statistically analyzed by examining 15 randomly chosen mitochondria, calculating cristae frequency as the number of cristae per mitochondrial length unit. Data represent three independent experiments. **k** Mitochondrial OCR in VSMCs was measured using the Seahorse XF Analyzer. The Agilent Seahorse XF Cell counting software was utilized to calculate cell numbers (n = 3 independent experiments). **l** Detection of ATP levels using the luciferase assay (n = 3 independent experiments). **m** mtDNA release was visualized using confocal microscopy, after immunolabeling VSMCs with anti-TOM20 (red) and anti-DNA (green) antibodies, scale bars correspond to 5 μm for low-magnification images and 2.5 μm for high-magnification views (n = 3 independent experiments). **n** The number of cytosolic mtDNA puncta per cell as quantitated (n = 30 cells/group). Data represent three independent experiments. **o** Observation of mtDNA release by confocal three-dimensional layer scanning, scale bars correspond to 5 μm for low-magnification images and 2.5 μm for high-magnification views (n = 3 independent experiments). **p** Ratio of mtDNA/nDNA in VSMCs (n = 3 independent experiments). a: p < 0.05, as compared with the Sham or Normal group

The effects of ischemia/hypoxia on vascular function were observed. Compared to the sham group, the contractile response to NE in the isolated SMA from ischemic rats was significantly diminished (Fig. 1a, b). NE stimulation at a concentration of 10^-3 ^mol/L led to a marked reduction in the SMA diameter in sham rats. However, this reaction was not observed in ischemic rats, regardless of the NE dosages (Fig. 1c, d). Furthermore, the contractility of VSMCs was examined. A significantly reduction of contractility was discerned in hypoxic VSMCs compared to the normal group after NE administration (Fig. 1e, f). However, compared to the Sham group, the levels of nitric oxide and neuronal nitric oxide synthase (nNOS) in SMA of ischemic rats did not show a significant difference (Supplementary Fig. S1a–c), indicating that nitric oxide and nNOS in SMA are not related to ischemia/hypoxia-induced vascular dysfunction. Notably, a conspicuous decline in the 24-hour survival rate and survival time was observed in ischemic rats compared to sham rats (Supplementary Fig. S1d, e).

We further investigated whether changes in mitochondria are involved in ischemia/hypoxia-induced vascular dysfunction. We found that hypoxic VSMCs exhibited disordered mitochondrial cristae structures, an increased number of vacuoles, and a decreased frequency of cristae (Fig. 1g, h). Observations via Hessian-SIM super-resolution microscopy revealed a trend similar to that of TEM results (Fig. 1i, j). This was conjoined with mitochondrial dysfunction, as discerned from significantly reduced oxygen consumption rate (OCR), ATP, heightened ROS production and a diminished mitochondrial membrane potential (Fig. 1k, l; Supplementary Fig. S1f–i). Moreover, the release of mtDNA in hypoxic VSMCs was significantly increased (Fig. 1m–p). However, using the NOS inhibitor (L-NAME) on VSMCs did not induce mitochondrial dysfunction and mtDNA release (Supplementary Fig. S1j–o). These findings confirm that mitochondrial cristae damage is related to ischemia/hypoxia-induced vascular dysfunction.

### Intervention of Arg1 improves vascular function and mitochondrial cristae structure

**Fig. 2:**
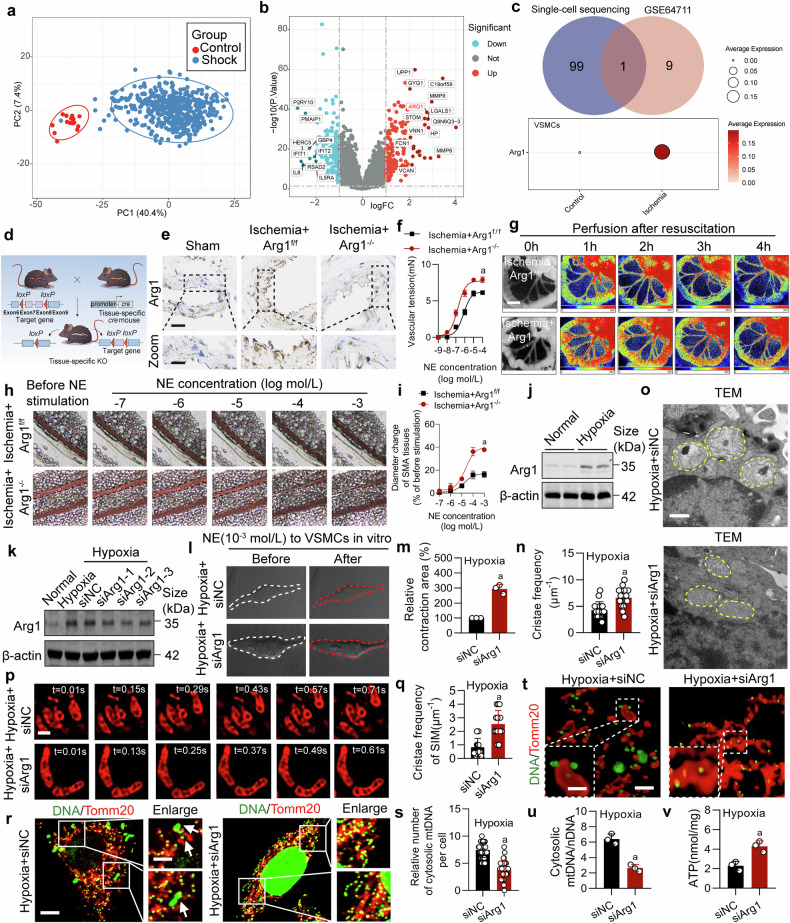
Intervention of Arg1 enhances vascular function and restore the structure of mitochondrial cristae following ischemic/hypoxic injury. **a** The principal component analysis (PCA) score plot for GSE64711 distinguishes between healthy controls and shock patients. **b** The differential gene expression volcano plot comparing shock to control reveals upregulated genes in red and downregulated genes in blue. **c** Single-cell sequencing analysis of Arg1 level in VSMCs. **d** Construction of the Arg1^-/-^ mouse. **e** Immuno-histological analysis revealing the Arg1 expression in SMA tissues of Arg1^-/-^ mice with ischemia, scale bars correspond to 30 μm for low-magnification images and 15 μm for high-magnification views (n = 6 mice in each group). **f** The vascular reactivity of SMA upon NE stimulation in Arg1^-/-^ mice was quantified (n = 6 mice in each group). **g** Laser speckle contrast imaging was utilized to monitor the intestinal blood flow in Arg1^-/-^ mice. Following the establishment of the ischemia model, the mice were administered a dose of lactated Ringer solution (equivalent to twice the volume of blood loss) through the right femoral vein within 30 minutes (bar=1 cm) (n = 6 mice in each group). **h** Variations in SMA diameter reacting to NE stimulation (concentrations ranging from 10^-7^ to 10^-3 ^mol/L) of ischemic Arg1^-/-^ mice was observed (n = 6 mice in each group). **i** The statistics of diameter change in SMA of ischemic Arg1^-/-^ mice reacting to NE stimulation (n = 6 mice in each group). **j** Western blot conducted to measure the expression of Arg1 in VSMCs post-hypoxia treatment (n = 3 independent experiments). **k** The expression of Arg1 in hypoxic VSMCs following transfection with three distinct siRNAs was detected (n = 3 independent experiments). **l** The impact of siArg1 on the responsiveness of VSMCs to 10^-3 ^mol/L NE was examined (n = 3 independent experiments). **m** Statistical results of VSMCs relative contraction area (n = 3 independent experiments). The impact of siArg1 on VSMC mitochondrial structure was evaluated using transmission electron microscopy (**n**, **o**) (bar=500 nm) and Hessian-SIM super resolution microscope (**p**, **q**) (bar = 1 μm) (n = 3 independent experiments). **r** The levels of mtDNA release due to siArg1 were measured, scale bars correspond to 5 μm for low-magnification images and 2.5 μm for high-magnification views (n = 3 independent experiments). **s** The number of cytosolic mtDNA puncta per cell as quantitated (n = 30 cells/group). Data represent three independent experiments. **t** Observation of mtDNA release by confocal three-dimensional layer scanning, scale bars correspond to 5 μm for low-magnification images and 2.5 μm for high-magnification views (n = 3 independent experiments). **u** Ratio of mtDNA/nDNA in VSMCs (n = 3 independent experiments). **v** The impact of siArg1 on ATP levels (n = 3 independent experiments). a: p < 0.05, as compared with the Ischemia + Arg1^f/f^ or Hypoxia + siNC group

To further delve into the mechanisms of vascular dysfunction and mitochondrial cristae disorder in ischemic/hypoxic injury, we analyzed the whole blood transcriptome dataset (GSE64711) of hemorrhagic shock patients available on the Gene Expression Omnibus (GEO) database (https://www.ncbi.nlm.nih.gov/geo/).^37^ The dataset includes 478 hemorrhagic shock patients and 17 healthy individuals as controls. Principal Component Analysis (PCA) results and volcano diagrams highlight a clear contrast between the transcriptome data of the Control and Shock group, showing a total of 185 upregulated and 154 downregulated differential genes (selection criteria: Shock vs. Control, |log2FoldChange | > 1, p < 0.05). We intersected the top 10 upregulated genes from GSE64711 with the differential genes (Top100 upregulated: Ischemia vs. Control, |log2FoldChange | > 0.25, p < 0.05) from single-cell sequencing in the ischemic mice’ VSMCs, and found Arg1 was the only overlapping upregulated gene (Fig. 2a–c and Supplementary Table S1). Prior studies showed that pro-inflammatory factors such as lipopolysaccharide and TNF-alpha can induce Arg1 expression.^38,39^ To elucidate the role of Arg1 in ischemia/hypoxia-induced vascular dysfunction, we generated Arg1 VSMCs conditional knockout (KO) mice (Arg1^-/-^) and Arg1 knockdown (KD) rats treated by adeno-associated virus (AAV) (SM22ap-EGFP-mir155(MCS)-SV40 PolyA) (Fig. 2d and Supplementary Fig. S2a). We evaluated SMA and found a significant increase of Arg1 expression in the ischemia group, while both Arg1^-/-^ and Arg1 KD showed a significant decrease of Arg1 by immunohistochemical analysis (Fig. 2e and Supplementary Fig. S2b). Comparing with the ischemia group, we found that Arg1 KO or KD effectively improved the contractile reactivity of SMA and increased intestinal blood flow (Fig. 2f–i and Supplementary Fig. S2c–e). Additionally, Arg1 KD also improved the survival rate of ischemic rats (Supplementary Fig. S2f, g).

In our in vitro experiments, we observed a significant upregulation of Arg1 in hypoxic VSMCs (Fig. 2j and Supplementary Fig. S3a). Then, we introduced Arg1 interference in vitro using siRNA (Fig. 2k and Supplementary Fig. S3b). The contractility of hypoxic VSMCs in the siArg1 group significantly increased (Fig. 2l, m). We also noted the effect of Arg1 KD on the cristae structure within VSMCs’ mitochondria and mtDNA release. Interestingly, Arg1 KD can effectively improve mitochondrial cristae in hypoxic VSMCs, as evidenced by increased cristae frequency (Fig. 2n–q). Additionally, Arg1 KD can also inhibit mtDNA release and improve mitochondrial function, including inhibiting ROS generation, upregulating OCR and ATP levels, and increasing the mitochondrial membrane potential (Fig. 2r–v and Supplementary Fig. S3c–g). The results indicate that the intervention of Arg1 enhances vascular function and the structure of mitochondrial cristae.

Since arginase has two isoforms,^40^ we compared the expression of Arg2 under normal and hypoxia conditions and found no significant differences. Additionally, interfering with Arg1 had no significant impact on Arg2 expression in VSMCs (Supplementary Fig. S4a, b). Furthermore, we examined the effects on the levels of L-arginine and urea in VSMCs under normal, hypoxia, and Arg1-interfered conditions. The results showed that L-arginine levels significantly decreased, and urea production increased after hypoxia (Supplementary Fig. S4c, d). Interfering with Arg1 increased L-arginine levels and inhibited urea production, but exogenously adding urea did not counteract the inhibitory effect of Arg1 interference on mtDNA release after hypoxia. Moreover, L-arginine^41^ supplementation did not improve the vascular reactivity of ischemic rats (Supplementary Fig. S4e, f), indicating that the regulatory effect of Arg1 on ischemia/hypoxia-induced vascular dysfunction is independent of its enzymatic activity.

### Interaction of Arg1 and Mic10 in VSMCs leads to conformation changes of Mic10

**Fig. 3:**
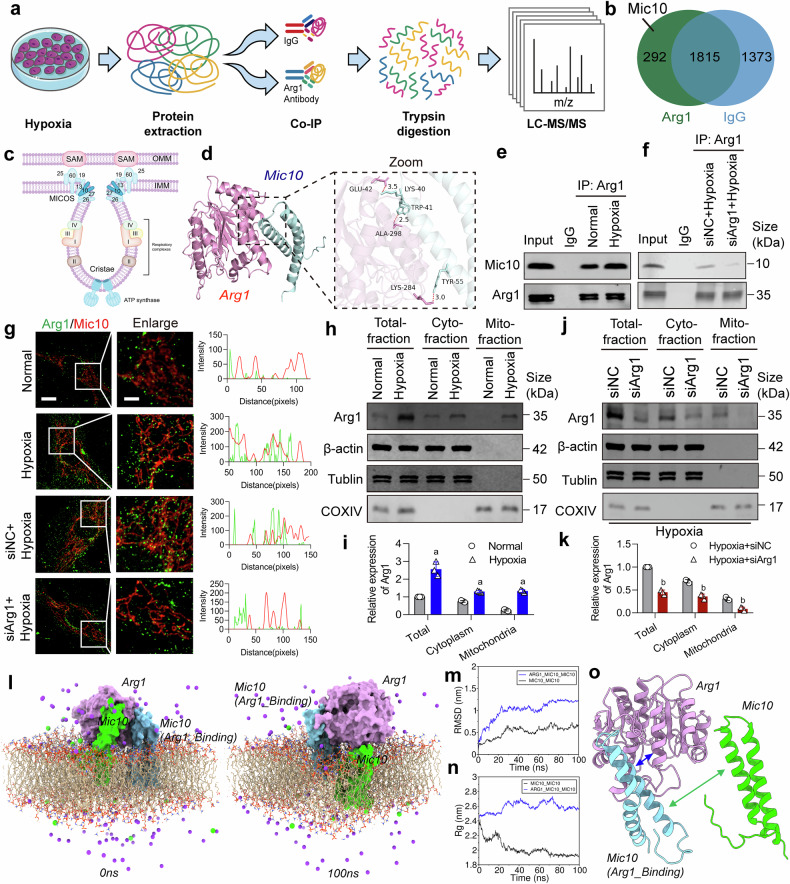
Exposure of VSMCs to hypoxia enhances the interaction between Arg1 and Mic10, eliciting conformational alterations in Mic10. **a** A schema illustrates the LC-MS/MS approach for detecting Arg1-interacting proteins. **b** A Venn diagram identified proteins that bind to Arg1. **c** A schematic representation of the mitochondrial cristae structure and the MICOS complex. OMM outer mitochondrial membrane, IMM inner mitochondrial membrane. **d** Molecular docking between Arg1 and Mic10, and the visualization of the binding sites are presented. **e** A co-immunoprecipitation (Co-IP) assay confirmed the interaction between Arg1 and Mic10 (n = 3 independent experiments). **f** Co-IP experiment assessed the impact of siArg1 on the interaction of Arg1 and Mic10 (n = 3 independent experiments). **g** Immunofluorescence microscopy revealed the colocalization of Arg1 and Mic10, shown in green and red, respectively, scale bars correspond to 8 μm for low-magnification images and 4 μm for high-magnification views (n = 3 independent experiments). **h**, **i** The expression and statistical analysis of Arg1 in total, cytoplasmic, and mitochondrial fractions of VSMCs following hypoxia. β-actin, Tubulin, and COX IV served as internal references for the total, cytoplasmic, and mitochondrial fractions, respectively (n = 3 independent experiments). **j**, **k** The expression and statistical analysis of Arg1 in total, cytoplasmic, and mitochondrial fractions of hypoxic VSMCs after being treated with siRNA (n = 3 independent experiments). **l** Arg1 binds to Mic10 and inhibits the polymerization between MIC10 monomers. **m** The Root Mean Square Deviation analysis. **n** The radius of gyration (Rg) analysis. **o** Analysis of hydrogen bond formation. a: p < 0.05, as compared with the Normal group; b: p < 0.05, as compared with the Hypoxia + siNC group

To further unravel the mechanism by which Arg1 regulates the mitochondrial cristae structure and mtDNA release of VSMCs after ischemia/hypoxia injury, we analyzed hypoxic cells and identified 292 proteins that interact with Arg1 using co-immunoprecipitation combined with proteomics (Fig. 3a, b). We specifically scrutinized proteins associated with the cristae structure in mitochondria and discovered that Mic10, a key subunit of the MICOS complex, interacts with Arg1 (Fig. 3c). The results of molecular docking revealed that Mic10 and the Glu42 (E42) site of Arg1 have the strongest binding affinity (Fig. 3d and Supplementary Fig. S5a, b). The results of Co-immunoprecipitation and fluorescence co-localization suggested that hypoxia enhanced the binding of Arg1 and Mic10 compared to the normal condition. However, Arg1 interference resulted in a diminished interaction between Arg1 and Mic10, reflected by a reduction in their binding and a consequent decrease in the co-localization of fluorescence (Fig. 3e–g). Furthermore, neither hypoxia nor Arg1 interference affects Mic10 expression levels (Supplementary Fig. S5c, d). Meanwhile, the detection of cytoplasmic and mitochondrial proteins revealed that mitochondrial Arg1 significantly increased after hypoxia, whereas siArg1 significantly reduced mitochondrial Arg1 levels (Fig. 3h–k). The above results suggested that hypoxia leads to an upregulation of mitochondrial Arg1 and an increased interaction with Mic10, causing disorganization of the cristae.

We examined the conformational changes of the Mic10 protein upon binding to the Arg1 (E42) site through molecular dynamics (MD) simulations. Using free energy landscape analysis, we selected the optimum conformation for Arg1 (E42) and Mic10 binding (Supplementary Fig. S5e). A 100-ns MD simulation revealed that the Root Mean Square Fluctuation (RMSF) of Mic10, reflecting atomic positional changes over time, exhibited significant alterations in structural stability upon Arg1 binding (Supplementary Fig. S5f). Additionally, the radius of gyration (Rg) analysis demonstrated a marked increase in the volume and shape of Mic10 after interacting with Arg1 (Supplementary Fig. S5g).

We analyzed the self-assembly of Mic10 monomers within the membrane. Results showed that Mic10 monomers spontaneously polymerized in the membrane (Supplementary Fig. S5h), whereas Arg1 binding to Mic10 disrupted this oligomerization (Fig. 3l). The Root Mean Square Deviation (RMSD) of the Arg1_Mic10_Mic10 complex was significantly higher than that of the Mic10_Mic10 group, indicating that Arg1 inhibits Mic10 monomer aggregation and reduces structural stability (Fig. 3m). Furthermore, the Rg value of the Mic10_Mic10 system sharply decreased, reflecting enhanced compactness due to the spontaneous assembly of Mic10 monomers. In contrast, the Rg of the Arg1_Mic10_Mic10 complex remained notably higher than that of the Mic10_Mic10 group, confirming Arg1-mediated suppression of Mic10 oligomerization (Fig. 3n). Hydrogen bond analysis revealed stable binding between Arg1 and Mic10, with five hydrogen bonds formed at their interface (blue arrows, Fig. 3o and Supplementary Fig. S5i). Notably, in the presence of Arg1, no hydrogen bonds were observed between the two Mic10 monomers (green arrows, Fig. 3o), further supporting the inhibitory role of Arg1 in Mic10 homo-oligomerization. The results indicate that Arg1, through its interaction with Mic10, suppresses Mic10 homo-oligomerization, thereby affecting the structure of mitochondrial cristae.

### Arg1 E42A mutation reverses mitochondrial cristae disorder in VSMCs

**Fig. 4:**
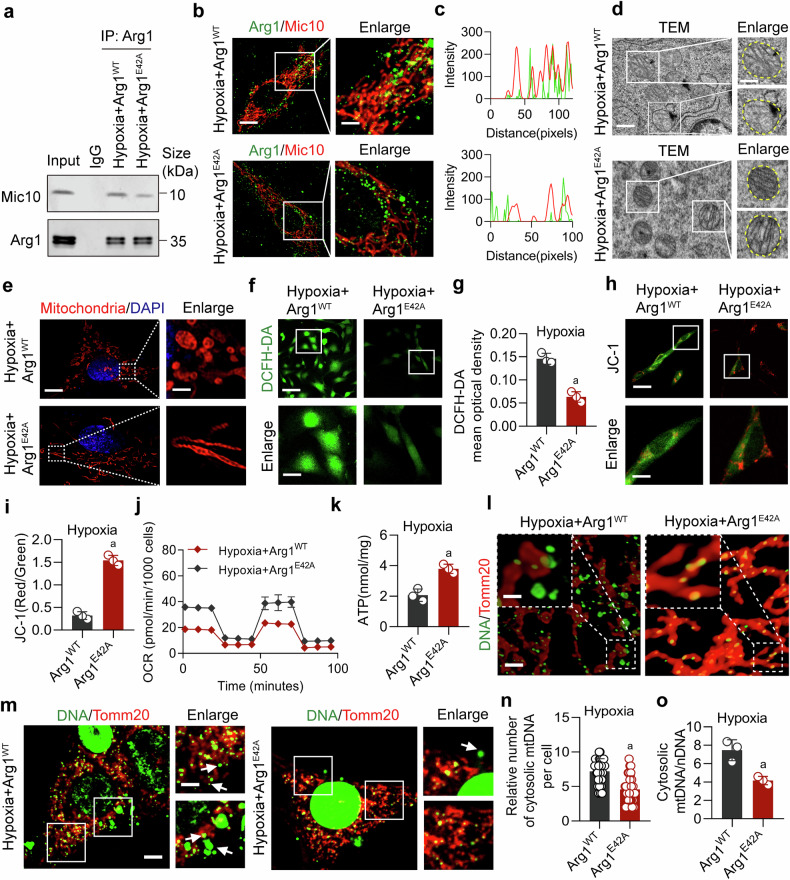
Arg1 E42A mutation reverse hypoxia-induced mitochondrial cristae disorganization in VSMCs. **a** The effect of the Arg1 E42A mutation on the interaction between Arg1 and Mic10 assessed using Co-IP analysis (n = 3 independent experiments). **b** The effect of Arg1 E42A mutation on the colocalization of Arg1 and Mic10 fluorescence, with Arg1 labeled in green and Mic10 in red, scale bars correspond to 8 μm for low-magnification images and 4 μm for high-magnification views (n = 3 independent experiments). **c** Fluorescence colocalization was analyzed by ImageJ. **d** TEM was used to examine the influence of the Arg1 E42E mutation on mitochondrial cristae morphology, scale bars correspond to 300 nm for low-magnification images and 150 nm for high-magnification views (n = 3 independent experiments). **e** Representative images of mitochondria cristae of VSMCs stained with 200 nM Mito-tracker Red for 15 min, scale bars correspond to 5 μm for low-magnification images and 2 μm for high-magnification views (n = 3 independent experiments). The Arg1 E42A mutation’s effects on **f**, **g** ROS, scale bars correspond to 30 μm for low-magnification images and 10 μm for high-magnification views and **h**, **i** mitochondrial membrane potential, scale bars correspond to 40 μm for low-magnification images and 20 μm for high-magnification views (n = 3 independent experiments). **j**, **k** The Arg1 E42A mutation’s effects on OCR and ATP of VSMCs (n = 3 independent experiments). **l** Observation of mtDNA release by confocal three-dimensional layer scanning, scale bars correspond to 5 μm for low-magnification images and 2.5 μm for high-magnification views (n = 3 independent experiments). **m** The effect of Arg1E42A on mtDNA release was measured, scale bars correspond to 5 μm for low-magnification images and 2.5 μm for high-magnification views (n = 3 independent experiments). **n** The number of cytosolic mtDNA puncta per cell as quantitated (n = 30 cells/group). Data represent three independent experiments. **o** Ratio of mtDNA/nDNA in VSMCs (n = 3 independent experiments). a: p < 0.05, as compared with the Hypoxia + Arg1^WT^ group

To further clarify the effect of Arg1 on the mitochondrial cristae of hypoxic VSMCs, we mutated the site (E42) of Arg1 through point mutation. Co-IP and immunofluorescence results showed a significant reduction in the binding of Arg1 to Mic10 and a noticeably diminished co-localization of fluorescence after the Arg1 (E42A) mutation, compared to the wild-type Arg1 (Arg1^WT^) (Fig. 4a–c), indicating that the two proteins primarily bind through the E42 site. We then observed the effects of the Arg1^E42A^ on the changes in mitochondrial cristae structure. Using transmission electron microscopy and Hessian-SIM super-resolution microscopy, we noted significant improvements in the cristae structure of the VSMCs after hypoxia with the Arg1^E42A^, compared to the Arg1^WT^, with a decrease in vacuole formation and a marked increase in cristae frequency (Fig. 4d, e and Supplementary Fig. S6a, b). Additionally, the Arg1^E42A^ significantly improved OCR, ATP production, inhibited ROS formation, and improved mitochondrial membrane potential in hypoxic VSMCs (Fig. 4f–k). We also found that compared with Arg1^WT^, Arg1^E42A^ effectively reduced the release of mtDNA in hypoxic VSMCs (Fig. 4l–o). We further investigated the impact of the Arg1 E42A mutation on the enzymatic activity of arginase and found that this mutation does not significantly affect enzyme activity (Supplementary Fig. S6c). This further supports the conclusion that the regulatory role of Arg1 in ischemia/hypoxia-induced vascular dysfunction is independent of its enzymatic activity.

### The upregulation of Arg1 and VDAC1 lactylation jointly leads to the release of mtDNA

**Fig. 5:**
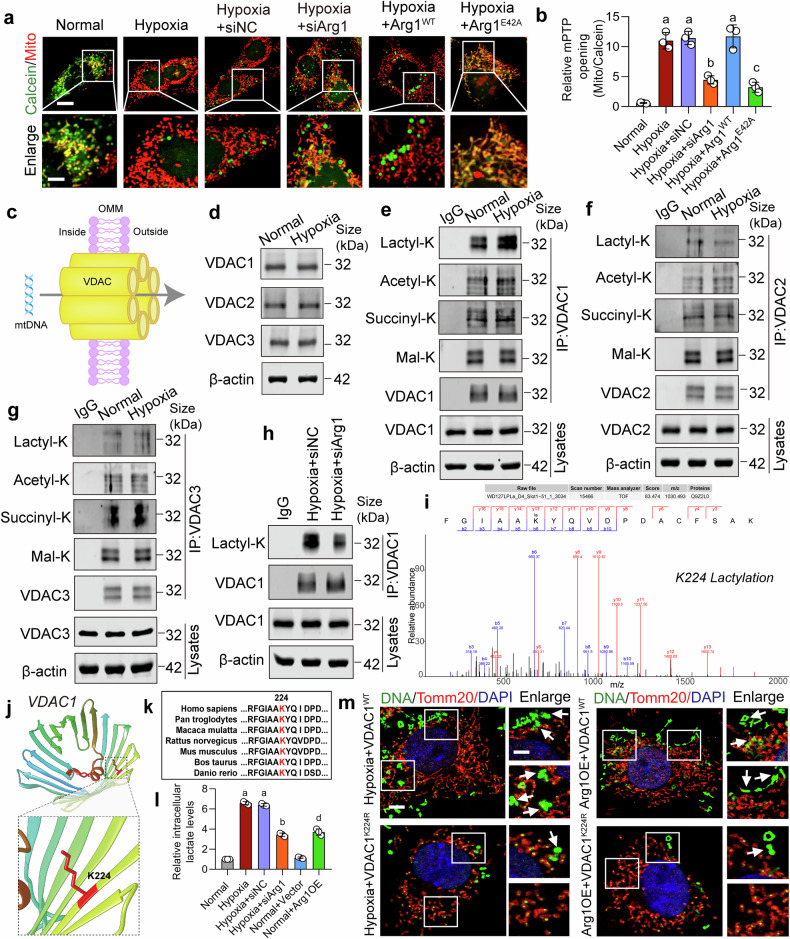
The upregulation of Arg1 and VDAC1 lactylation lead to the release of mtDNA. **a** The extent of mPTP opening was observed using Calcein-CoCl_2_ staining, scale bars correspond to 10 μm for low-magnification images and 5 μm for high-magnification views (n = 3 independent experiments). **b** Relative MPTP opening ratio (fluoresce intensity of MitoTracker/Calcein) in each group (n = 3 independent experiments). **c** Schematic diagram of the spatial relationship between mtDNA and VDAC. **d** Western blot analysis detected the expressions of VDACs following hypoxic conditions (n = 3 independent experiments). **e**–**g** The changes of post-translational modifications of VDACs after hypoxia (n = 3 independent experiments). **h** The effect of Arg1 knockout on VDAC1 lactylation (n = 3 independent experiments). **i** Mass spectrum of VDAC1 lactylation modification sites. **j** VDAC1 lysine 224 (K224) lactylation modification site structural display. **k** Conservational analysis of the K224 site in VDAC1. **l** Relative intracellular lactate levels detection in different groups (n = 3 independent experiments). **m** The effect of VDAC1 K224R mutation mimicking deacetylation on mtDNA release in hypoxic or Arg1 overexpressed VSMCs, scale bars correspond to 5 μm for low-magnification images and 2.5 μm for high-magnification views (n = 3 independent experiments). a: p < 0.05, as compared with the Normal group; b: p < 0.05, as compared with the Hypoxia + siNC group; c: p < 0.05, as compared with the Hypoxia + Arg1^WT^ group; d: p < 0.05, as compared with the Normal + Vector group

The above results showed that Arg1 can affect the cristae structure by binding with Mic10, resulting in the release of mtDNA. Previous studies have found that the mtDNA release is mainly associated with the opening of mPTP.^24,42^ Our results showed that in hypoxic VSMCs, mPTP was significantly opened, and interference with Arg1 or Arg1 E42A mutation inhibited mPTP opening (Fig. 5a, b). Meanwhile, CsA (a specific inhibitor of mPTP) treatment also significantly reduced mtDNA release, similar to the siArg1 group (Supplementary Fig. S7a). These results suggested that Arg1 can cause the opening of mPTP to release mtDNA by affecting the cristae structure. The mPTP is a group of protein complexes located between the inner and outer membranes of the mitochondria.^43^ While the reshaping of cristae can facilitate the translocation of mtDNA across the inner membrane, the release of mtDNA from the mitochondria requires passage through the outer membrane. However, the mechanisms governing the passage of mtDNA through the mitochondrial outer membrane under hypoxic conditions remain to be elucidated.

Prior research suggested that mtDNA exits out of the mitochondria via the VDACs in the outer membrane, as Fig. 5c showed.^24^ To examine the changes in VDACs induced by hypoxia, we assessed the impact of hypoxia on VDACs expression in VSMCs. Our data revealed that the expressions of VDAC isoforms remained unaltered after hypoxia (Fig. 5d and Supplementary Fig. S7b). We investigated the post-translational modifications of VDACs utilizing antibodies and Mass spectrometry. Our findings indicated that only VDAC1 lactylation was significantly increased in response to hypoxia, compared to the normal group (Fig. 5e–g). This may be related to the increased lactate level caused by hypoxia (Fig. 5l). Mass spectrometry identified the lactylation site on VDAC1 as the lysine residue at position 224 (K224), which is highly conserved across species (Fig. 5i–k). We further examined the impact of Arg1 regulation on VDAC1 lactylation. The results indicated that inhibition of Arg1 attenuated VDAC1 lactylation by improving mitochondrial function and partially inhibiting lactate production (Fig. 5h, l). Then we examined the effect of VDAC1 lactylation on mtDNA release and discovered that the K224R mutation in VDAC1, which mimicked the absence of lactylation, inhibited mtDNA release under hypoxic conditions compared to wild-type VDAC1 (Fig. 5m and Supplementary Fig. S7c, d). Besides, overexpression of Arg1 also induced elevated lactate production and mtDNA release, while the VDAC1 K224R mutation appeared to mitigate the effects of Arg1 overexpression (Fig. 5l, m and Supplementary Fig. S7e, f). Furthermore, the inhibition of mitochondrial function using the mitochondrial electron transport chain complex I inhibitor rotenone^44^ also resulted in an increased lactate level similar to the effect observed with Arg1 overexpression (Supplementary Fig. S7g–i). These results indicated that the upregulation of Arg1 and VDAC1 lactylation jointly led to the release of mtDNA.

### mtDNA release triggers PANoptosis in VSMCs via activating cGAS-STING signaling pathway

**Fig. 6:**
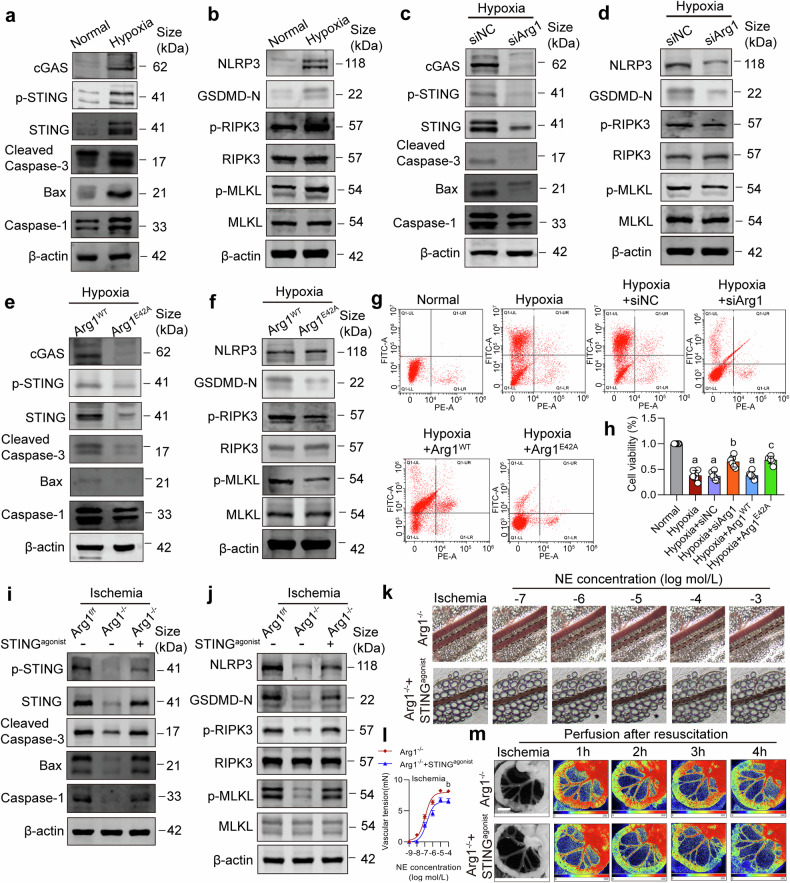
The release of mtDNA induced PANoptosis in VSMCs via activating the cGAS-STING pathway. **a**, **b** Western blot assessed the effects of hypoxia treatment on the expressions of cGAS-STING pathway and PANoptosis-related proteins in VSMCs (n = 3 independent experiments). **c**, **d** Western blot determined the influence of siArg1 on the expressions of cGAS-STING pathway and PANoptosis-related proteins in VSMCs (n = 3 independent experiments). **e**, **f** The influence of the Arg1 E42A mutation on the expressions of cGAS-STING pathway and PANoptosis-related proteins in VSMCs (n = 3 independent experiments). **g** Flow cytometry was utilized to detect apoptosis in cells (n = 3 independent experiments). **h** The viability of VSMCs was measured using the CCK-8 assay (n = 6 independent experiments). **i**, **j** WB detected the impact of STING agonist on PANoptosis-related proteins in SMAs from ischemic Arg1 KO mice (n = 3 independent experiments). **k** The vascular reactivity of SMA upon NE stimulation in STING agonist treated ischemic Arg1 KO mice (n = 6 mice in each group). **l** Variations in SMA diameter reacting to NE concentrations ranging from 10^-7^ to 10^-3 ^mol/L of ischemic Arg1 KO mice treated with STING agonist (n = 6 mice in each group). **m** Laser speckle contrast imaging monitored the intestinal blood flow in ischemic Arg1 KO mice treated with STING agonist (bar = 1 cm) (n = 6 mice in each group). a: p < 0.05, as compared with the Normal group; b: p < 0.05, as compared with the Hypoxia + siNC group or Ischemia + Arg1^-/-^ group; c: p < 0.05, as compared with the Hypoxia + Arg1^WT^ group

Recent studies have found that the release of mtDNA can activate the cGAS-STING signaling pathway, resulting in inflammatory cell damage and inducing various types of programmed cell death, including apoptosis, pyroptosis, and necroptosis.^45,46^ We further explored the mechanisms of released mtDNA leading to vascular dysfunction under ischemia/hypoxia condition. Through WB detection, we found that, the proteins of the cGAS-STING pathway were significantly activated in hypoxic VSMCs compared to normal VSMCs (Fig. 6a and Supplementary Fig. S8a). Furthermore, we measured the expression of apoptosis-related proteins (Cleaved-Caspase-3 and Bax), pyroptosis-related proteins (NLRP3, Caspase-1 and GSDMD-N-terminal), and necroptosis related proteins (p-RIPK3 and p-MLKL). The results showed that the expression of apoptosis, pyroptosis, and necroptosis-related proteins in hypoxia-treated cells were significantly increased compared to the normal group, indicating that hypoxic VSMCs underwent PANoptosis (Fig. 6a, b and Supplementary Fig. S8a). After interfering with Arg1, the activation of the cGAS-STING signaling pathway proteins in hypoxic cells was suppressed, and the expressions of PANoptosis-related proteins were significantly decreased (Fig. 6c, d and Supplementary Fig. S8b). Additionally, we found that Arg1^E42A^ effectively reduced the activation of the cGAS-STING signaling pathway proteins and downregulated the expression of PANoptosis-related proteins, compared to Arg1^WT^ (Fig. 6e, f and Supplementary Fig. S8c). Flow cytometry cell counts and CCK8 results also showed that the proportion of apoptotic cells was significantly increased after hypoxia, and interference with Arg1 or Arg1^E42A^ mutation reduced the proportion of apoptotic cells (Fig. 6g, h). Additionally, knocking out STING downregulated PANoptosis in hypoxic cells (Supplementary Fig. S8d, e). We treated the Arg1^-/-^ mice with STING agonist (1.5 mg/kg) and observed the changes in PANoptosis and vascular function. The results showed that STING agonist effectively countered the protective effect of Arg1 KO on PANoptosis and vascular function of the ischemic mice (Fig. 6i–m and Supplementary Fig. S8f, g). Furthermore, the nNOS inhibitor L-NAME did not induce PANoptosis of VSMCs (Supplementary Fig. S9a, b).

The activation of cGAS-STING promotes TBK1 phosphorylation, which then mediates anti-infection and autoimmune responses.^47^ Through Western blot analysis, we observed that the levels of p-TBK1 were significantly elevated in hypoxia-treated VSMCs compared to the normal group. However, intervention with Arg1 or treatment with the cGAS-STING pathway inhibitor (H-151) effectively suppressed p-TBK1, and the combined use of siArg1 and H-151 did not exhibit an additive effect (Supplementary Fig. S9c, d). These results suggested that mtDNA release triggers PANoptosis via cGAS-STING activation in hypoxic VSMCs.

### Lactate promotes Arg1 expression by upregulating H3K18la

**Fig. 7:**
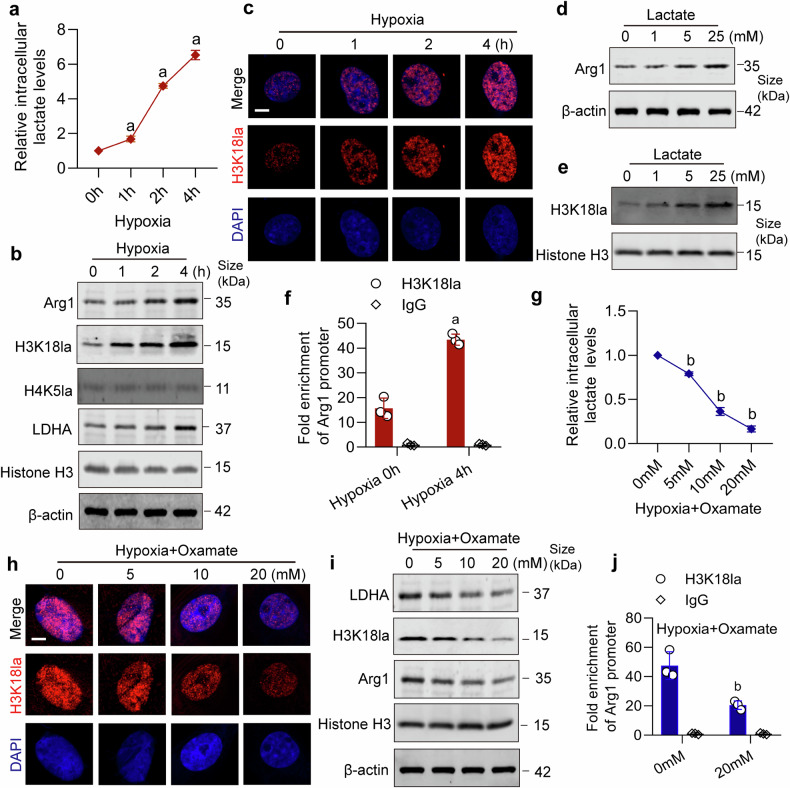
Lactate enhances the expression of Arg1 through the upregulation of H3K18la. **a** Assessment of relative intracellular lactate levels of VSMCs at different time points under hypoxia (n = 3 independent experiments). **b** Western blot analysis of the expressions of Arg1, H3K18la, H4K5la, and LDHA at different time points under hypoxia (n = 3 independent experiments). **c** Immunofluorescence detection of the effect of hypoxia on H3K18la in VSMCs (bar = 5 μm) (n = 3 independent experiments). **d** The effect of lactate stimulation at different concentrations on Arg1 expression (n = 3 independent experiments). **e** The effect of lactate stimulation at different concentrations on H3K18la expression (n = 3 independent experiments). **f** H3K18la occupancy analysis of VSMCs by ChIP–qPCR (n = 3 independent experiments). **g** The relative intracellular lactate levels of VSMCs were treated with different concentrations of Oxamate after hypoxia for 4 hours (n = 3 independent experiments). **h** Immunofluorescence on the effect of different concentrations of Oxamate on H3K18la (bar = 5 μm) (n = 3 independent experiments). **i** The LDHA, H3K18la and Arg1 expressions of VSMCs treated with different concentrations of Oxamate after hypoxia for 4 hours (n = 3 independent experiments). **j** H3K18la occupancy analysis of hypoxic VSMCs after being treated with Oxamate (n = 3 independent experiments). a: p < 0.05, as compared with the 0 h group; b: p < 0.05, as compared with the 0 mM group

The above results indicated that Arg1 influences the release of mtDNA and lactate production. Research has demonstrated that lactate also can regulate gene expression by affecting histone lactylation.^48^ We investigated whether lactate could affect the generation of Arg1. In our hypoxic VSMCs model, we observed a correlation between increased lactate levels and the expression of both Arg1 and H3K18la (Fig. 7a–c and Supplementary Fig. S10a). Direct lactate treatment on VSMCs resulted in the upregulation of Arg1 and H3K18la (Fig. 7d, e and Supplementary Fig. S10b, c). Furthermore, ChIP-qPCR results showed that the binding of H3K18la to the Arg1 promoter increased with prolonged hypoxia (Fig. 7f and Supplementary Fig. S10d–f). Inhibition of lactate production using the LDHA inhibitor Oxamate suppressed the expression of H3K18la and Arg1 (Fig. 7g–i and Supplementary Fig. S10g). Moreover, ChIP-qPCR results indicated that inhibiting lactate synthesis led to a reduction in the binding of H3K18la to the Arg1 promoter (Fig. 7j). These results suggested that lactate accumulation following hypoxia can promote Arg1 expression by upregulating H3K18la.

### Construction of VSMCs-targeting nanoparticle NP-siArg1

**Fig. 8:**
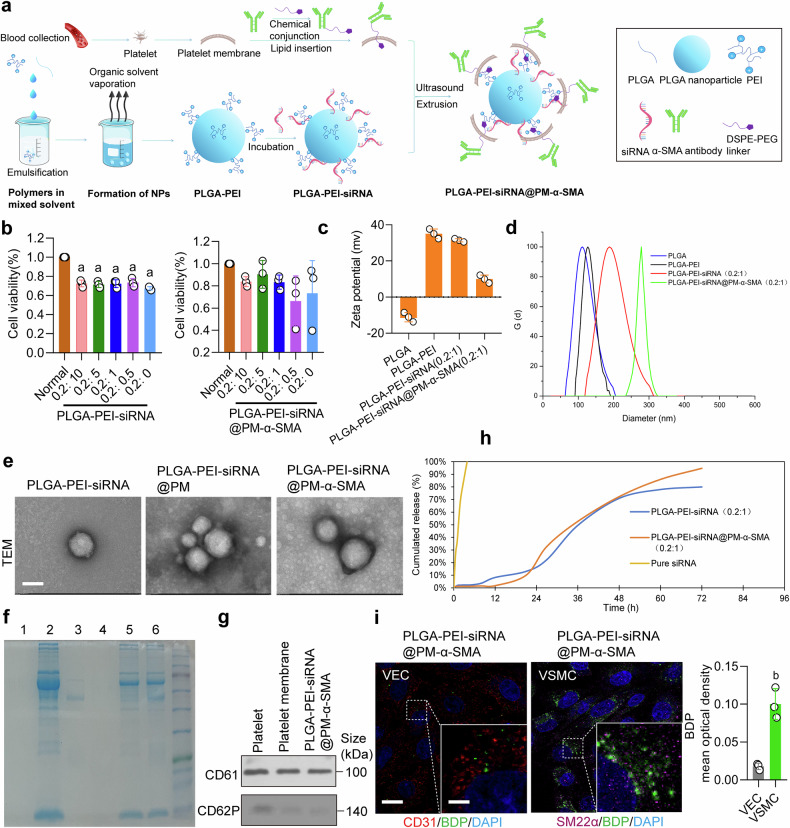
Characterization and detection of nanoparticles. **a** A schematic of PLGA-PEI-siRNA@PM-α-SMA (NP-siArg1) synthesis. **b** Effect of different ratios of siRNA in PLGA-PEI and PLGA-PEI@PM-α-SMA nanoparticles on cell viability (n = 3 independent experiments). **c** Zeta potential diagrams of nanoparticles with different components. **d** Particle size distribution diagrams for nanoparticles with different components. **e** Transmission electron microscopy images of PLGA-PEI-siRNA, PLGA-PEI-siRNA@PM, and PLGA-PEI-siRNA@PM-α-SMA (bar = 100 nm). **f** SDS-PAGE of PLGA (No.1), PM (No.2), α-SMA monoclonal antibody (No.3), PLGA-PEI-siRNA (No.4), PLGA-PEI-siRNA@PM (No.5), PLGA-PEI-siRNA@PM-α-SMA (No.6). **g** WB detection of the expressions of CD61 and CD62P. **h** In vitro release curves of siRNA, PLGA-PEI-siRNA, and PLGA-PEI-siRNA@PM-α-SMA. **i** Targeting specificity of PLGA-PEI-siRNA@PM-α-SMA to VSMCs versus VECs. VECs were labeled with CD31, VSMCs with SM22α, and nanoparticles with BDP, scale bars correspond to 25 μm for low-magnification images and 10 μm for high-magnification views (n = 3 independent experiments). a: p < 0.05, as compared with the Normal group; b: p < 0.05, as compared with the VEC group

To explore the clinical translation potential of Arg1 as a treatment target for vascular dysfunction, we constructed a nanoparticle (NP) loaded with Arg1 siRNA (NP-siArg1). To target VSMCs, we coated the surface of PLGA (Poly (DL-lactide-co-glycolide acid)) nanoparticles with platelet membrane (PM). Additionally, we modified the platelet membrane with α-SMA to enhance targeting of VSMCs. We also introduced PEI to increase the loading efficiency of siRNA into the nanoparticles. PEI increases the loading efficiency of siRNA to nanoparticle surfaces through electrostatic absorption, as shown in Fig. 8a. Table 1 presented the results for particle size, zeta potential, and polydispersity index (PDI) of the nanoparticles produced in this experiment. High encapsulation rates indicated that the chosen conditions were efficient for siRNA packaging, and the siRNA/PEI complexes are not on the nanoparticle surface.^49^ Therefore, after the incorporation of antibodies and platelet membrane, PLGA-PEI surfaces carried a positive charge, but this charge gradually neutralized with the addition of siRNA. We evaluated cytotoxicity, interference efficiency testing, particle size, and zeta potential across different ratios. The molar ratio of 0.2:1 (PLGA:siRNA) was selected based on its low cytotoxicity and optimal interference efficiency (Fig. 8b and Supplementary Fig. S11a, b). We then measured the changes in particle size and zeta potential of nanoparticles after PM coating and antibody conjugation. The particle size increased from 153.41 nm to 284.97 nm, while the zeta potential decreased from 35.05 mV to 9.98 mV (Fig. 8c, d). These results showed that antibodies, siRNA, and PM were effectively encapsulated within the nanoparticles. The spherical structure of the nanoparticles was confirmed using transmission electron microscopy. After PM coating, a layer resembling a spread membrane formed on the surface of the nanoparticles (Fig. 8e). SDS identifies the presence of cell membranes and antibodies on the surface of nanoparticle, WB identifies the presence of platelet membranes inside the nanoparticle (Fig. 8f, g).

**Table 1:** siRNA composite nanoparticle size, zeta potential, and PDI

Nanoparticle compositional components	Particle size (nm)	Zeta potential(mv)	PDI	Production efficiency	Encapsulation rate
PLGA	99.93 ± 10.01	−11.12 ± 2.58	0.174 ± 0.026		
PLGA@PEI	153.41 ± 4.60	35.05 ± 2.68	0.165 ± 0.029		
PLGA@PEI-siRNA (molar ratio 0.2:10)	209.70 ± 5.53	34.27 ± 6.60	0.217 ± 0.020	31.21%	96.25%
PLGA@PEI-siRNA (molar ratio 0.2:5)	214.90 ± 9.52	32.50 ± 2.34	0.190 ± 0.030	35.76%	93.47%
PLGA@PEI-siRNA (molar ratio 0.2:1)	209.15 ± 14.58	31.45 ± 0.90	0.165 ± 0.013	39.42%	92.45%
PLGA@PEI-siRNA (molar ratio 0.2:0.5)	213.21 ± 5.01	22.14 ± 5.81	0.145 ± 0.03	42.45%	91.46%
PLGA@PEI@PM-α-SMA	254.88 ± 11.41	15.75 ± 2.81	0.299 ± 0.02		
PLGA@PEI-siRNA@PM-α-SMA (molar ratio 0.2:10)	237.64 ± 5.27	11.81 ± 0.40	0.101 ± 0.050	34.65%	92.12%
PLGA@PEI-siRNA@PM-α-SMA (molar ratio 0.2:5)	248.29 ± 3.80	10.56 ± 4.99	0.202 ± 0.055	36.82%	90.25%
PLGA@PEI-siRNA@PM-α-SMA (molar ratio 0.2:1)	284.97 ± 9.48	9.98 ± 2.17	0.133 ± 0.11	40.61%	89.37%
PLGA@PEI-siRNA@PM-α-SMA (molar ratio 0.2:0.5)	306 ± 69.42	8.75 ± 4.02	0.338 ± 0.03	44.21%	90.78%

In vitro release experiments revealed that the siRNA solution released 56% within 1.5 hours in 200 mL of medium and completely released within 4 hours. For PLGA-PEI-siRNA nanoparticles, only 2% siRNA release was observed within the initial 8 hours. After 8 hours, the release of siRNA gradually increased, reaching 50% at 36 hours. In the subsequent 36 hours, the fast-release phase entered a second phase. Within 72 hours, the cumulative release rate reached 80%. However, for the PLGA-PEI-siRNA@PM-α-SMA nanoparticle group, the cumulative release rate of siRNA was only 2% at 12 hours. At 21 hours, as the nanoparticles came in full contact with the PBS in the dialysis bag, water quickly entered into the nanoparticles, thereby accelerating the release of surface siRNA. The entire process took about 27 hours. During the second phase, gradual hydrolysis of the polymer resulted in a slower release of siRNA, with the release rate increasing from 33% to 95% (Fig. 8h). Cell phagocytosis experiment indicated that PLGA-PEI-siRNA@PM-α-SMA was effectively phagocytosed by VSMCs (Supplementary Fig. S11c). These results indicated the successful synthesis of VSMCs-targeting nanoparticles: PLGA-PEI-siRNA@PM-α-SMA (NP-siArg1).

We conducted dose screening for PLGA-PEI-siRNA@PM-α-SMA. The results demonstrated that doses ranging from 1 to 500 μg/mL effectively suppressed Arg1 expression; however, cytotoxicity increased with higher doses. Consequently, 1 μg/mL was selected to balance efficacy and safety (Supplementary Fig. S11d–f). Finally, we incubated PLGA-PEI-siRNA@PM-α-SMA with vascular endothelial cells (VECs) and VSMCs to evaluate its targeting specificity. Results demonstrated that VSMCs exhibited significantly higher nanoparticle uptake efficiency than VECs, confirming its VSMC-targeting capability (Fig. 8i). Since hypoxia increases the endothelial permeability,^50^ we further assessed nanoparticle penetration through VEC monolayers to VSMCs using the transwell assay (VECs in the upper chamber, VSMCs in the lower chamber). Compared to the normal group, the hypoxia group showed markedly increased nanoparticle uptake (Supplementary Fig. S11g–i), indicating enhanced penetration under hypoxic conditions. The results indicate the successful construction of a VSMC-targeting nanoparticle, NP-siArg1.

### Therapeutic effect of VSMCs-targeting nanoparticle NP-siArg1 on ischemic/hypoxic injury-induced vascular dysfunction

**Fig. 9:**
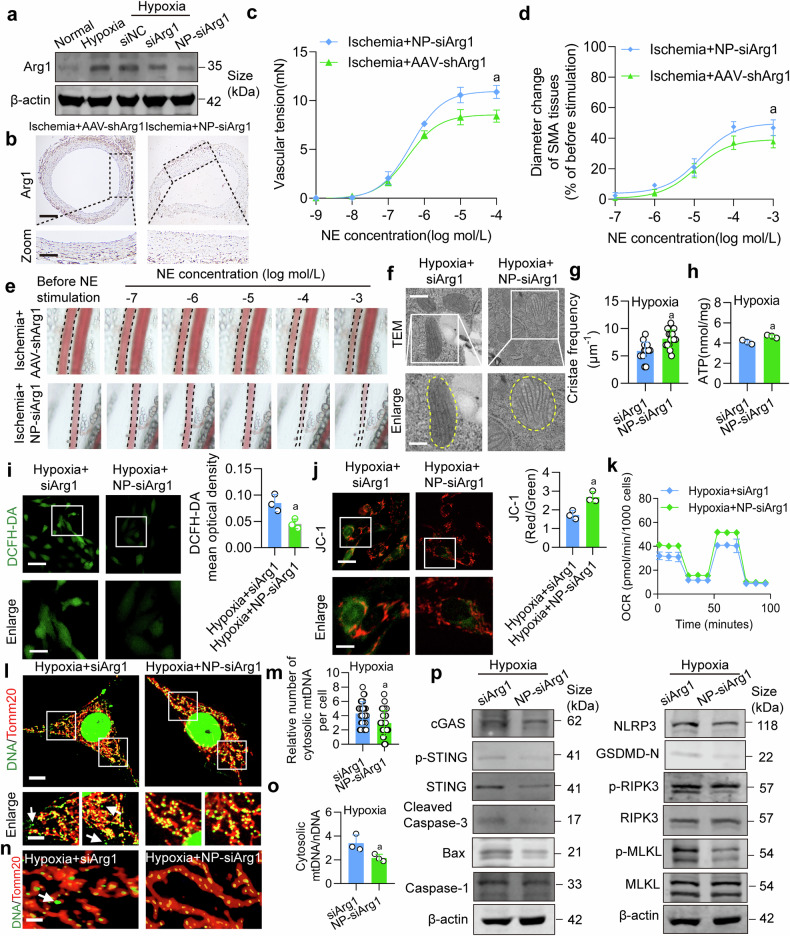
The therapeutic effects of NP-siArg1 on ischemic/hypoxic injury-induced vascular dysfunction. **a** The effects of NP-siArg1 on the expression of Arg1 in VSMCs under hypoxic condition (n = 3 independent experiments). **b** Immunohistochemistry determined the impact of NP-siArg1 on Arg1 expression in SMA tissue (n = 6 rats in each group) (100X). **c** The vascular reactivity of SMA tissues upon NE stimulation in NP-siArg1-treated rats was quantified (n = 6 rats in each group). **d** The statistics of diameter change in SMA reacting to NE stimulation (n = 6 rats in each group). **e** Changes in SMA diameter in response to NE concentrations ranging from 10^-7^ to 10^-3 ^mol/L were measured to evaluate the effect of NP-siArg1 (n = 6 rats in each group). The consequences of NP-siArg1 on **f**, **g** mitochondrial cristae structure, scale bars correspond to 300 μm for low-magnification images and 150 μm for high-magnification views, **h** ATP level, **i** ROS production, scale bars correspond to 60 μm for low-magnification images and 30 μm for high-magnification views, **j** mitochondrial membrane potential, scale bars correspond to 40 μm for low-magnification images and 20 μm for high-magnification views, and **k** mitochondrial OCR were assessed (n = 3 independent experiments). **l**, **m** The consequences of NP-siArg1 on mtDNA release, scale bars correspond to 10 μm for low-magnification images and 5 μm for high-magnification views (n = 3 independent experiments). **n** Confocal three-dimensional layer scanning on mtDNA release by (bar = 1 μm) (n = 3 independent experiments). **o** The ratio of mtDNA/nDNA in VSMCs (n = 3 independent experiments). **p** The effect of NP-siArg1 on hypoxia-induced PANoptosis in VSMCs (n = 3 independent experiments). a: p < 0.05, as compared with the Ischemia + AAV-shArg1 or Hypoxia + siArg1 group

We further evaluated the therapeutic efficacy of NP-siArg1. Compared to siArg1 or AAV-shArg1, the NP-siArg1 (3 mg/kg)^51,52^significantly reduced the expression of Arg1 (Fig. 9a, b and Supplementary Fig. S12a). Moreover, compared to AAV-shArg1, NP-siArg1 exhibited more pronounced improvements in vascular reactivity in ischemic rats (Fig. 9c–e). Additionally, NP-siArg1 also showed superior efficacy in improving the survival rate of ischemic rats compared to AAV-shArg1 (Supplementary Fig. S12b, c). We additionally examined the effects of NP-siArg1 on the mitochondrial cristae structure and function, mtDNA release, and PANoptosis in hypoxic VSMCs. The results revealed that, compared to siArg1, NP-siArg1 improved the mitochondrial cristae structure in hypoxic VSMCs, showing a significant increase in mitochondrial cristae frequency (Fig. 9f, g). It inhibited ROS generation, increased ATP level, OCR, and mitochondrial membrane potential, as shown in Fig. 9h–k. Figure 9l–p and Supplementary Fig. S12d–f illustrated that NP-siArg1 exhibited better inhibition of mPTP opening, mtDNA release, and PANoptosis in hypoxic VSMCs compared to siArg1. These findings demonstrated that inhibition of Arg1 is a novel intervention strategy to alleviate ischemic/hypoxic injury-induced vascular dysfunction, with potential clinical implications for translational application.

## Discussion

**Fig. 10:**
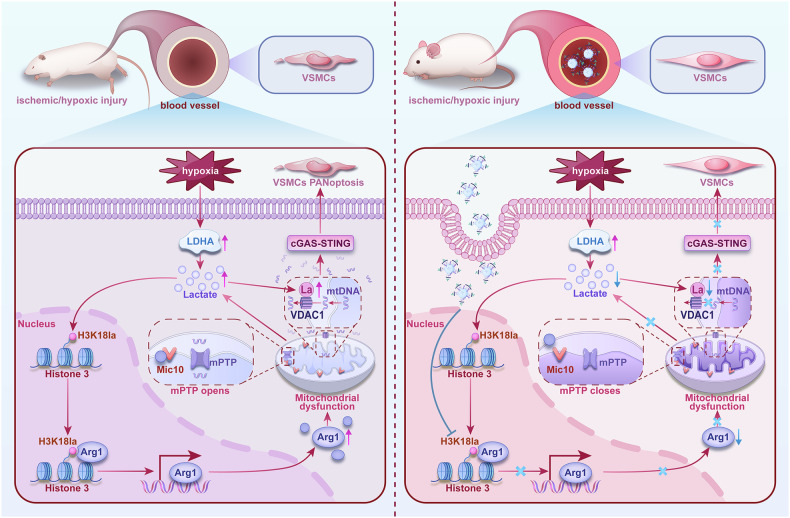
A schematic diagram of ischemic/hypoxic injury-induced vascular dysfunction and targeted therapy

Our study indicates that ischemic/hypoxic injury can induce marked vascular dysfunction, disorganization of mitochondrial cristae, mitochondrial dysfunction and release of mtDNA. The primary mechanism involves augmented lactate generation subsequent to the ischemic/hypoxic injury, and increased expression of H3K18la in VSMCs, which promotes Arg1 expression. The heightening of Arg1 expression intensifies its interaction with Mic10, leading to the structural remodeling of Mic10 that finally disrupts the structure of mitochondrial cristae. The dysfunction of mitochondria augments lactate production in VSMCs. This process forms a positive feedback loop of damage signaling between the nucleus and mitochondria. Moreover, the elevated lactate leads to VDAC1 K224 lactylation. The mitochondrial cristae disorder and VDAC1 lactylation jointly lead to mtDNA release. Ultimately, the release of mtDNA activates the cGAS-STING signaling pathway, triggering PANoptosis in VSMCs. VSMCs targeting nanoparticle to intervene the expression of Arg1 in VSMCs can effectively improve vascular dysfunction via blocking mitochondrial cristae remodeling and mtDNA release (Fig. 10).

We found that ischemic/hypoxic injury can lead to vascular hyporeactivity. Previous studies have shown that vascular hyporeactivity is associated with adrenergic receptor desensitization, calcium desensitization, and membrane hyperpolarization.^3,53^ The mechanism mainly involves the regulation of RhoA, Rac1, and MLCK activity within VSMCs.^54^ However, the mechanisms do not fully explain the features like mitochondrial cristae remodeling and mtDNA release induced by ischemia/hypoxia. The finding that Arg1-mediated mitochondrial cristae remodeling and VSMC PANoptosis provide new insights into ischemic/hypoxic injury-induced vascular dysfunction at the organelle level. nNOS is known to be expressed in VSMCs,^55^ and L-arginine serves as a substrate for nNOS-dependent nitric oxide production. However, we found that the levels of nitric oxide and nNOS expression in SMA did not change significantly after ischemia. This could be due to the nitrite (NO_2_^–^) reduction pathway, which produces NO through enzymatic or non-enzymatic reactions, compensating for potential fluctuations in NOS-dependent NO.^56^ Additionally, nNOS activity in VSMCs is regulated by calcium signaling and chaperone proteins. Ischemia may maintain its function by activating calmodulin-dependent pathways,^57^ but the specific mechanisms need further investigation in the future.

Mic10, also termed Min10, Mcs10, or Mos1, resides in mitochondrial inner membrane. Recent studies indicate that Mic10 functions as a pivotal component of the MICOS complex, essential for sustaining normal mitochondrial operations and cristae structure.^16,58^ Mic10 has two transmembrane domains with termini protruding into the mitochondrial intermembrane space, and each transmembrane region is characterized by an abundance of conserved glycine sequences GxGxGxG. Emerging evidence suggests that mutations disrupting the GxGxGxG sequences hinder the inner membrane’s capacity to bend. It appears that oligomers of Mic10 are strategically positioned on either side of cristae junctions, facilitating the inner membrane’s extension towards the matrix and consequently shaping the mitochondrial cristae.^15,16^ The downregulation of Mic10 diminishes the number of cristae junctions, modifies cristae architecture, and results in concentric circular formations. In contrast, Mic10 overexpression extends cristae abnormally.^59^ Thus, Mic10’s role in the MICOS complex is crucial for preserving cristae morphology in mitochondria. Furthermore, investigations have revealed Mic10’s involvement in mtDNA distribution, potentially near cristae junctions. Under expression of Mic10 may obliterate these junctions, severing cristal connections to the inner membrane and impairing mtDNA association with mitochondrial cristae.^23,59^ This study revealed that hypoxia in VSMCs enhances the interaction between Arg1 and Mic10, inhibits Mic10 homo-oligomerization, and promotes the release of mtDNA. Recent findings suggest that only the trimeric form of Arg1 is enzymatically active,^60^ highlighting the crucial role of Arg1’s oligomeric state in its functional regulation. Our study examines the interaction interface between the Arg1 monomer and Mic10 at the E42 site. However, the Arg1 trimeric conformation may influence E42’s accessibility due to steric hindrance, thereby affecting the binding efficiency with Mic10. Future research should focus on elucidating the structure of the Arg1 trimer-Mic10 complex to provide a more comprehensive structural basis for understanding the mechanism.

The nuclear-mitochondrial communication is a key process to maintain cellular metabolic and physiological homeostasis. Recent studies have highlighted the role of metabolic products in this cross-membrane communication between nucleus and mitochondria. Lactate has been considered a waste product of cellular metabolism, Newer evidence suggests that lactate plays an important role in the communication between the nucleus and mitochondria.^61^ Research by Hashimoto et al. found that after lactate enters the nucleus, it regulates the expression of genes related to mitochondrial function through chromatin remodeling.^62^ The accumulation of lactate can also impact the function of cells through histone lactylation.^48^ For instance, histone lactylation promotes the activation of reparative genes following a myocardial infarction.^63^ The results of this study suggest that lactate can act as a nuclear-mitochondrial signaling molecule. We discovered that lactate regulated the expression of Arg1 through histone lactylation (H3K18la). Arg1, in turn, interacted with Mic10, disrupting the structure of mitochondrial cristae and further promoting lactate generation, which formed a positive feedback loop of damaging signals between the nucleus and mitochondria. Additionally, lactate directly induced lysine 224 lactylation of the VDAC1. While Arg1 primarily affected the inner mitochondrial membrane, VDAC1 lactylation mainly impacted the outer mitochondrial membrane. Together, these effects caused mPTP opening, leading to mtDNA release. Notably, the influence of Arg1 on lactate could promote the lactylation of VDAC1. Our findings revealed that lactate functioned not only as an energy source in the cytoplasm but also as a key metabolic signal involved in the communication network between mitochondria and the nucleus, this is consistent with previous studies.^64,65^

PANoptosis, a recently identified form of programmed cell death, exhibits features of necroptosis, apoptosis, and pyroptosis.^66^ Emerging evidence suggests a critical role of PANoptosis in the pathogenesis of critical disease-associated multiorgan failure.^67^ Apoptosis is mediated by caspases and relies on the activation of caspase-3.^68^ Necroptosis is a form of regulated cell death that requires the activation of RIPK3 and MLKL.^69^ Pyroptosis is an inflammatory form of cell death triggered by inflammasomes, culminating in gasdermin cleavage and the formation of membrane pores.^70^ Studies have shown that endoplasmic reticulum stress associated with STING activation can lead to BAX activation and the release of cytochrome c, inducing apoptosis.^71^ Moreover, activation of the cGAS-STING pathway can result in the production of IFN and TNF. The binding of TNF to TNFR1 causes the activation of RIPK1/RIPK3 which, in the absence of Caspase-8 inhibition, leads to the phosphorylation of MLKL and necroptosis.^46^ Additionally, cGAS-STING-mediated IFN production can enhance the activity of AIM2, thereby increasing the expression of Caspase-1 and promoting pyroptosis.^72^ Consistent with previous studies, our research has found that the conformation change of Mic10 leading to mitochondrial cristae remodeling can activate cGAS-STING signaling pathway by releasing mtDNA, further triggering the activation of caspase-3, MLKL, and GSDMD, which induced PANoptosis in VSMCs and contribute to vascular dysfunction.

This study has some limitations. This study found that Arg1-mediated mitochondrial damage can promote lactate production, forming a positive feedback loop of damaging signals. However, this loop may be cross-regulated with the HIF1α pathway. On one hand, lactate can stabilize HIF1α,^73^ on the other hand, mitochondrial ROS may activate HIF1α.^74^ The synergy between these mechanisms might be key to pathological progression. The experimental framework of this study focuses on the mechanism by which the interaction between Arg1 and Mic10 in VSMCs regulates mitochondrial cristae remodeling and contributes to vascular dysfunction. However, it is noteworthy that Arg1 in endothelial cells has been shown to participate in inducing endothelial dysfunction.^75,76^ Future studies should employ cell-specific knockout models and functional assays to further elucidate the mechanisms by which Arg1 in endothelial cells influences vascular dysfunction, as well as the synergistic effects of endothelial-VSMC interactions in vascular dysfunction. This study found that the release of mtDNA can activate p-TBK1. The upregulation of p-TBK1 may regulate inflammatory responses through the activation of transcription factors such as IRF3 or NF-κB.^77^ However, its specific downstream targets in PANoptosis have not yet been elucidated. Additionally, TBK1 may interact with other stress signals to co-regulate cell death, which requires further investigation.

In summary, this study reveals a new mechanism by which ischemic/hypoxic injury induces vascular dysfunction. Lactate regulates the expression of Arg1, forming a positive feedback loop of damaging signals between the nucleus and mitochondria, leading to the disorganization of mitochondrial cristae and the release of mtDNA. This process ultimately results in PANoptosis in VSMCs. Furthermore, the use of nanotechnology to target VSMCs with siArg1 effectively mitigates vascular dysfunction. Our findings provide new insights into the role of mitochondria in organ damage and a new therapeutic strategy for ischemic/hypoxic injury at the sub-cellular level.

## Materials and methods

### Ethical approval of the study protocol

The animal experiments adhered to the guidelines of Reporting of In Vivo Experiments (ARRIVE). The experiment protocol was approved by the Laboratory Animal Welfare and Ethics Committee of the Army Medical University (approval No. AMUWEC20224867).

### Reagents

Antibodies for Arg1 (ab233548), Bax (ab32503), and NLRP3 (ab263899) were purchased from Abcam (Cambridge, MA, USA). β-actin (ab8226), Tubulin (ab7291), and COX IV (ab202554), also obtained from Abcam, were used as internal references for total, cytoplasmic, and mitochondrial fractions, respectively. Antibodies for Mic10 (GTX46630), CD31 (GTX74899) and Arg2 (GTX118048) were purchased from GeneTEX (San Antonio, TX, USA). Antibodies for Cleaved-Caspase-3 (9664S) and were SM22α (40471S) purchased from Cell Signaling Technology (Danvers, Massachusetts, USA). Antibodies for DNA (NB110-89473) and Tomm20 (NBP1-81556) were purchased from Novus (Briarwood Avenue, Centennial, USA). Antibodies for LDHA (A23678), VDAC1 (A19707), VDAC2 (A16294), VDAC3 (A10544), cGAS (A8335), phospho-STING (AP1223), STING (A21051), Caspase-1 (A21085), GSDMD (A24476), MLKL (A21894), phospho-MLKL (AP1174), RIPK3 (A5431), and phospho-RIPK3 (AP1257) were purchased from ABclonal (Wuhan, China). Antibodies for Lactyl-Histone H3 (Lys18) (PTM-1427RM), Lactyl-Histone H4 (Lys5) (PTM-1407RM), Histone H3 (PTM-1002RM), Lactyl-lysine (PTM-1401RM), Malonyl-lysine (PTM-902), Acetyl-lysine (PTM-101) and Succinyl-lysine (PTM-419) were obtained from PTM Biolabs (Hangzhou, China). Antibodies for nNOS (bsm-52474R), phospho-TBK1 (bs-3440R), and TBK1(bsm-60750R) were obtained from Bioss (Beijing, China). DCFH-DA (S0033M), JC-1 (C2003S), MPTP (C2009S), and Nitric oxide (S0021S) assay kits were purchased from Beyotime Biotechnology (Shanghai, China). Lactate assay kit (L256) was purchased from Dojindo (Kyushu, Japan). Urea assay kit (ab83362) was purchased from Abcam (Cambridge, MA, USA). L-Arginine assay kit (E-BC-K850-M) was purchased from Elabscience (Wuhan, China). L-NAME (HY-18729A), L-Arginine (HY-N0455), Urea (HY-Y0271), Rotenone (HY-B1756), STING agonist (HY-143321) and inhibitor (HY-112693) were purchased from MedChemExpress (Monmouth, NJ, USA). Arginase activity assay kit (MAK112) was purchased from Sigma (St. Louis, MO, USA). CsA (S2286) was purchased from Selleck (Shanghai, China). Adenoviral vectors for Arg1 overexpression (Arg1OE), Arg1 (E42A) mutation, VDAC1 (K224R) mutation, STING knockdown (shSTING) and Adeno-associated virus for Arg1 knockdown (AAV-shArg1) were generated by Genechem Technology (Shanghai, China). siRNA for Arg1 was generated by Sangon Biotech (Shanghai, China). All other chemicals were purchased from Sigma unless specifically mentioned.

### Establishment of an ischemic rat model

Before the experiment, the rats were fasted for eight hours with free access to water. They were given sodium pentobarbital (30 mg/kg) and placed on a heated platform to keep their body temperature at 37 °C. All surgical procedures were performed under aseptic conditions. Hemodynamic monitoring was conducted by catheterizing the right carotid artery and vein. Additionally, the right femoral artery was catheterized to withdraw approximately 50% of the total blood volume ( ≈ 7% of body weight) over a 10-minute period, and the ischemic state was maintained for 4 hours. After the experiment, the rats were euthanized with a lethal dose of sodium pentobarbital (100 mg/kg, iv).^9,78^

### Construction of Arg1 VSMCs conditional knockout C57BL/6J mouse

Arg1-flox mice (NM-CKO-200062) were purchased from the Shanghai Model Organisms Center, Inc. LoxP sites were inserted flanking exon 7-8 of the Arg1 gene, and a conditional Arg1 gene knockout mouse model was established using STOCK Tg (Tagln-cre)1Her/J mice (JAX stock #004746).^79^ These transgenic mice express Cre recombinase under the control of the mouse transgelin (Tagln, also known as smooth muscle protein 22-alpha) promoter. When crossbred with a strain harboring a loxP site-flanked gene segment, Cre-mediated recombination facilitates the excision of the targeted sequence in VSMCs.

### Cell culture and hypoxic cell model

VSMCs were harvested from the superior mesenteric arteries (SMAs) of Sprague-Dawley rats utilizing an explant method as described previously.^9^ After obtaining the VSMCs, they were cultured in the Dulbecco-Modified Eagle Medium (DMEM)-F12, supplemented with 10% Fetal Bovine Serum (FBS) and 1% antibiotics. Cells from the third to fifth passage were used for the following experiments. VSMCs were cultured in low-glucose DMEM (1000 mg/L D-glucose) without FBS and moved into a hypoxia culture compartment, filled with a gas mixture of 95% N_2_ and 5% CO_2_, in which the oxygen concentration was estimated to be less than 0.2%. After 4 hours of hypoxia, the cell model preparation was complete.

### Vascular reactivity measurement

The vascular reactivity of SMA to norepinephrine (NE) was assessed using an isolated organ perfusion system (Scientific Instruments, Barcelona, Spain). Each SMA was cut into 1-2 mm length, suspended between a force transducer, and immersed into an isolated organ chamber (Scientific Instruments, Barcelona, Spain) containing 5 ml Krebs–Henseleit (K-H) solution. The vascular contractile response of the arterial rings to a gradient of NE concentrations (10^-9^ to 10^-4 ^mol/L) was determined by a Power Lab System interfaced with a force transducer.

To observe the vasoconstriction of SMAs in vivo, a midline incision (20-30 mm) was made to open the abdomen. The ileocecal part of the mesentery was carefully extirpated and situated on a transparent plastic stage. Single unbranched arterioles without conspicuous bends (diameters ranging from 30 to 50 μm and approximately 200 μm) were selected. The mesentery was maintained at 37°C and sufficiently hydrated by continuous superfusion with a saline solution. Post-equilibrium for 10 minutes, graded doses of NE (10^-7^ to 10^-3 ^mol/L) in a volume of 100 μL were added to the mesenteric surface. This caused the arterioles to constrict, and the alterations in the diameter were digitally captured using a video camera (Olympus, DP21).

### siRNA transfection

The VSMCs were grown to 30-50% confluence. The siRNA primer was diluted to 50 nM in 50 µL of Opti-MEM and pipetted gently 3-5 times. It was then mixed with 1 µL of Lipofectamine2000 and diluted in another 50 µL of Opti-MEM. After the mixture was incubated at room temperature for 5 minutes, it was combined and further incubated for 20 minutes. The final 50 nM transfection complex was added to the culture dish, which was then placed in a 37 °C, 5% CO_2_ incubator for 72 hours. The target sequence was as follow: Arg1 siRNA, 5′- GGAUGAGCAUGAGCUCCAATT-3′.

### Transmission electron microscopy

Adhering to the established protocol,^10^ the treated VSMCs were collected to observe the mitochondrial cristae by Hitachi H-7500 transmission electron microscope (TEM). Fifteen mitochondria were selected from each group to observe the frequency of cristae. Mitochondrial cristae frequency (the cristae amount of unit length of mitochondria) was calculated by ImageJ.

### Immunofluorescence staining

VSMCs were dispersed in a confocal chamber, rinsed twice with 1X PBS, and then fixed with 4% paraformaldehyde at room temperature for 20 minutes. The cells were permeabilized with 0.1% Triton-X 100 in 1X PBS for 5 minutes and blocked with 5% BSA at room temperature for 1 hour. They were then incubated overnight at 4 °C with primary antibodies targeting Tomm20 or DNA. After rinsing with PBS containing 0.1% Tween-20, the cells were incubated with fluorophore-conjugated secondary antibodies (mouse or rabbit) from Invitrogen (Carlsbad, CA, USA) for 1 hour at room temperature, then rinsed once more with 1X PBS. The immunofluorescence staining was observed using a confocal laser scanning microscope (Leica SP5, Germany).

### mPTP opening detection

The activation of mPTP was assessed using Calcein-CoCl_2_ staining and confocal microscopy. The treated VSMCs were incubated for 30 minutes with 2 μM Calcein and 100 nM MitoTracker DeepRed, followed by washing with PBS and exposure to 2 mM CoCl_2_ for 15 minutes. The Calcein fluorescence commonly localizes within the mitochondria, only experiencing distribution throughout the cobalt when mPTP opening allows for such, subsequently dimming the Calcein fluorescence found within the mitochondrial matrix. After three PBS washes, fluorescence imaging was performed at 488 nm excitation (Calcein) or 633 nm (MitoTracker), and emission at 510–550 nm (Calcein) or 560–617 nm (MitoTracker) using Leica TCS software.

### Cytosolic mtDNA isolation

After lysis, VSMCs were centrifuged at 700 × g for 10 minutes at 4 °C to remove the nuclei. The supernatant was normalized based on protein concentration. The cell lysate was then centrifuged at 10,000 × g for 30 minutes at 4 °C to isolate the cytosolic fraction, including mtDNA and nDNA. Quantitative PCR using primers for mitochondrial cytochrome c oxidase 1 (mtCOI) and b-globin detected mtDNA and nDNA.^80,81^ The mtDNA to nDNA ratio was calculated to assess mtDNA release into the cytoplasm. Primers are listed in Supplementary Table S2.

### Real-time quantitative PCR

Total RNA was extracted using Trizol Reagent (Invitrogen, NY, USA). PrimeScript RT Master Mix (Takara, Shiga, Japan) was utilized to synthesize cDNA via reverse transcription. Real-time quantitative PCR was performed using FastStart Essential DNA Green Master (Roche, Shanghai, China). GAPDH served as the reference gene, and relative gene expression was calculated using the 2^-ΔΔCt method.

### Subcellular fractionation

VSMCs were collected using filter cartridges. The cytosolic fractions were isolated with a Minute™ Cytoplasmic Extraction Kit (Invent Biotechnologies, Inc. SC-003), while the mitochondrial fractions were obtained using the Minute™ Mitochondria Isolation Kit (Invent Biotechnologies, Inc. MP-007).^43^ The fractionated proteins were used for immunoblotting analyses.

### Molecular dynamics

Molecular dynamics (MD) simulation was performed employing Gromacs software version 2021.03 to explore the interaction between protein Arg1 and Mic10. The simulation commenced with the docking of Arg1 to Mic10, utilizing the Amber99SB force field, following the procedure detailed in a previously published article.^6^ Visualization of the simulation outcomes was performed using Origin 8.0 software.

### Identification of post-translational modification sites in proteins

For in-gel tryptic digestion, gel slices were destained with 50% acetonitrile in 50 mM NH4HCO3, dehydrated with 100% acetonitrile, reduced with 10 mM dithiothreitol at 37 °C for 60 minutes, and alkylated with 55 mM iodoacetamide in the dark at room temperature for 45 minutes. After washing and rehydration in 50 mM NH_4_HCO_3_ with 10 ng/μL trypsin, the slices were incubated on ice for 1 hour, then digested overnight at 37 °C. Peptides were extracted with 50% acetonitrile and 5% formic acid, followed by 100% acetonitrile, lyophilized, reconstituted in solvent A, and analyzed using an EASY-nLC 1000 UHPLC coupled to a Thermo Scientific™ Q Exactive mass spectrometer with NSI ionization. MS/MS data were searched with Proteome Discoverer 1.3 using the following parameters: Trypsin/P enzyme; max 4 missed cleavages; carbamidomethyl as a fixed modification; methionine oxidation, protein N-terminal acetylation, serine, threonine, tyrosine phosphorylation, lysine acetylation, malonylation, succinylation, and lactylation as variable modifications; peptide ion score threshold >20; High peptide confidence for results.

### Synthesis of PLGA-PEI-siRNA

30 mg PLGA was dissolved in 1 mL dichloromethane. siRNA was mixed with PEI, vortexed, and incubated for 30 minutes. The PEI-siRNA solution was combined with the PLGA solution, dripped into 4 ml of 5% PVA, stirred, and ultrasonicated to form an emulsion. This emulsion was added to 0.3% PVA, stirred, washed, and ultrafiltered to obtain PLGA-PEI-siRNA nanoparticles. Finally, BDP-FL-NHS ester (1:1) was conjugated to the nanoparticles.

### Preparation of platelet membrane-wrapped PLGA-PEI-siRNA (PLGA-PEI-siRNA@PM)

Rat whole blood was collected in sodium citrate tube. Platelet-rich plasma was obtained by centrifugation at 200 g for 8 minutes, followed by 4000 g for 5 minutes. The platelet membrane was extracted through five freeze-thaw cycles (−80 °C to room temperature), lyophilized, and stored at −80 °C. Membrane proteins were quantified using the Bradford method. The membrane was mixed with PLGA-PEI-siRNA (1:1 mass ratio) and ultrasonicated at 42 kHz (ice bath, 5 seconds on/off for 5 minutes). Unencapsulated membrane was removed by ultrafiltration, and the sample was fluorescently labeled.^82,83^

### Preparation of PLGA-PEI-siRNA@PM-α-SMA (NP-siArg1)

DSPE-PEG2000-NHS and anti-α-SMA antibody were mixed in PBS (pH 6.5), stirred for 24 hours, ultrafiltered, lyophilized, and stored at −20°C. Platelet membranes were incubated with anti-α-SMA at 37°C for 30 minutes, washed, ultrafiltered, and combined with PLGA-PEI-siRNA (1:1 protein mass ratio). The mixture was sonicated at 42 kHz in an ice bath, centrifuged, and ultrafiltered to remove unencapsulated membranes.

### Encapsulation and production efficiency

The siRNA content in the nanoparticles was quantified using RiboGreen, and the amounts of siRNA added and in the wash solutions were detected separately with a fluorometer. The calculation formula for encapsulation efficiency is as follows:

$${\rm{Encapsulation}}\; {\rm{efficiency}}=\frac{({{\rm{siRNA}}}_{{\rm{t}}}-{{\rm{siRNA}}}_{{\rm{w}}})}{{{\rm{siRNA}}}_{{\rm{t}}}}\times 100\%$$

${{\rm{siRNA}}}_{{\rm{t}}}$ is the total amount of the siRNA added, ${{\rm{siRNA}}}_{{\rm{w}}}$ is the amount of siRNA in the wash solution.

The produced PLGA-PEI-siRNA was lyophilized using a freeze-dryer, then its mass was measured, and the production efficiency was calculated according to the following formula:

$${\rm{Production}}\; {\rm{efficiency}}=\frac{{{\rm{W}}}_{{\rm{dried}}\; {\rm{NP}}}}{{{\rm{W}}}_{{\rm{siRNA}}}+{{\rm{W}}}_{{\rm{PLGA}}}}\times 100\%$$

${{\rm{W}}}_{{\rm{dried\; NP}}}$ is the mass of the nanoparticles after freeze-drying, ${{\rm{W}}}_{{\rm{siRNA}}}$ is the mass of siRNA, ${{\rm{W}}}_{{\rm{PLGA}}}$ is the mass of the PLGA that was added.

### Particle size, potential, and morphology analysis

Samples were diluted with enzyme-free water before analysis. Nanoparticle size and Zeta potential were measured using a NanoBrook Omni analyzer. Morphology was assessed via transmission electron microscopy by depositing nanoparticles on a copper grid, blotting after two minutes, and staining with uranyl acetate. Target platelet membrane proteins were identified by Western Blot.

### Release ratio of siRNA

siRNA-loaded nanoparticles were placed in a dialysis bag and stirred in PBS (pH 7.4) at 37 °C for 4 days. Samples were centrifuged at 15,000 g for 30 minutes, and the supernatant was mixed 1:1 with Ribogreen RNA assay reagent, incubated in the dark for 3 minutes, and analyzed for fluorescence intensity. The release rate was calculated using the following formula:

$${\rm{Release}}\; {\rm{ratio}}=\frac{{\rm{The}}\; {\rm{amount}}\; {\rm{of}}\; {\rm{siRNA}}\; {\rm{released}}}{{\rm{The}}\; {\rm{amount}}\; {\rm{of}}\; {\rm{siRNA}}\; {\rm{added}}}\times 100 \%$$

### Statistical analysis

Statistical analyses were performed using SPSS (version 20.0) and GraphPad Prism (version 8.0). All data are presented as mean ± standard deviation (SD). Animal experiments were conducted with at least 6 biological replicates, while cell experiments were performed in three independent experiments. Discrepancies between two groups were analyzed with independent t-tests. For comparison for more than two groups, ANOVA followed by Tukey’s test, SNK, or LSD tests were used. Statistical significance was set at P < 0.05.

## Supplementary information

Sigtrans_Supplementary_Materials

Original images of Western blots

Author Checklist for Research Article (20250219)

## Data Availability

All data needed to evaluate the conclusions in the article are present in the article and the Supplementary Materials. The data and materials used in the current study are available from the corresponding authors upon reasonable request. The RNA sequencing data analyzed in this publication have been deposited in NCBI’s Gene Expression Omnibus and are accessible through GEO Series accession number GSE64711. The mass spectrometry proteomics data have been deposited to the ProteomeXchange Consortium via the PRIDE partner repository with the dataset identifier PXD063156 (https://www.ebi.ac.uk/pride/).
