# Supplementary material for: Arginase 1 drives mitochondrial cristae remodeling and PANoptosis in ischemia/hypoxia-induced vascular dysfunction
Source: Signal Transduct Target Ther. 2025 May 28;10:167. doi: 10.1038/s41392-025-02255-2 (PMC12117058; doi:10.1038/s41392-025-02255-2)
Supplement: Supplementary file 1 — Sigtrans_Supplementary_Materials [file 41392_2025_2255_MOESM1_ESM.docx]

Supplementary Materials for

**Arginase 1 drives mitochondrial cristae remodeling and PANoptosis in ischemia/hypoxia-induced vascular dysfunction**

Han She^1,2†^, Jie Zheng^3†^, Guozhi Zhao^4†^, Yunxia Du^1†^, Lei Tan^1^, Zhe-Sheng Chen^5^, Yinyu Wu^1^, Yong Li^1^, Yiyan Liu^2^, Yue Sun^1^, Yi Hu^1^, Deyu Zuo^6,7*^, Qingxiang Mao^1*^, Liangming Liu^2*^, and Tao Li^2*^

Correspondence to: lt200132@tmmu.edu.cn

**This PDF file includes:**

Figures. S1 to S12

Tables. S1 to S2

**Other Supplementary Materials for this manuscript include the following:**

Original images of Western blots

***Supplementary Figures S1-S12***

**
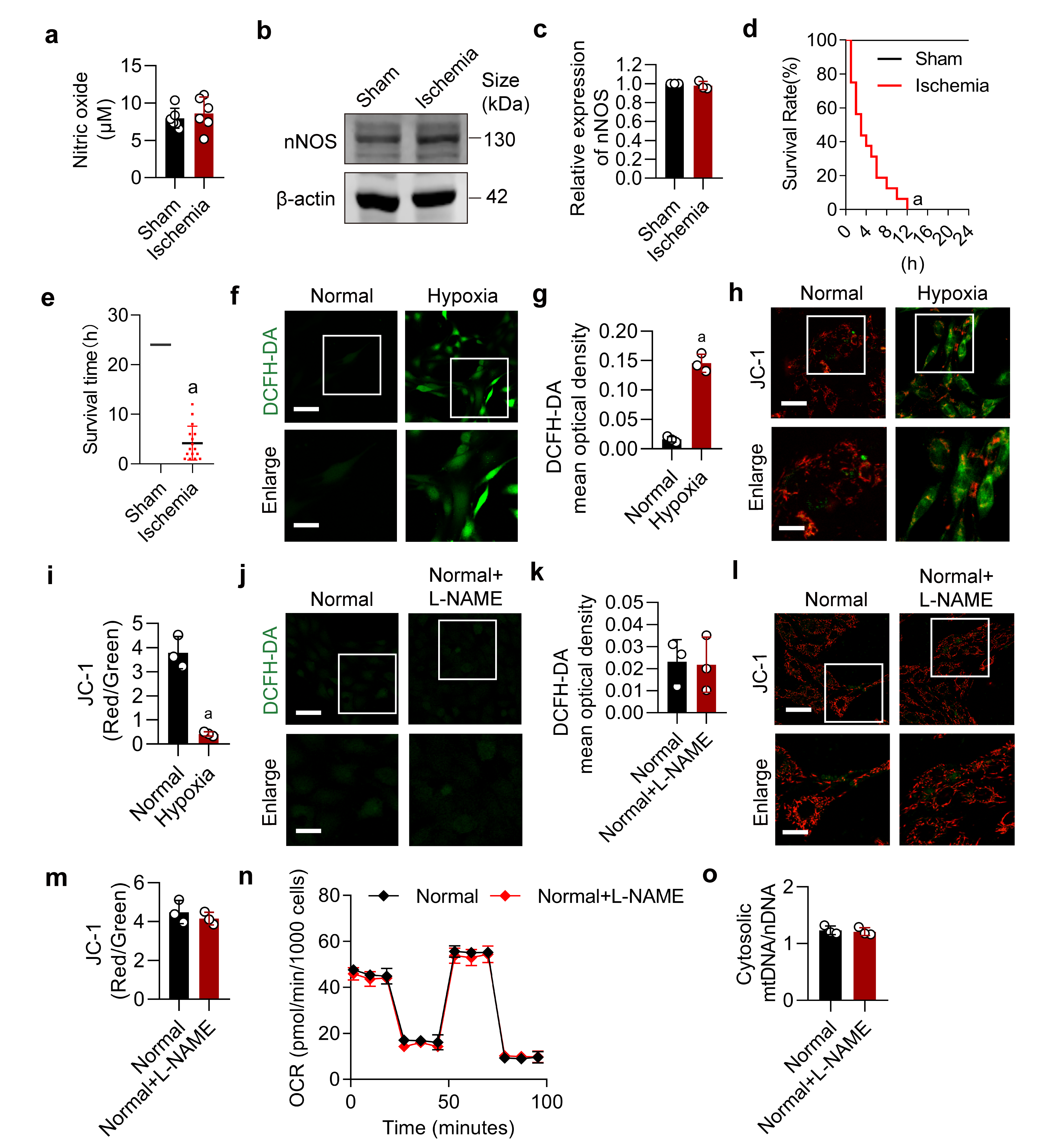
**

**Figure. S1.**

**a** Measurement of nitric oxide levels in ischemic rats (n=6 rats in each group). **b-c** Western Blot analysis of nNOS levels (n=3 independent experiments). **d** The 24-hour survival rate of ischemic rats (n=16 rats in each group). **e** The 24-hour survival time for ischemic rats (n=16 rats in each group). **f-g** ROS levels were determined by DCFH-DA, with mean fluorescence intensity assessed via ImageJ, scale bars correspond to 60 μm for low-magniﬁcation images and 30 μm for high-magniﬁcation views (n=3 independent experiments). **h-i** JC-1 was used to evaluate mitochondrial membrane potential, with membrane potential inferred from the ratio of red to green fluorescence, as determined by ImageJ, scale bars correspond to 40 μm for low-magniﬁcation images and 20 μm for high-magniﬁcation views (n=3 independent experiments). **j-k** The effect of L-NAME (100μM) on ROS levels, scale bars correspond to 60 μm for low-magniﬁcation images and 30 μm for high-magniﬁcation views (n=3 independent experiments). **l-m** The effect of L-NAME on mitochondrial membrane potential, scale bars correspond to 40 μm for low-magniﬁcation images and 20 μm for high-magniﬁcation views (n=3 independent experiments). **n** The effect of L-NAME on mitochondrial OCR in VSMCs (n=3 independent experiments). **o** The effect of L-NAME on the ratio of mtDNA/nDNA in VSMCs (n=3 independent experiments). a: p < 0.05, as compared with the Sham or Normal group.


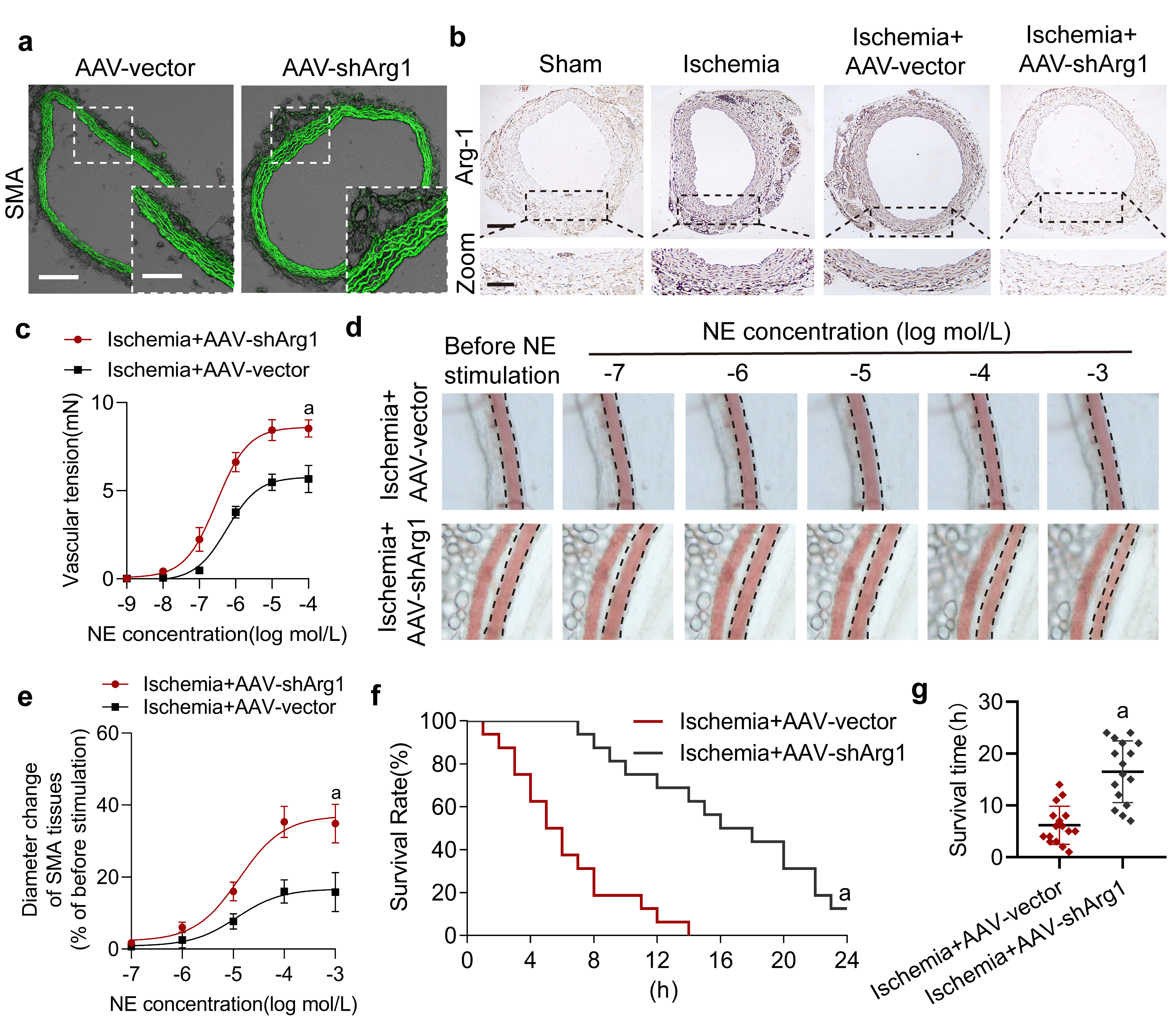


**Figure. S2.**

**a** The infection efficiency of AAVs in frozen SMA tissue sections was assessed (The rats were injected with the AAVs (1.0×10^12^vg, per rat) through the tail vein), scale bars correspond to 200 μm for low-magniﬁcation images and 100 μm for high-magniﬁcation views (n=6 rats in each group). **b** Immunohistochemistry evaluated the knockdown efficiency of AAVs on Arg1 expression in SMA tissues, scale bars correspond to 200 μm for low-magniﬁcation images and 100 μm for high-magniﬁcation views (n=6 rats in each group). **c** Quantification of the vascular reactivity of SMA tissues upon NE stimulation in shArg1-modified samples (n=6 rats in each group). **d** The influence of shArg1 on the variations of SMA diameter in response to NE concentrations ranging from 10^-7^ to 10^-3^ mol/L (n=6 rats in each group). **e** The statistics of diameter change in SMA reacting to NE stimulation (n = 6 rats in each group). The impact of shArg1 on the **f** 24-hour survival rate and **g** survival time of ischemic rats (n=16 rats in each group). a: p<0.05, as compared with the Ischemia+AAV-vector group.


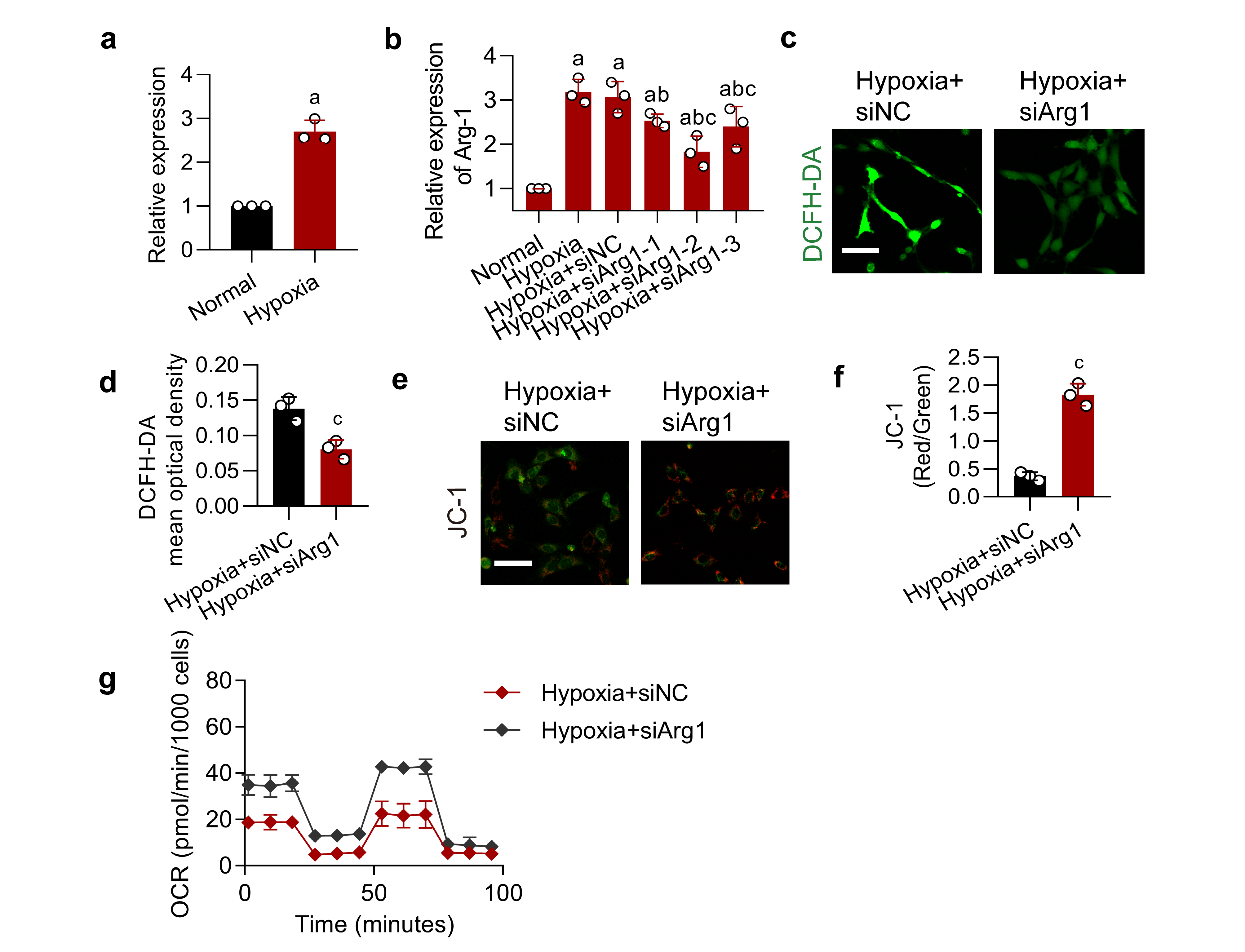


**Figure. S3.**

**a** Relative expression of Arg1 in VSMCs following hypoxia (n=3 independent experiments). **b** The interference efficiency of siRNA (n=3 independent experiments). The effects of siArg1 on the **c-d** ROS (bar=60μm) and **e-f** mitochondrial membrane potential (bar=60 μm) (n=3 independent experiments). **g** Mitochondrial OCR in VSMCs determined by the Seahorse analyzer (n=3 independent experiments). a: p<0.05, as compared with the Normal group; b: p<0.05, as compared with the Hypoxia group; c: p<0.05, as compared with the Hypoxia+siNC group.


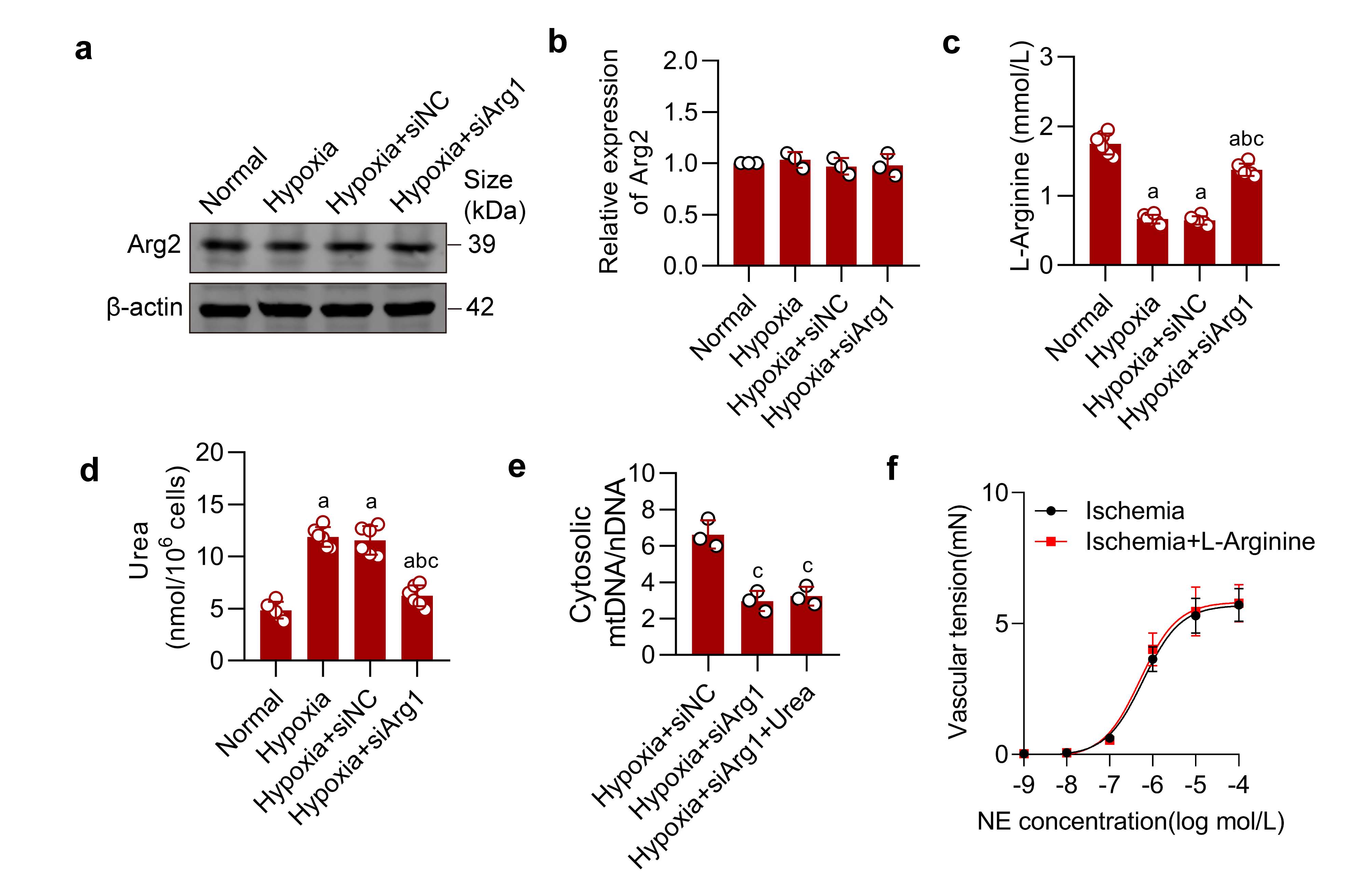


**Figure. S4.**

**a-b** Western blot analysis was used to detect the relative expression of Arg2 (n=3 independent experiments). **c** L-arginine level measurement (n=6 independent experiments). **d** Urea level measurement (n=6 independent experiments). **e** The effect of urea (10 mM) on mtDNA release from hypoxia-treated VSMCs with Arg1 interference (n=3 independent experiments). **f** The impact of L-arginine (300 mg/kg, iv) supplementation on vascular reactivity to NE stimulation in the SMA tissues of ischemic rats (n=6 rats in each group). a: p<0.05, as compared with the Normal group; b: p<0.05, as compared with the Hypoxia group; c: p<0.05, as compared with the Hypoxia+siNC group.


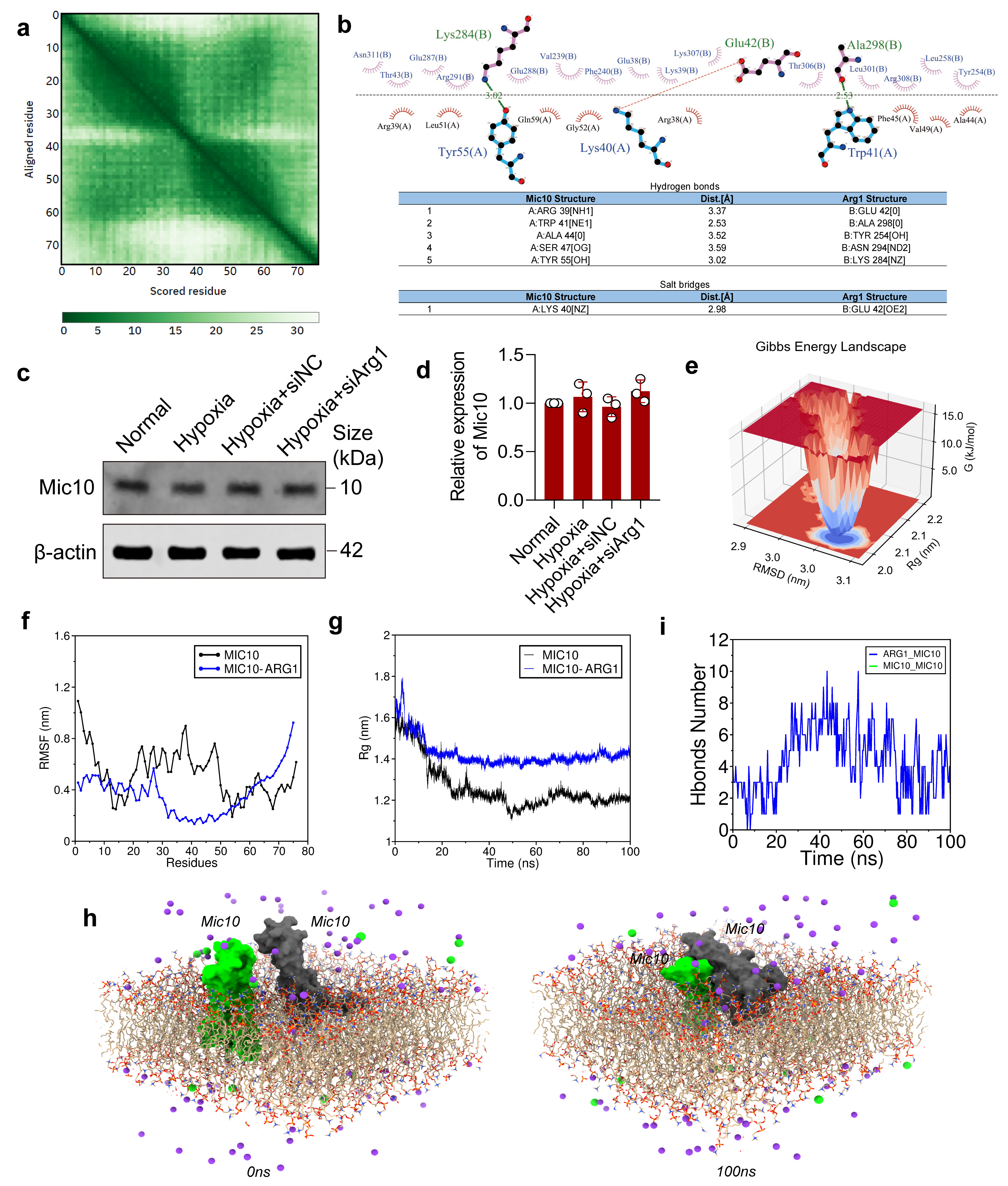


**Figure. S5.**

**a** AlphaFold evaluated the prediction results with high confidence. **b** Molecular docking compared the binding affinities at various binding sites. **c-d** Western blot detected the relative expression of Mic10 (n=3 independent experiments). **e** The Gibbs free energy landscape. **f** Root Mean Square Fluctuation (RMSF) analysis indicating significant changes in Mic10's structural stability upon interaction with Arg1(E42). **g** The radius of gyration (Rg) demonstrated a marked increase in the volume and conformation of Mic10 following its engagement with Arg1(E42). **h** Mic10 monomers can spontaneously polymerize within the membrane. **i** Formation of hydrogen bonds.


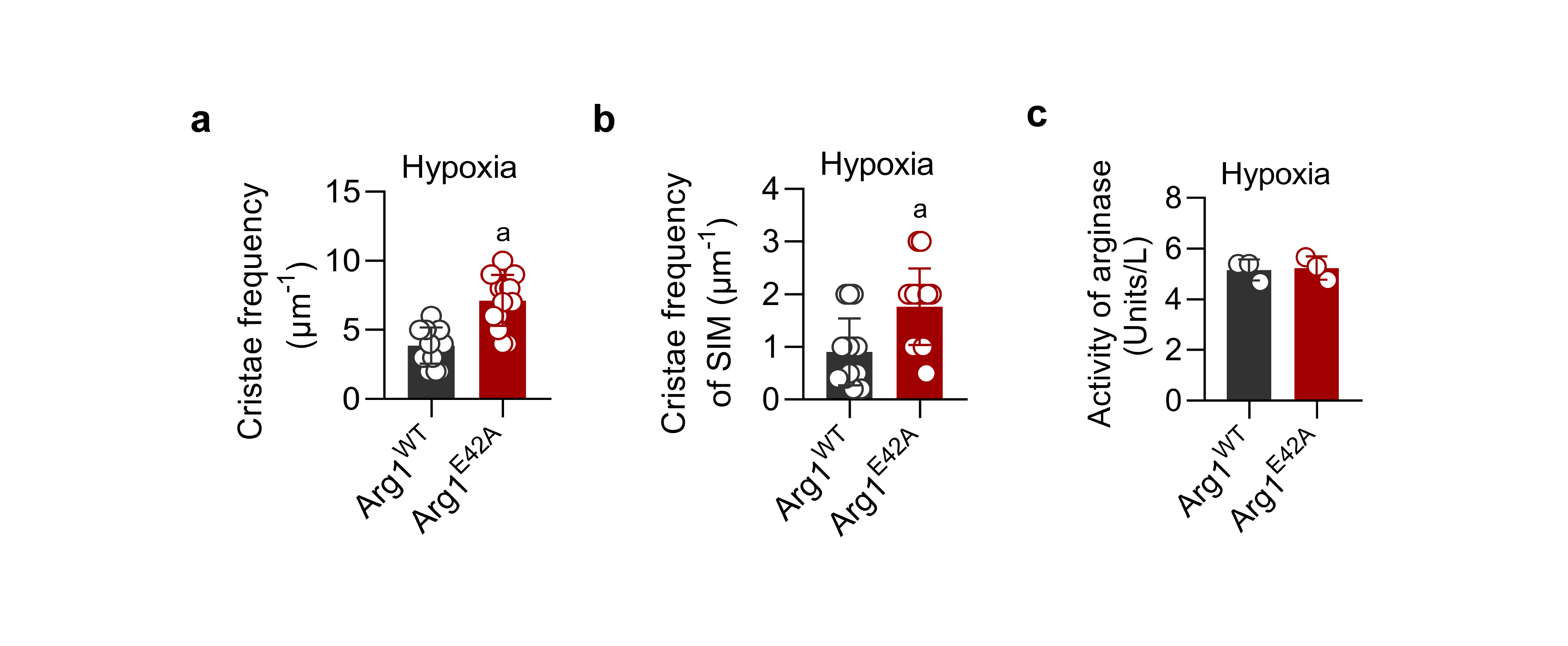


**Figure. S6.**

**a** The cristae frequency was statistically analyzed by examining 15 randomly chosen mitochondria, calculating cristae frequency as the number of cristae per mitochondrial length unit. Data represent three independent experiments. **b** The cristae frequency detected by Hessian-SIM super-resolution microscopy was statistically analyzed by examining 15 randomly chosen mitochondria. Data represent three independent experiments. **c** Arginase activity assay of Arg1 E42A mutation. Data represent three independent experiments. a: p<0.05, as compared with the Hypoxia+Arg1^WT^ group.


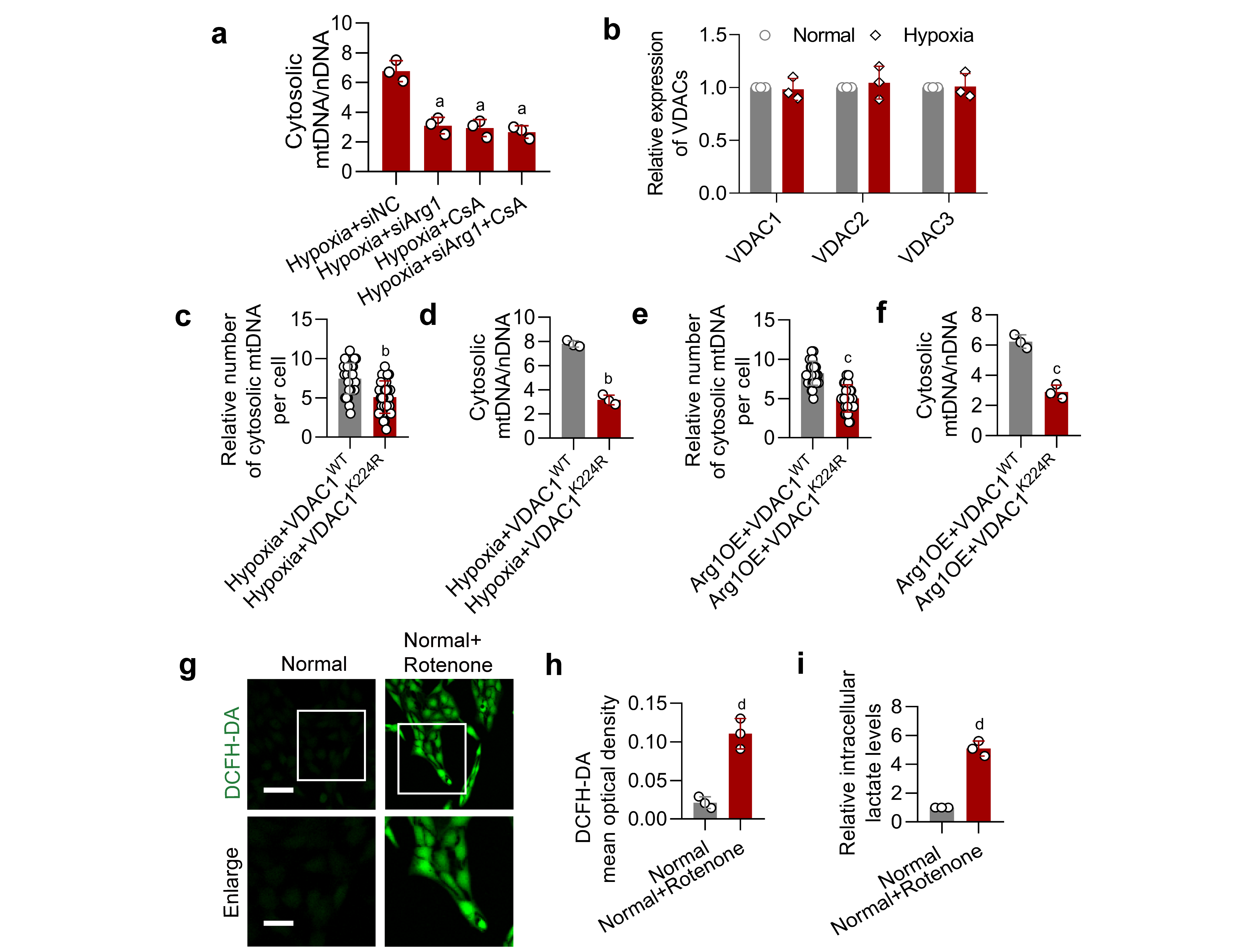


**Figure. S7.**

**a** Ratio of mtDNA/nDNA in CsA (10 μM) treated VSMCs under hypoxic conditions (n=3 independent experiments). **b** Relative expressions of VDACs (n=3 independent experiments). **c** The number of cytosolic mtDNA puncta per cell as quantitated in hypoxia treated VDAC1^K224R^ VSMCs (n=30 cells/group). Data represent three independent experiments. **d** Ratio of mtDNA/nDNA in hypoxia-treated VDAC1^K224R^ VSMCs (n=3 independent experiments). **e** The number of cytosolic mtDNA puncta per cell as quantitated in Arg1 overexpression (Arg1OE) treated VDAC1^K224R^ VSMCs (n=30 cells per group). Data represent three independent experiments. **f** The ratio of mtDNA/nDNA in Arg1OE treated VDAC1^K224R^ VSMCs (n=3 independent experiments). **g-h** The effect of mitochondrial inhibitor rotenone on ROS production in VSMCs, scale bars correspond to 60 μm for low-magniﬁcation images and 30 μm for high-magniﬁcation views (n=3 independent experiments). VSMCs were treated with 1µM rotenone for 2 h. **i** The effect of rotenone on relative intracellular lactate levels in VSMCs (n=3 independent experiments). a: p<0.05, as compared with the Hypoxia+siNC group; b: p<0.05, as compared with the Hypoxia+VDAC1^WT^ group; c: p<0.05, as compared with the Arg1OE+VDAC1^WT^ group; d: p<0.05, as compared with the Normal group.


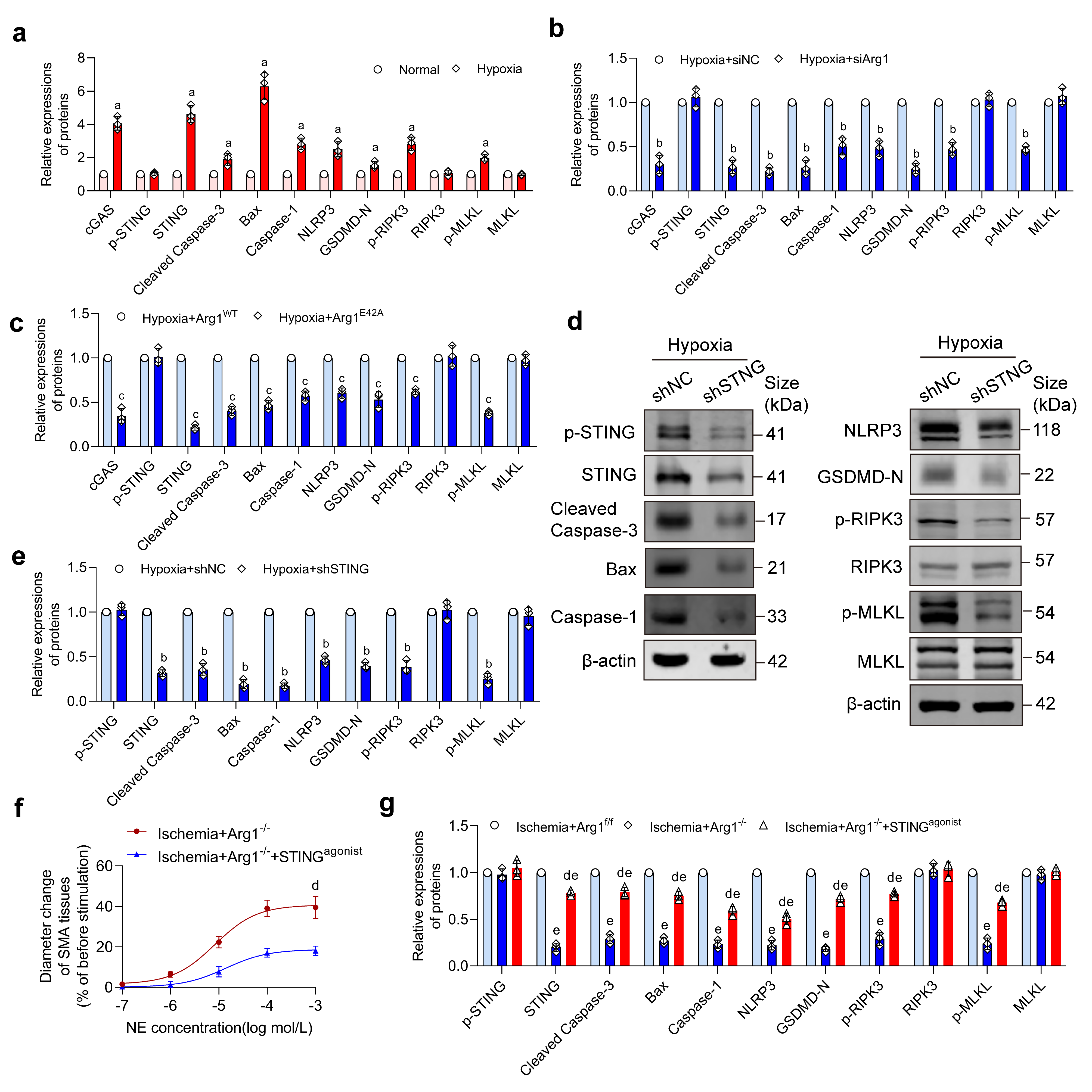


**Figure. S8.**

**a** Relative expressions of cGAS-STING and PANoptosis-related proteins in VSMCs. cGAS, STING, Cleaved Caspase-3, Bax, Caspase-1, NLRP3, GSDMD-N, RIPK3, and MLKL were normalized to β-actin, while p-STING, p-RIPK3, and p-MLKL were normalized to their corresponding total proteins (n=3 independent experiments). **b** Relative expressions of cGAS-STING and PANoptosis-related proteins in siArg1 treated VSMCs (n=3 independent experiments). **c** Relative expressions of cGAS-STING and PANoptosis-related proteins in VSMCs with Arg1 E42A mutation (n=3 independent experiments). **d-e** Western blot assessed the effects of STING knockout on the expressions of PANoptosis-related proteins in VSMCs (n=3 independent experiments). **f** The statistics of diameter change in SMA reacting to NE stimulation (n=6 mice in each group). **g** Relative expressions of PANoptosis-related proteins in SMAs from ischemic Arg1CKO mice (n=3 independent experiments). a: p<0.05, as compared with the Normal group; b: p<0.05, as compared with the Hypoxia+siNC or Hypoxia+shNC group; c: p<0.05, as compared with the Hypoxia+Arg1^WT^ group; d: p<0.05, as compared with the Ischemia+Arg1^-/-^ group; e: p<0.05, as compared with the Ischemia+Arg1^f/f^ group.


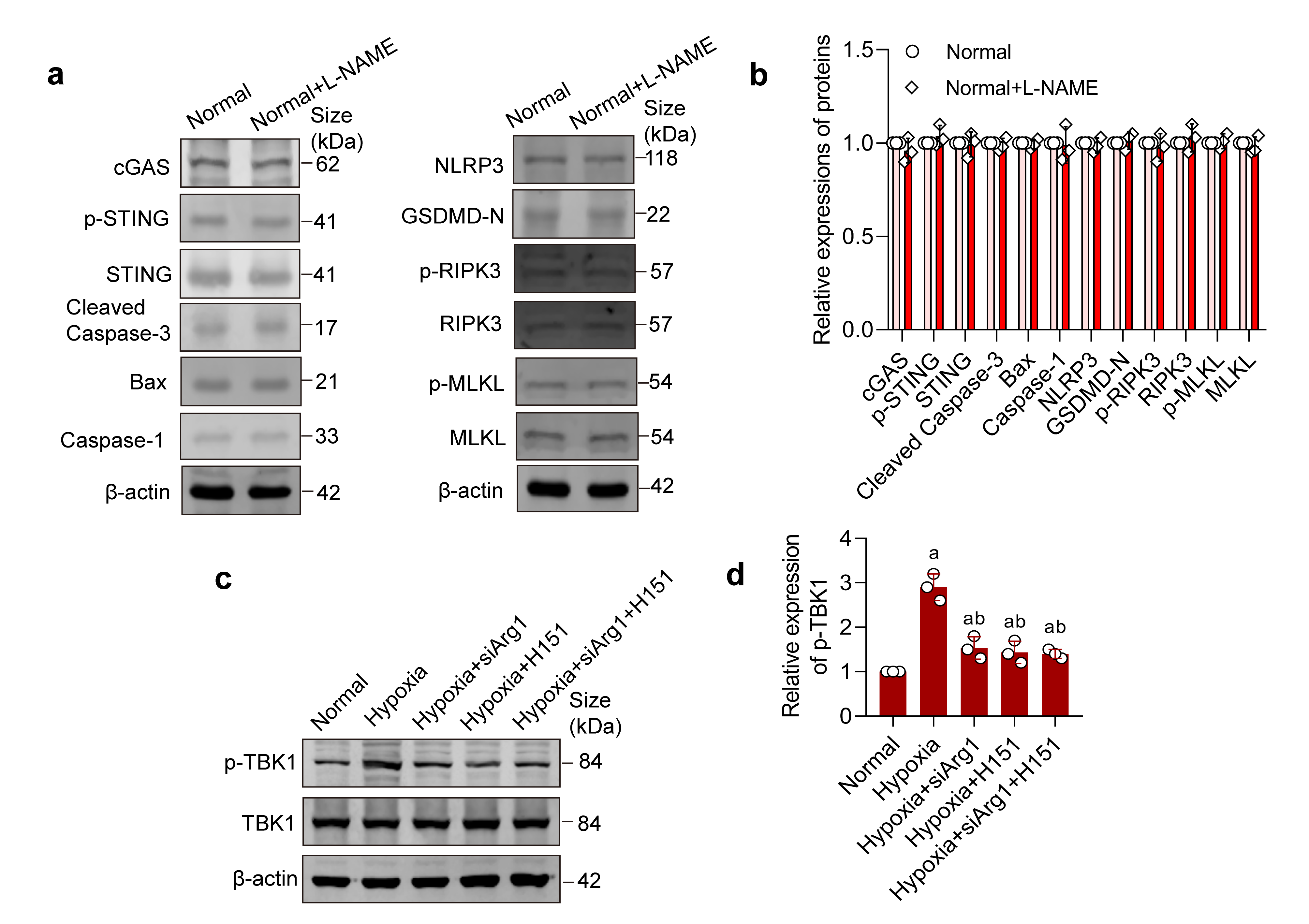


**Figure. S9.**

**a-b** Relative expression of cGAS-STING and PANoptosis-related proteins in VSMCs (n=3 independent experiments). **c-d** Relative expression of p-TBK1 in VSMCs (n=3 independent experiments). 1 μM H-151 was administered 1h prior to hypoxia treatment. a: p<0.05, as compared with the Normal group; b: p<0.05, as compared with the Hypoxia group.


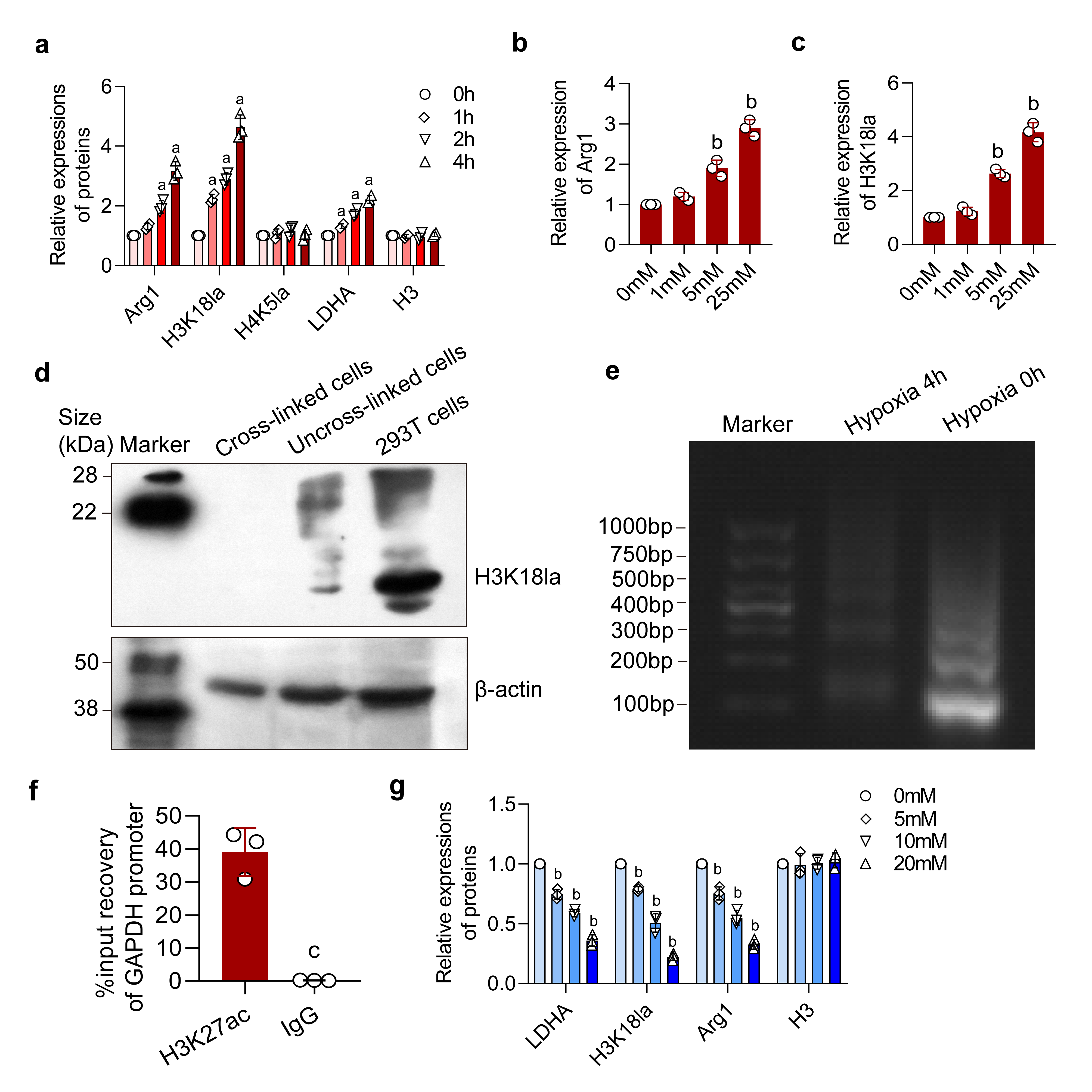


**Figure. S10.**

**a** Relative expressions of Arg1, H3K18la, H4K5la, LDHA, and Histone H3 in VSMCs. Arg1, LDHA, and Histone H3 were normalized to β-actin, while H3K18la and H4K5la were normalized to Histone H3 (n=3 independent experiments). **b** Relative expression of Arg1 in VSMCs (n=3 independent experiments). **c** Relative expressions of H3K18la in VSMCs (n=3 independent experiments). **d** WB verifies the cross-linking of cells. **e** The chromosome breakage gel image showed the DNA fragments concentrated at 100-1000bp. **f** Positive control system, the protein for positive control is H3K27ac, the gene sequence detected by qPCR is the GAPDH promoter region. Data represent three independent experiments. **g** Relative expressions of Arg1, H3K18la, LDHA and Histone H3 in VSMCs (n=3 in-dependent experiments). a: p<0.05, as compared with the 0h group; b: p<0.05, as compared with the 0 mM group; c: p<0.05, as compared with the H3K27ac group.


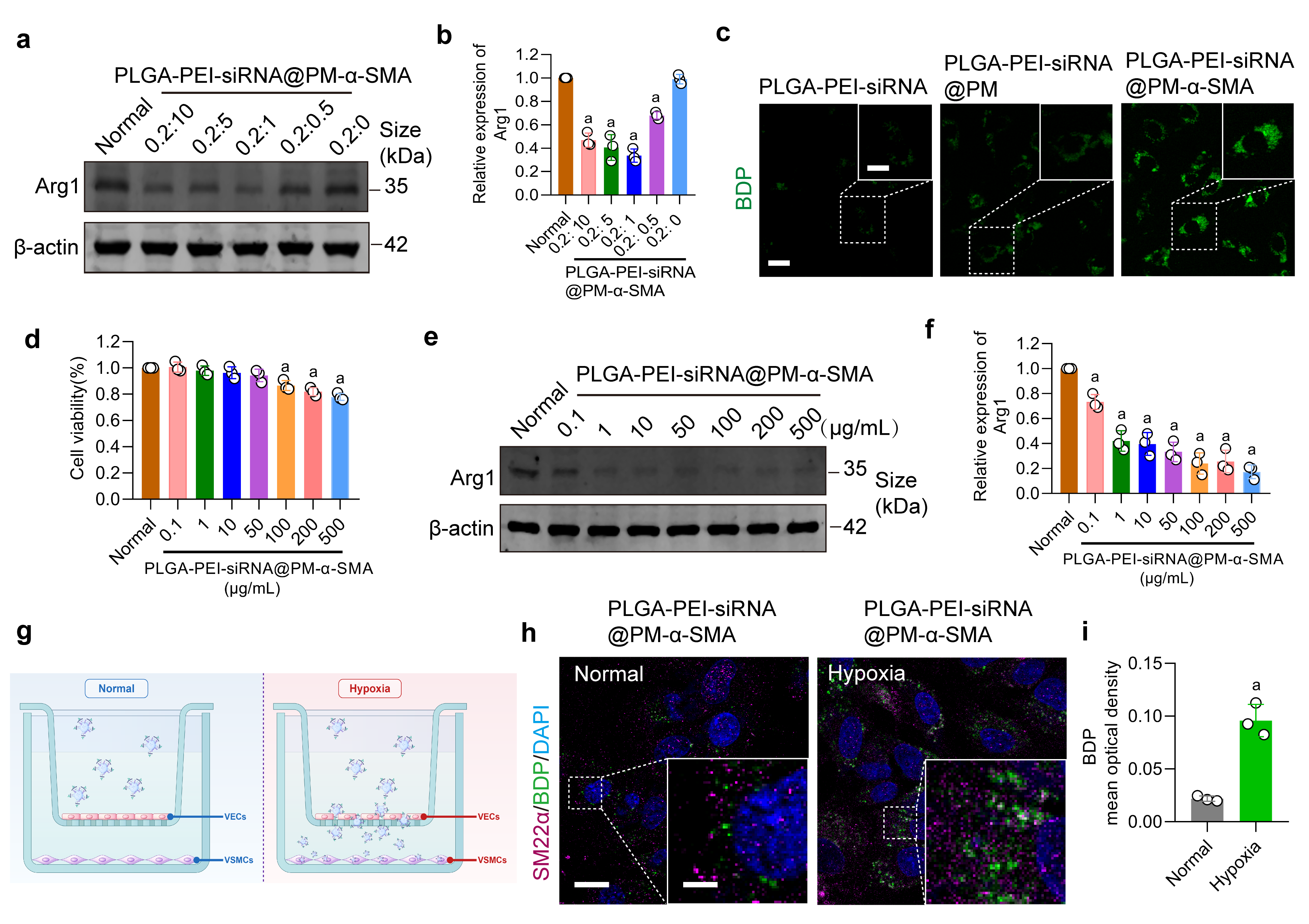


**Figure. S11.**

**a-b** Interference efficiency of PLGA-PEI-siRNA@PM-α-SMA with different PLGA:siRNA ratios (n=3 independent experiments). **c** Confocal observation of the phagocytic action of VSMCs on PLGA-PEI-siRNA, PLGA-PEI-siRNA@PM, and PLGA-PEI-siRNA@PM-α-SMA, scale bars correspond to 25 μm for low-magniﬁcation images and 10 μm for high-magniﬁcation views (n=3 independent experiments). **d** Cytotoxicity of PLGA-PEI@PM-α-SMA at different concentrations (n=3 independent experiments). **e-f** Arg1 interference efficiency of PLGA-PEI@PM-α-SMA at different concentrations (n=3 independent experiments). **g-i** Transwell assay (pore size: 0.4µm) evaluating PLGA-PEI-siRNA@PM-α-SMA penetration through VEC monolayers under hypoxia. VECs were labeled with CD31, VSMCs with SM22α, and nanoparticles with BDP, scale bars correspond to 25 μm for low-magniﬁcation images and 5 μm for high-magniﬁcation views (n=3 independent experiments). a: p<0.05, as compared with the Normal group.


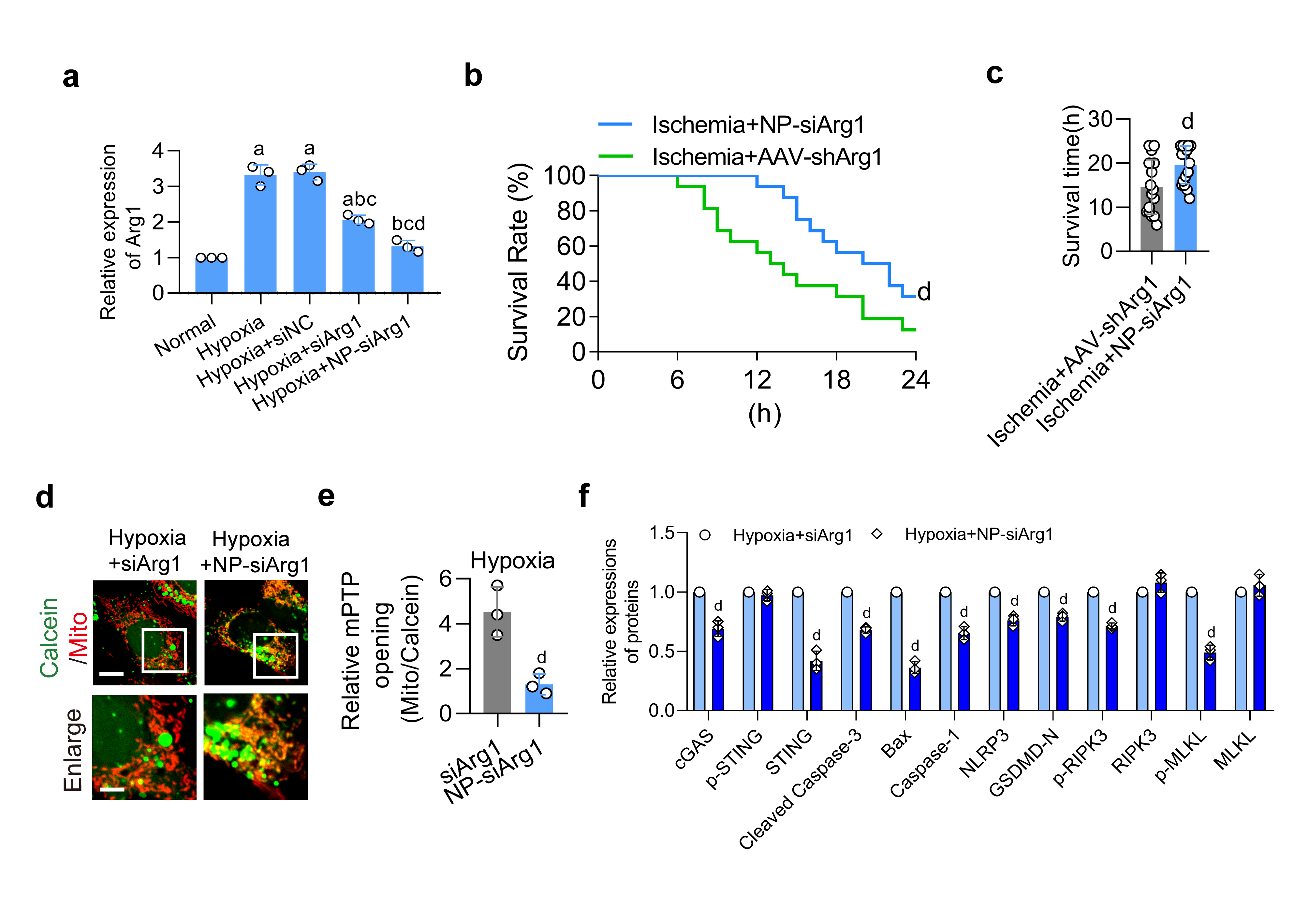


**Figure. S12.**

**a** The interference efficiency of NP-siArg1 (n=3 independent experiments). **b-c** The impact of NP-siArg1 on the 24-hour survival rate and survival time of rats experiencing ischemic injury was examined (n=16 rats in each group). **d-e** The consequences of NP-siArg1 on mPTP opening, scale bars correspond to 10 μm for low-magniﬁcation images and 5 μm for high-magniﬁcation views (n=3 independent experiments). **f** The effects of NP-siArg1 on hypoxia-induced PANoptosis in VSMCs (n=3 independent experiments). a: p<0.05, as compared with the Normal group; b: p<0.05, as compared with the Hypoxia group; c: p<0.05, as compared with the Hypoxia+siNC group; d: p<0.05, as compared with the Ischemia+AAV-shArg1 or Hypoxia+siArg1 group.

***Supplementary Tables S1-S2***

| Top 100 upregulated differential genes of VSMCs | | | | |
| --- | --- | --- | --- | --- |
| Saa3 | Mrgpra2b | Slfn4 | CT030170.4 | Ms4a4a |
| Ngp | Ifi204 | Adgre1 | Nuak2 | Oasl2 |
| Ifitm6 | Ms4a6d | Cd14 | Ggt1 | Rsad2 |
| Mmp8 | S100a8 | Map3k6 | Cdc14a | Alox5 |
| Lcn2 | Ltb4r1 | Ccl2 | Steap4 | Mefv |
| Cd177 | Padi4 | Shtn1 | Edn1 | Oas1a |
| Ccl7 | Stfa2l1 | Mmp3 | Rab20 | Gm17268 |
| Cxcl3 | Apod | F10 | Mmp25 | Batf |
| Csf3 | Sdf2l1 | Msr1 | Ly6g | Blcap |
| Fpr1 | Prok2 | Ly6c1 | Plscr1 | Mt2 |
| Ccl12 | Wfdc21 | Tmem252 | Nfe2 | Clec4a2 |
| Ch25h | Rgs17 | Osgin2 | Bst1 | Saa1 |
| Ltf | Zkscan14 | Jpt2 | Gm43814 | Slc7a11 |
| Gm5483 | Ly6c2 | Usp18 | Scara5 | Nos2 |
| Lrg1 | S100a9 | Gemin6 | Gpr141 | Slc2a6 |
| Il6 | Heatr1 | Arid3a | Wfdc17 | Dgat2 |
| Pglyrp1 | Arg1 | Lif | Ikbke | Ccl9 |
| AA467197 | Itgb2l | C1qb | Ccdc162 | Ass1 |
| Camp | Fosl1 | Orm1 | Il1rn | Basp1 |
| I830127L07Rik | Gm13293 | Csf2rb2 | Oasl1 | Mt1 |

**Table. S1. Top 100 upregulated differential genes of VSMCs from single-cell sequencing.**

| Gene name | Forward primer | Reverse primer |
| --- | --- | --- |
| b-globin | 5′-GAACCCTGATGATGTTGGTGG-3′ | 5′-GGTTGTCCAAGTGTTTCAGGC-3′ |
| mtCOI | 5′-TGGCTTACAAGACGCCACAT-3′ | 5′-TGGGCGTCTATTGTGCTTGT-3′ |
| GAPDH | 5′-GAAGCTGGTCATCAACGGGA-3′ | 5′-CGACATACTCAGCACCAGCA-3′ |

**Table. S2. Primer sequence of nDNA, mtDNA and GAPDH.**
