# Supplementary material for: Arginase 1 drives mitochondrial cristae remodeling and PANoptosis in ischemia/hypoxia-induced vascular dysfunction
Source: Signal Transduct Target Ther. 2025 May 28;10:167. doi: 10.1038/s41392-025-02255-2 (PMC12117058; doi:10.1038/s41392-025-02255-2)

Figure2j

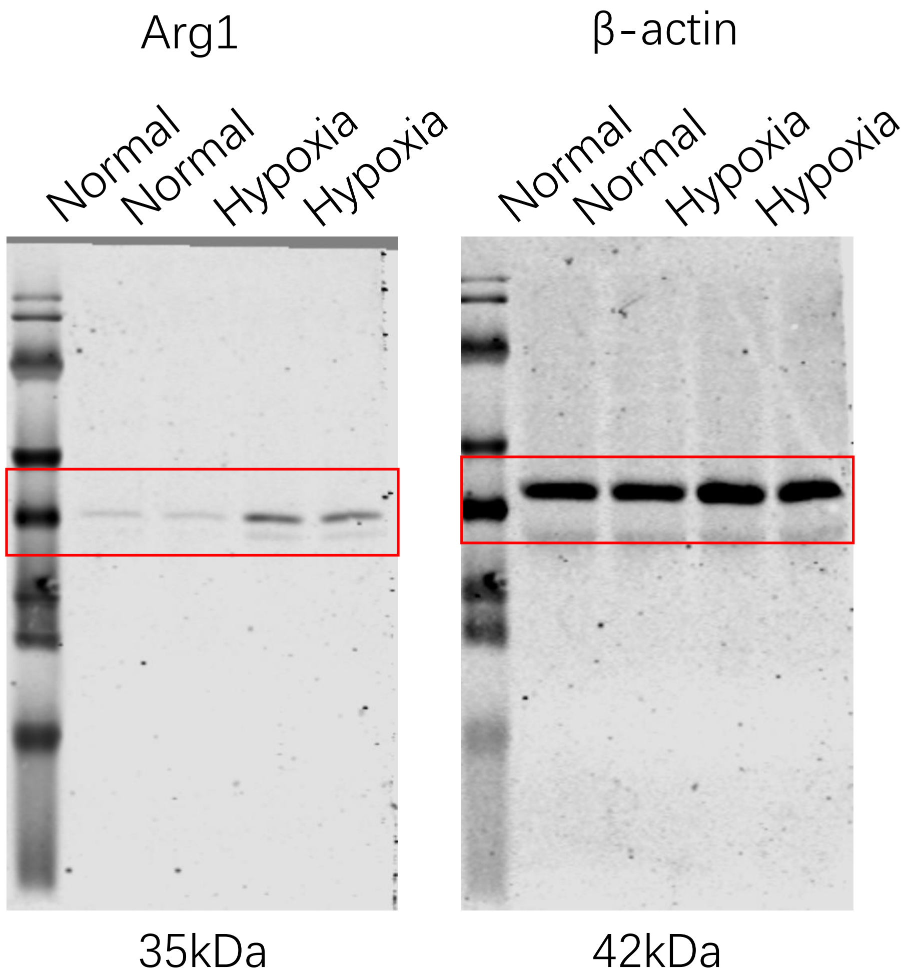
Arg1
β-actin
Normal
Normal
Hypoxia
Hypoxia
Normal
Normal
Hypoxia
Hypoxia
35kDa
42kDa

Figure2k

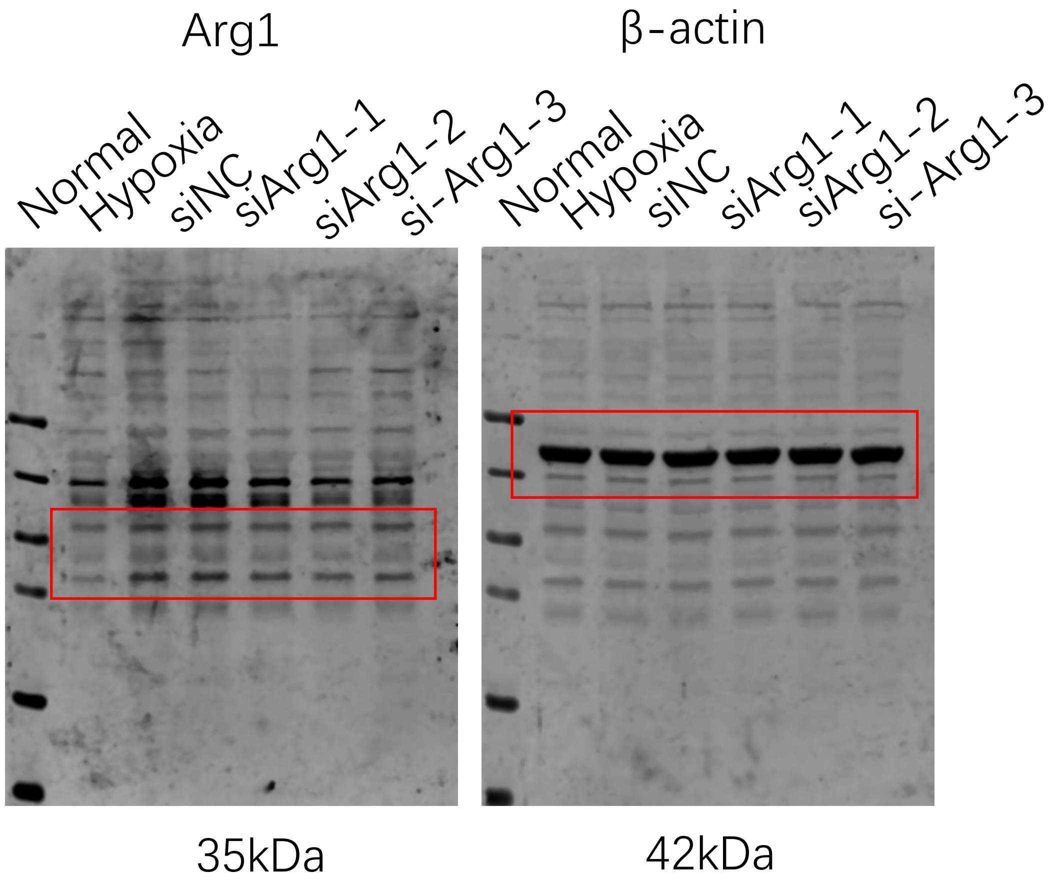
Arg1
β-actin
Normal
Hypoxia
siNC
siArg1-1
siArg1-2
si-Arg1-3
Normal
Hypoxia
siNC
siArg1-1
siArg1-2
si-Arg1-3
35kDa
42kDa

Figure3e

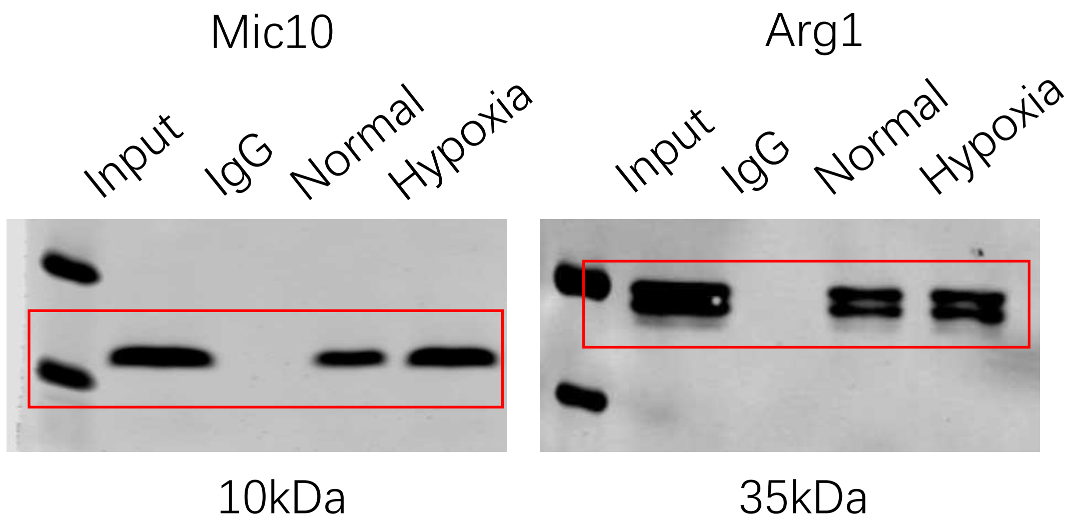
Mic10
Input
IgG
Normal
Hypoxia
10kDa
Arg1
Input
IgG
Normal
Hypoxia
35kDa

Figure3f

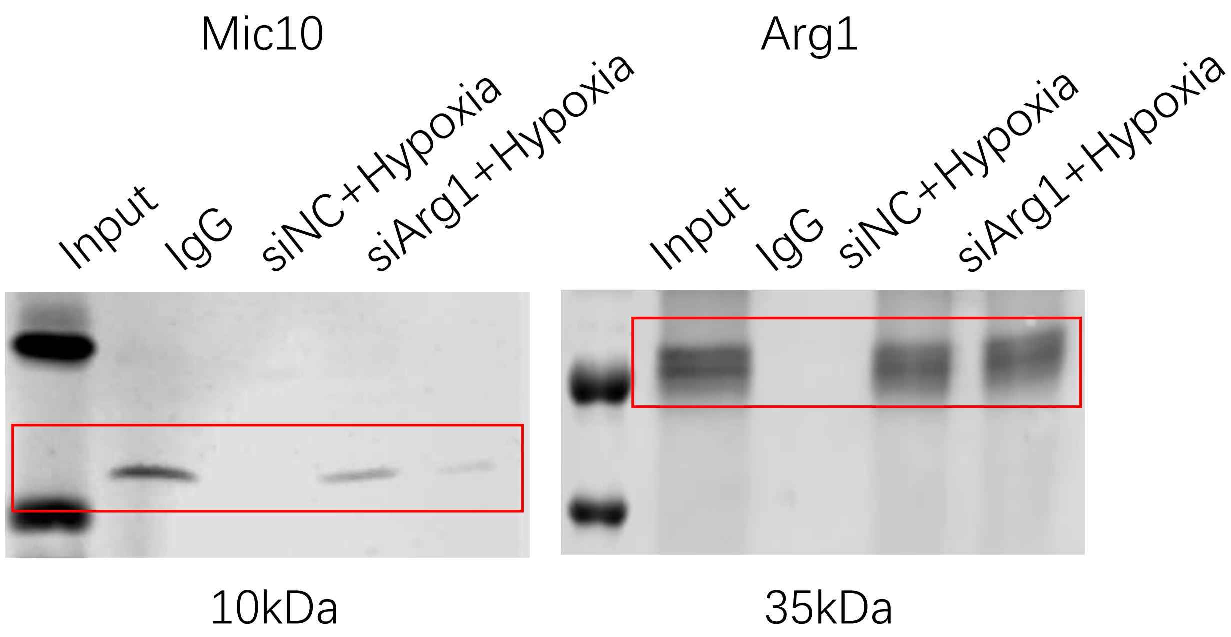
Mic10
Input
IgG
siNC+Hypoxia
siArg1+Hypoxia
10kDa
Arg1
Input
IgG
siNC+Hypoxia
siArg1+Hypoxia
35kDa

Figure3h

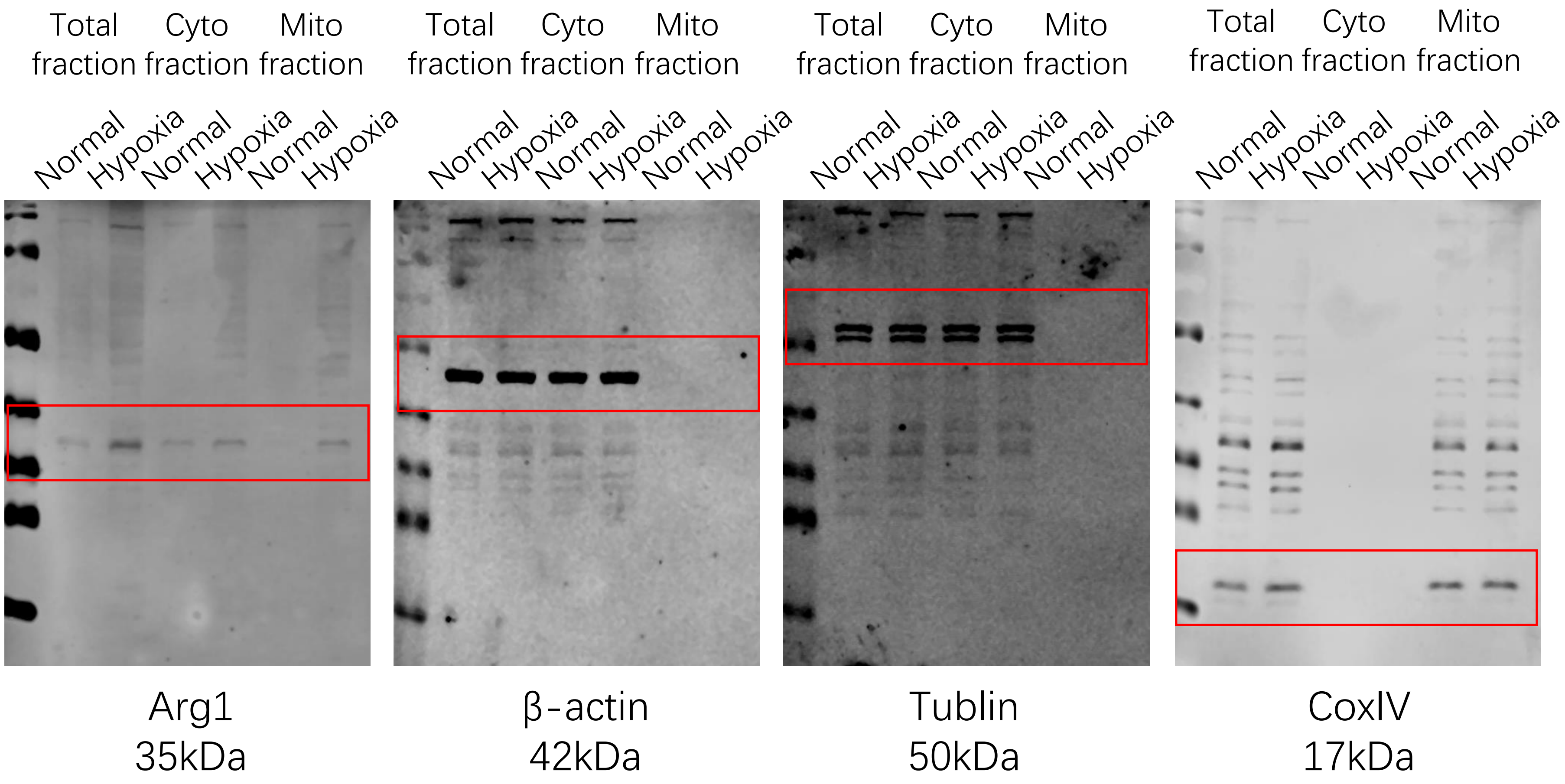
Total fraction
Cyto fraction
Mito fraction
Normal
Hypoxia
Normal
Hypoxia
Normal
Hypoxia
Arg1
35kDa
β-actin
42kDa
Tublin
50kDa
CoxIV
17kDa

Figure3j

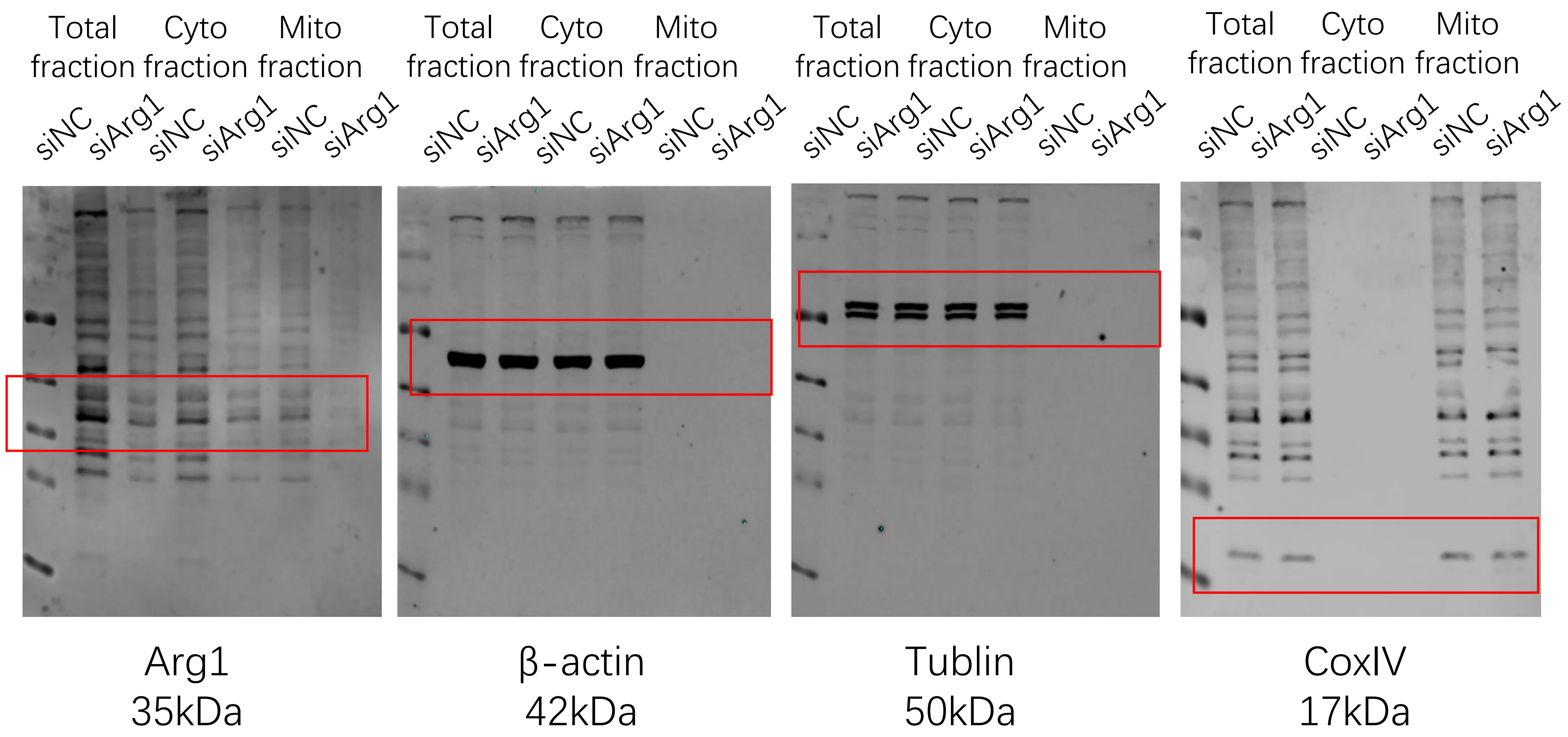
Total fraction
Cyto fraction
Mito fraction
siNC
siArg1
siNC
siArg1
siNC
siArg1
Arg1
35kDa
β-actin
42kDa
Tublin
50kDa
CoxIV
17kDa

Figure4a

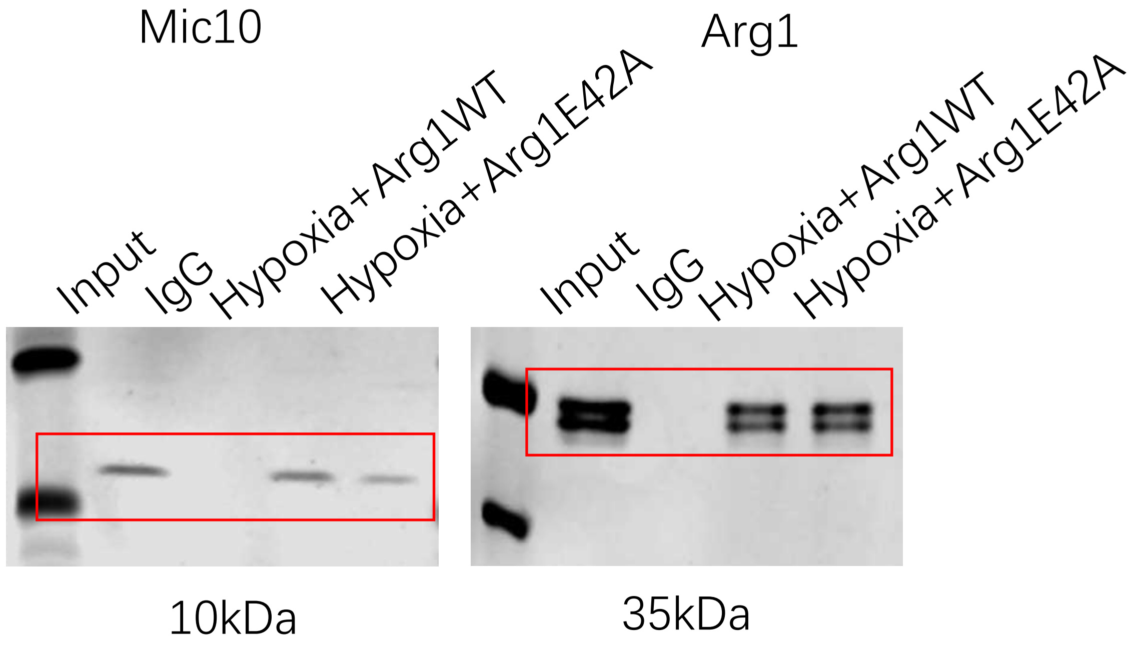
Mic10
Input
IgG
Hypoxia+Arg1WT
Hypoxia+Arg1E42A
10kDa
Arg1
Input
IgG
Hypoxia+Arg1WT
Hypoxia+Arg1E42A
35kDa

Figure5d

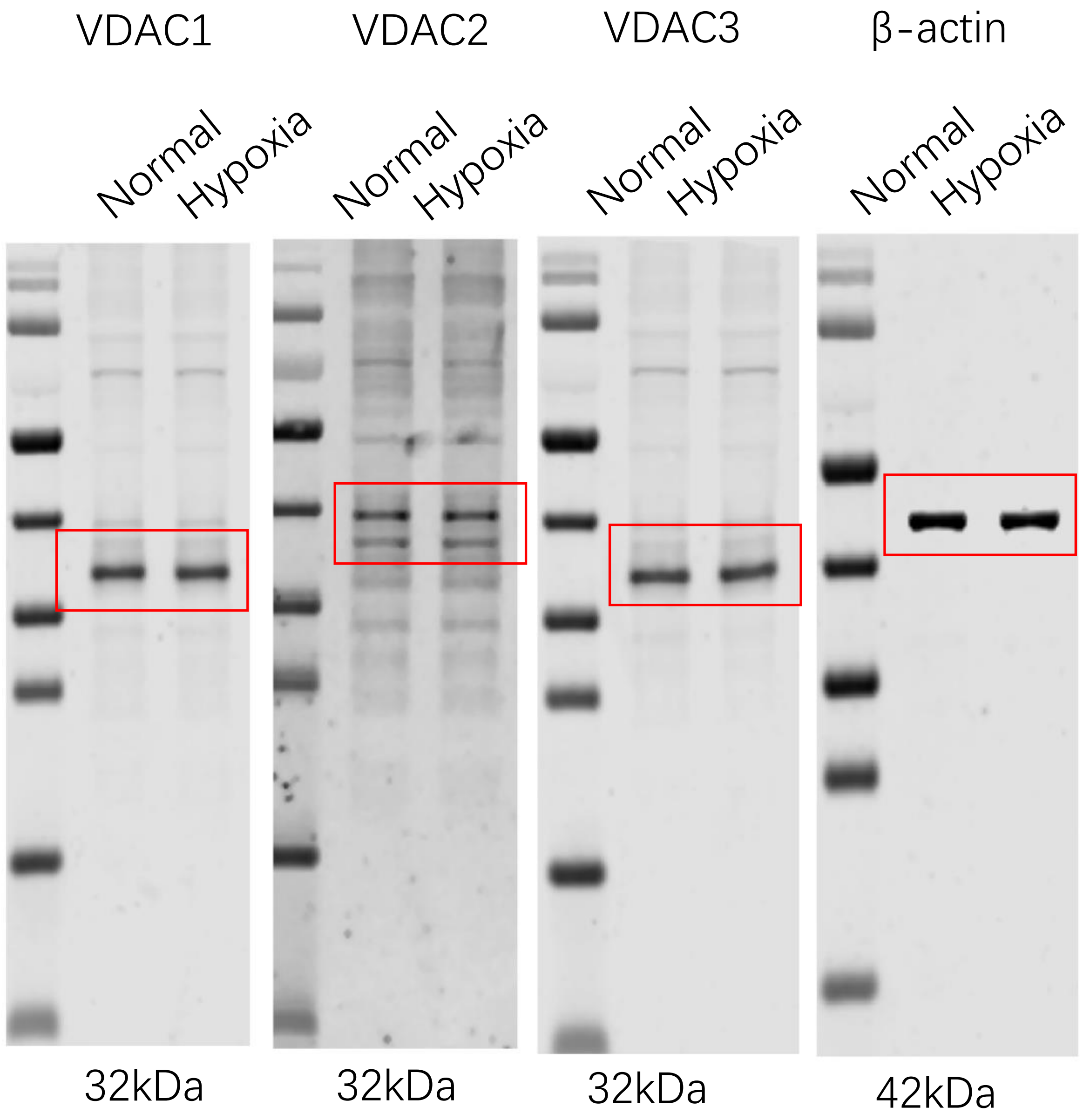
VDAC1
VDAC2
VDAC3
β-actin
Normal
Hypoxia
Normal
Hypoxia
Normal
Hypoxia
Normal
Hypoxia
32kDa
32kDa
32kDa
42kDa

Figure5e

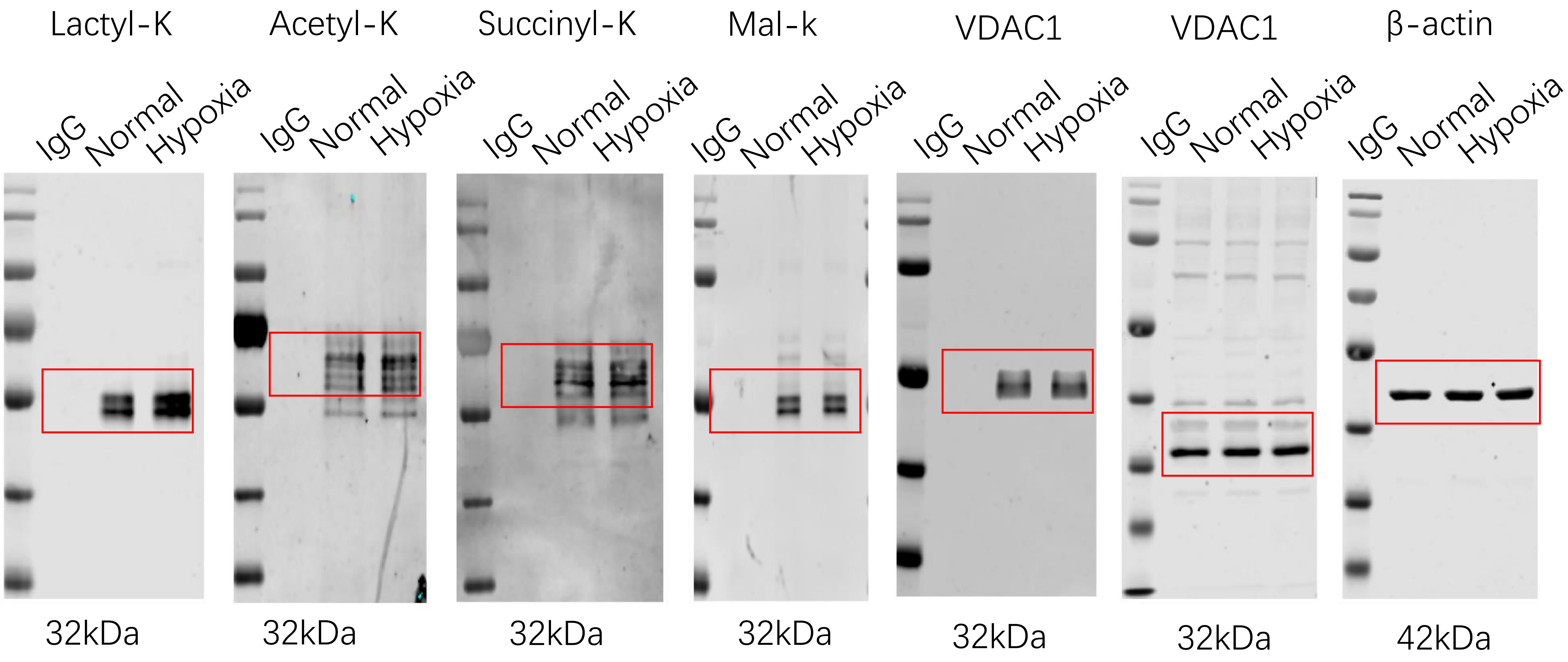

Lactyl-K
Acetyl-K
Succinyl-K
Mal-k
VDAC1
VDAC1
β-actin
IgG
Normal
Hypoxia
32kDa
32kDa
32kDa
32kDa
32kDa
32kDa
42kDa

Figure5f

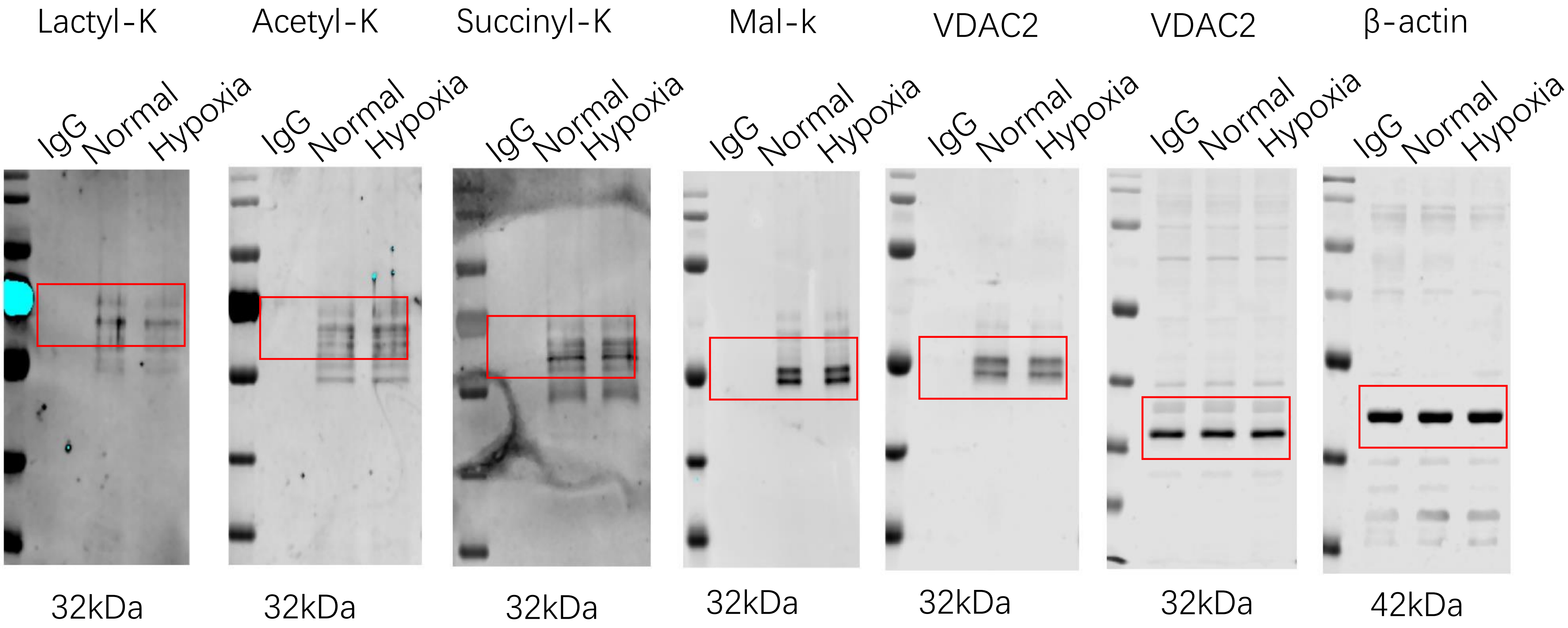
Lactyl-K
Acetyl-K
Succinyl-K
Mal-k
VDAC2
VDAC2
β-actin
IgG
Normal
Hypoxia
32kDa
32kDa
32kDa
32kDa
32kDa
32kDa
42kDa

Figure5g

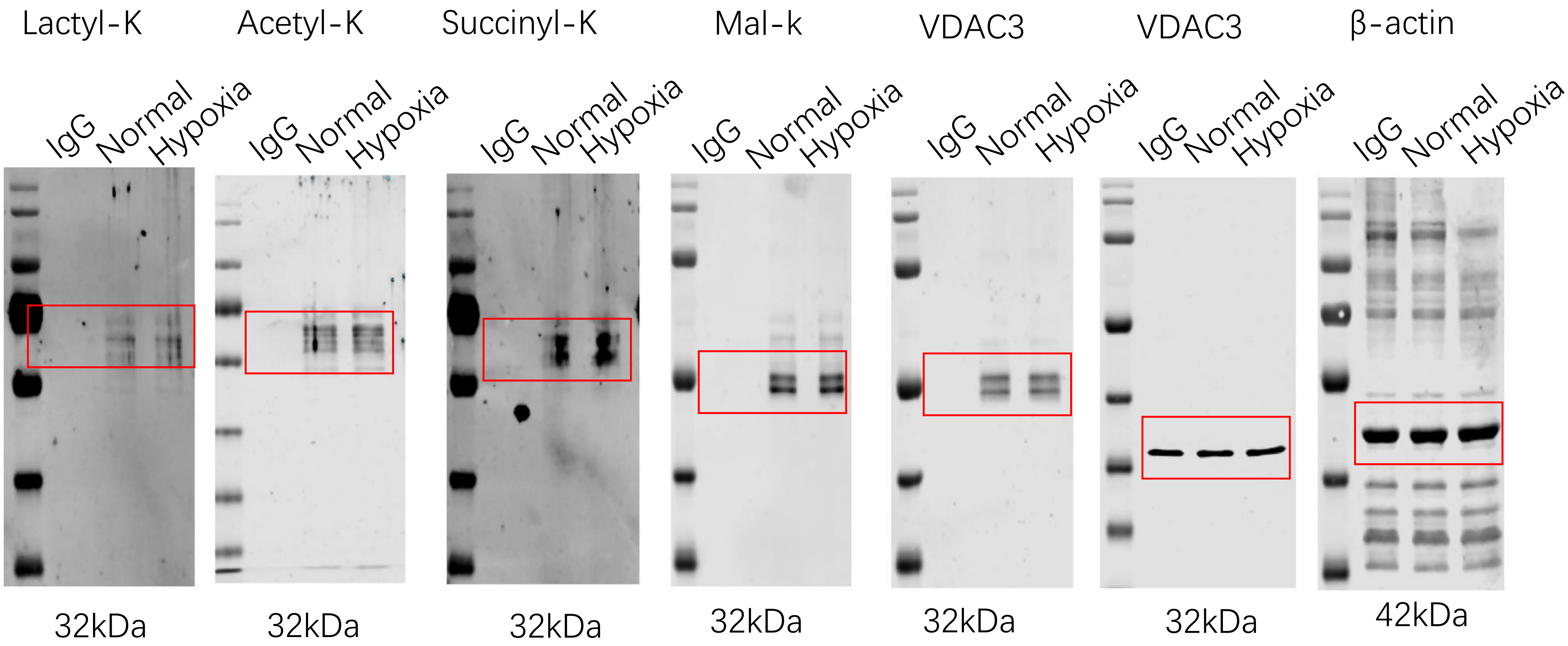
Lactyl-K
Acetyl-K
Succinyl-K
Mal-k
VDAC3
VDAC3
β-actin
IgG
Normal
Hypoxia
32kDa
32kDa
32kDa
32kDa
32kDa
32kDa
42kDa

Figure5h

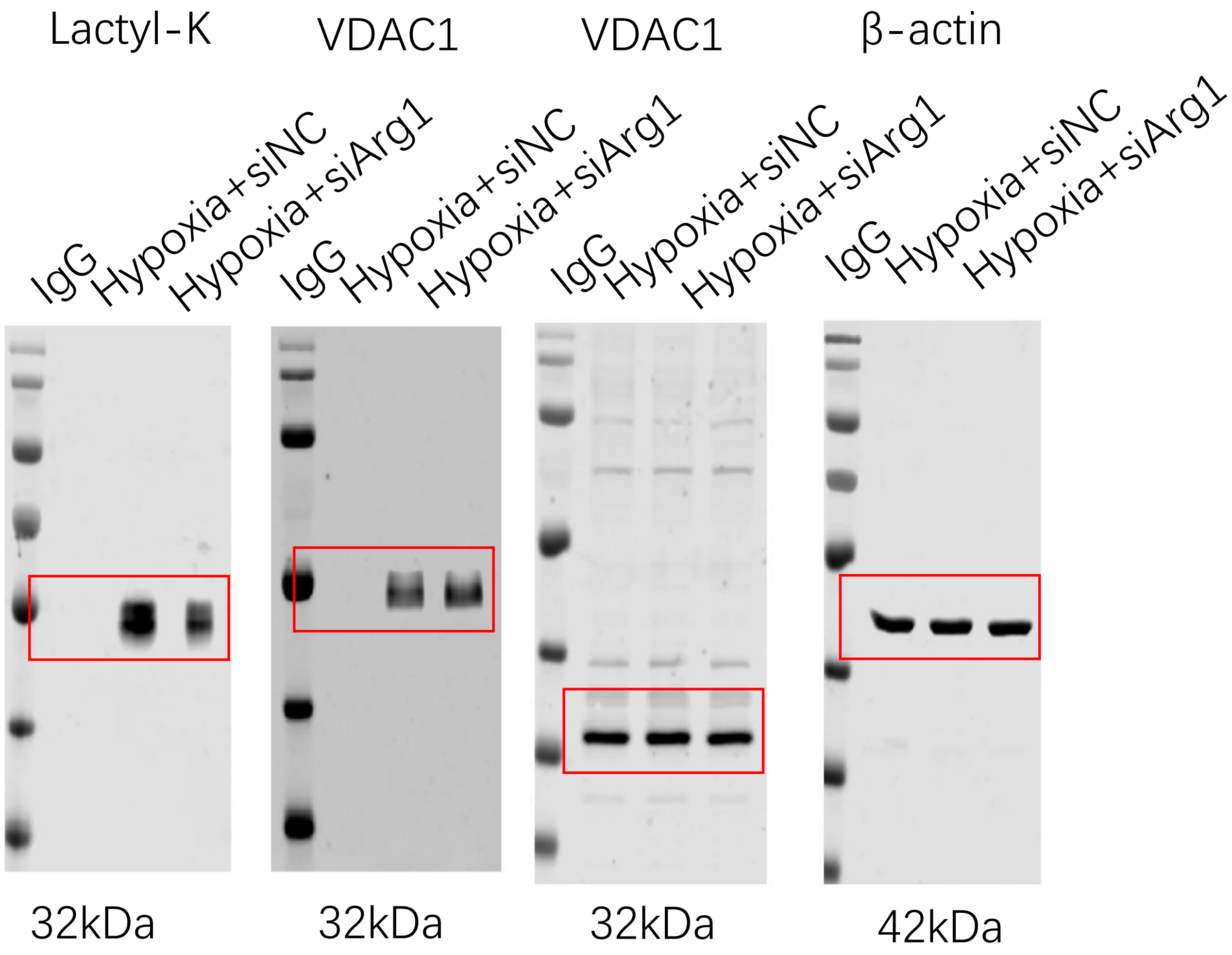
Lactyl-K
VDAC1
VDAC1
β-actin
IgG
Hypoxia+siNC
Hypoxia+siArg1
IgG
Hypoxia+siNC
Hypoxia+siArg1
IgG
Hypoxia+siNC
Hypoxia+siArg1
IgG
Hypoxia+siNC
Hypoxia+siArg1
32kDa
32kDa
32kDa
42kDa

Figure6a

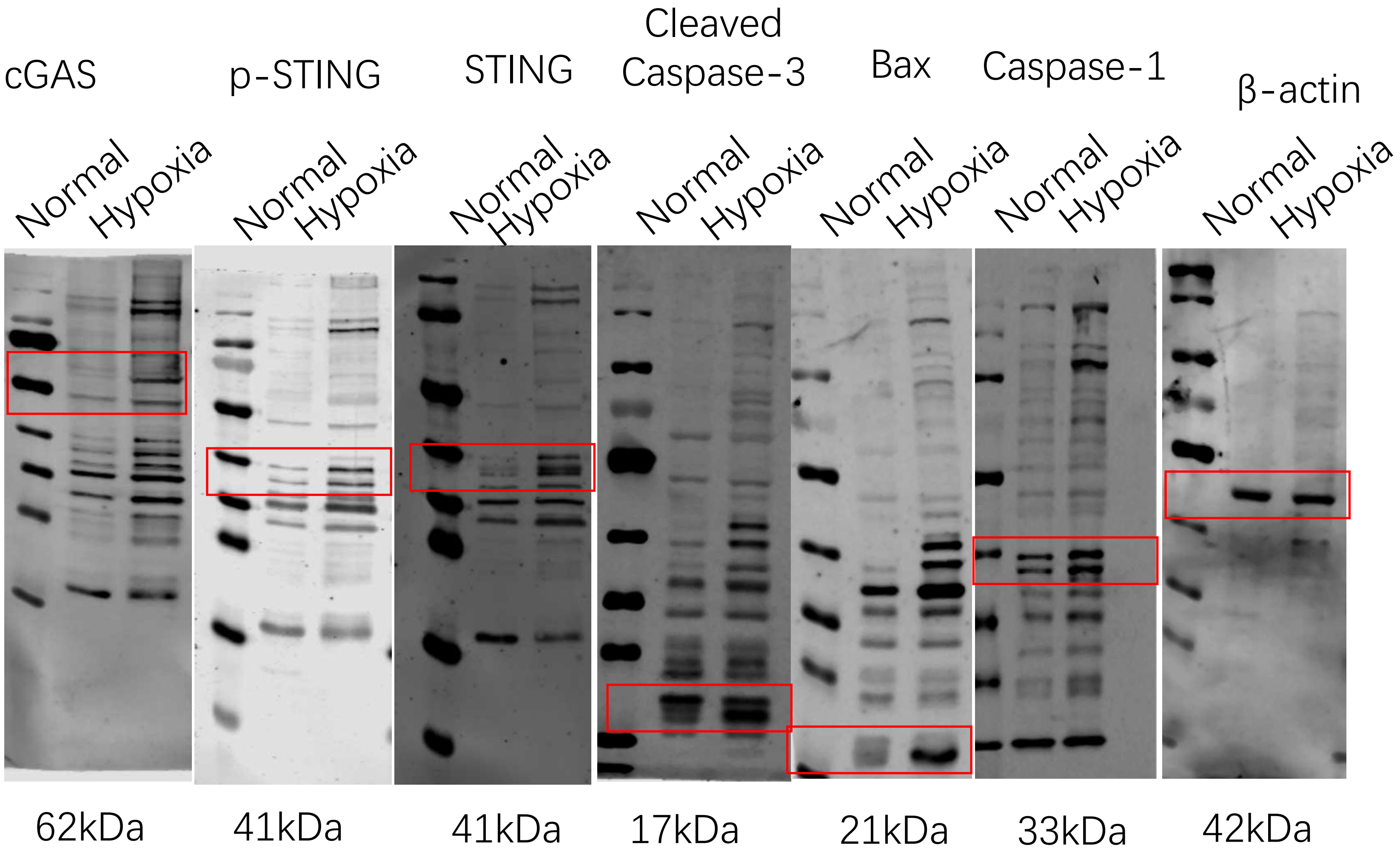
cGAS
p-STING
STING
Cleaved Caspase-3
Bax
Caspase-1
β-actin
Normal
Hypoxia
Normal
Hypoxia
Normal
Hypoxia
Normal
Hypoxia
Normal
Hypoxia
Normal
Hypoxia
Normal
Hypoxia
62kDa
41kDa
41kDa
17kDa
21kDa
33kDa
42kDa

Figure6b

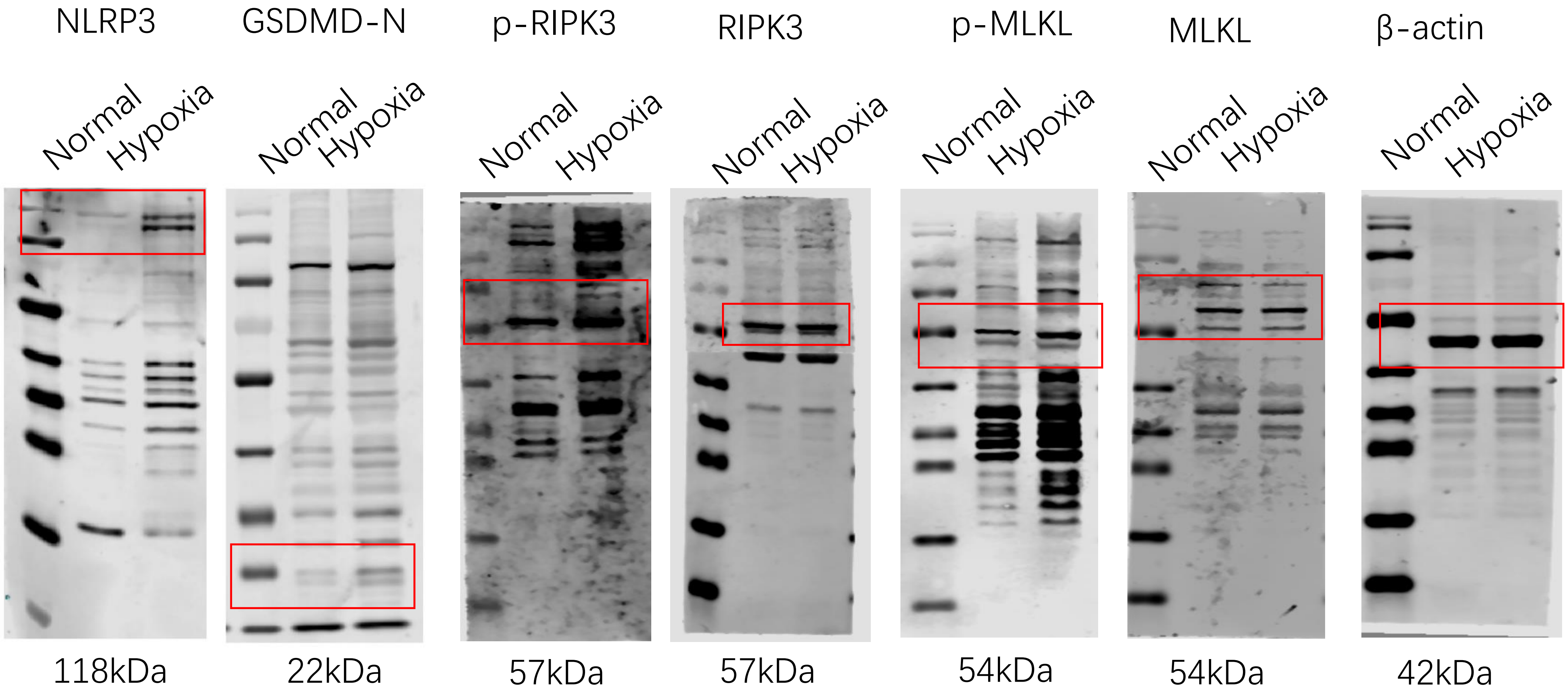
NLRP3
GSDMD-N
p-RIPK3
RIPK3
p-MLKL
MLKL
β-actin
Normal
Hypoxia
118kDa
22kDa
57kDa
57kDa
54kDa
54kDa
42kDa

Figure6c

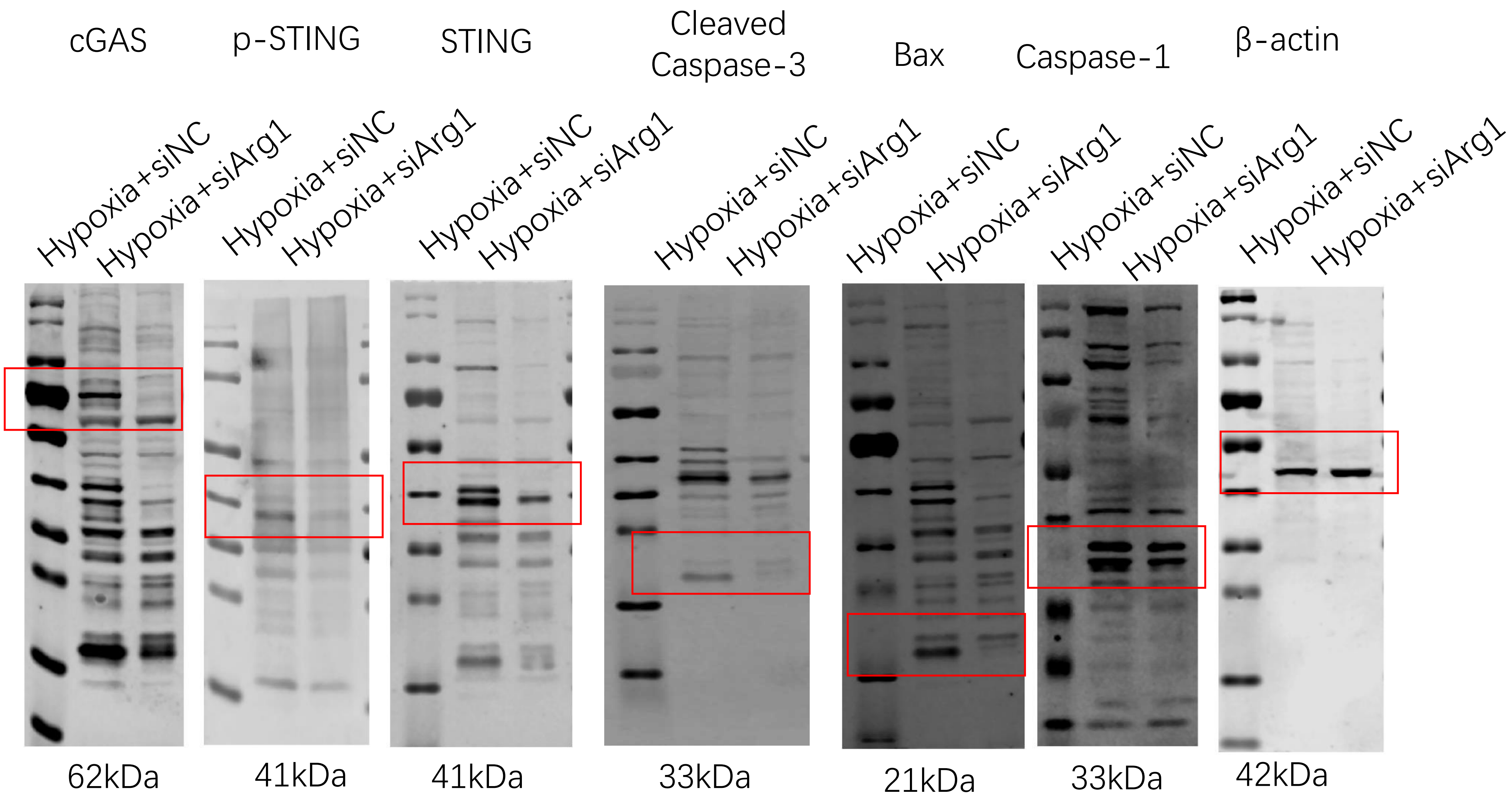
cGAS
p-STING
STING
Cleaved Caspase-3
Bax
Caspase-1
β-actin
Hypoxia+siNC
Hypoxia+siArg1
Hypoxia+siNC
Hypoxia+siArg1
Hypoxia+siNC
Hypoxia+siArg1
Hypoxia+siNC
Hypoxia+siArg1
Hypoxia+siNC
Hypoxia+siArg1
Hypoxia+siNC
Hypoxia+siArg1
Hypoxia+siNC
Hypoxia+siArg1
62kDa
41kDa
41kDa
33kDa
21kDa
33kDa
42kDa

Figure6d

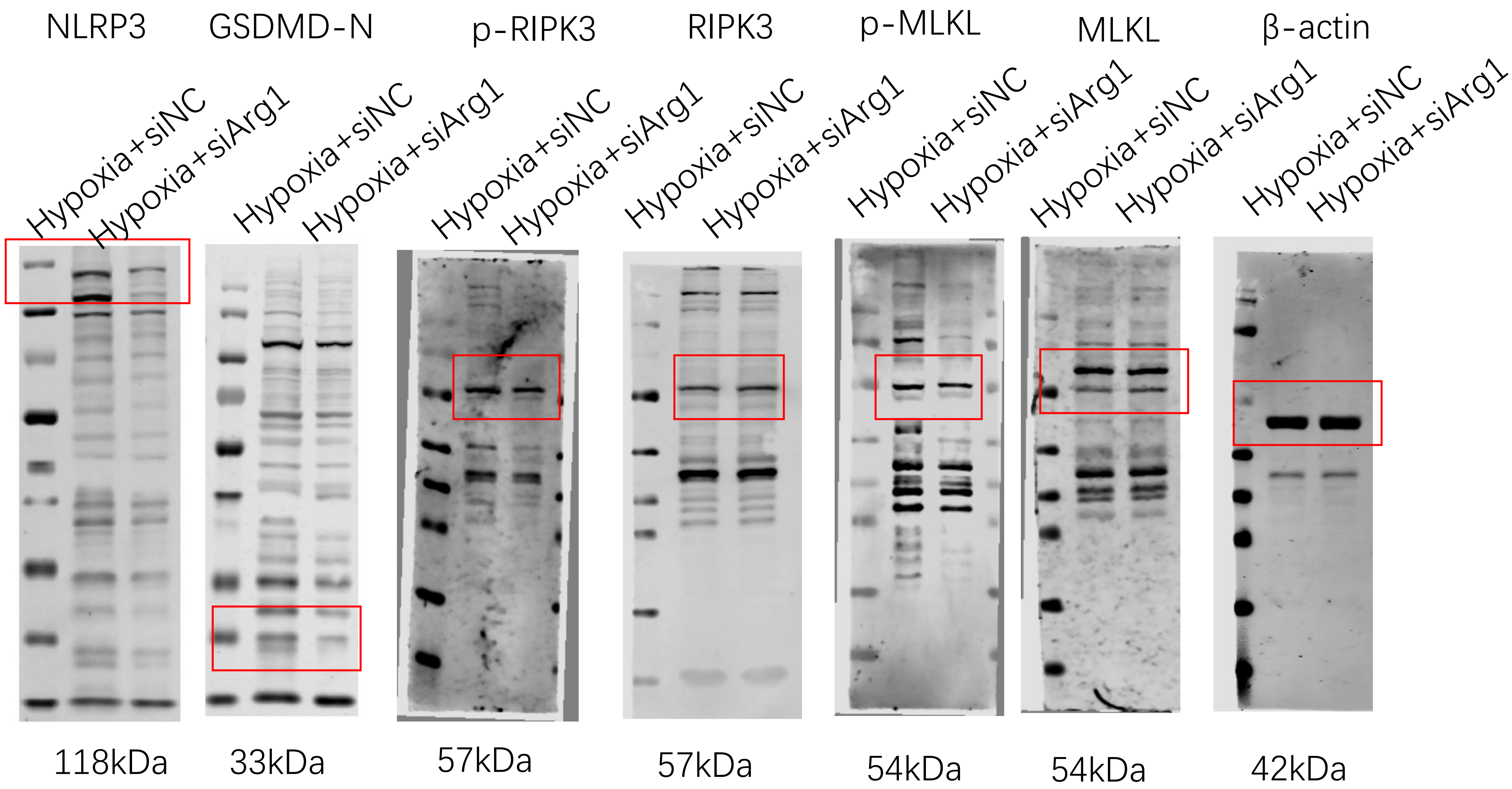
NLRP3
GSDMD-N
p-RIPK3
RIPK3
p-MLKL
MLKL
β-actin
Hypoxia+siNC
Hypoxia+siArg1
Hypoxia+siNC
Hypoxia+siArg1
Hypoxia+siNC
Hypoxia+siArg1
Hypoxia+siNC
Hypoxia+siArg1
Hypoxia+siNC
Hypoxia+siArg1
Hypoxia+siNC
Hypoxia+siArg1
Hypoxia+siNC
Hypoxia+siArg1
118kDa
33kDa
57kDa
57kDa
54kDa
54kDa
42kDa

Figure6e

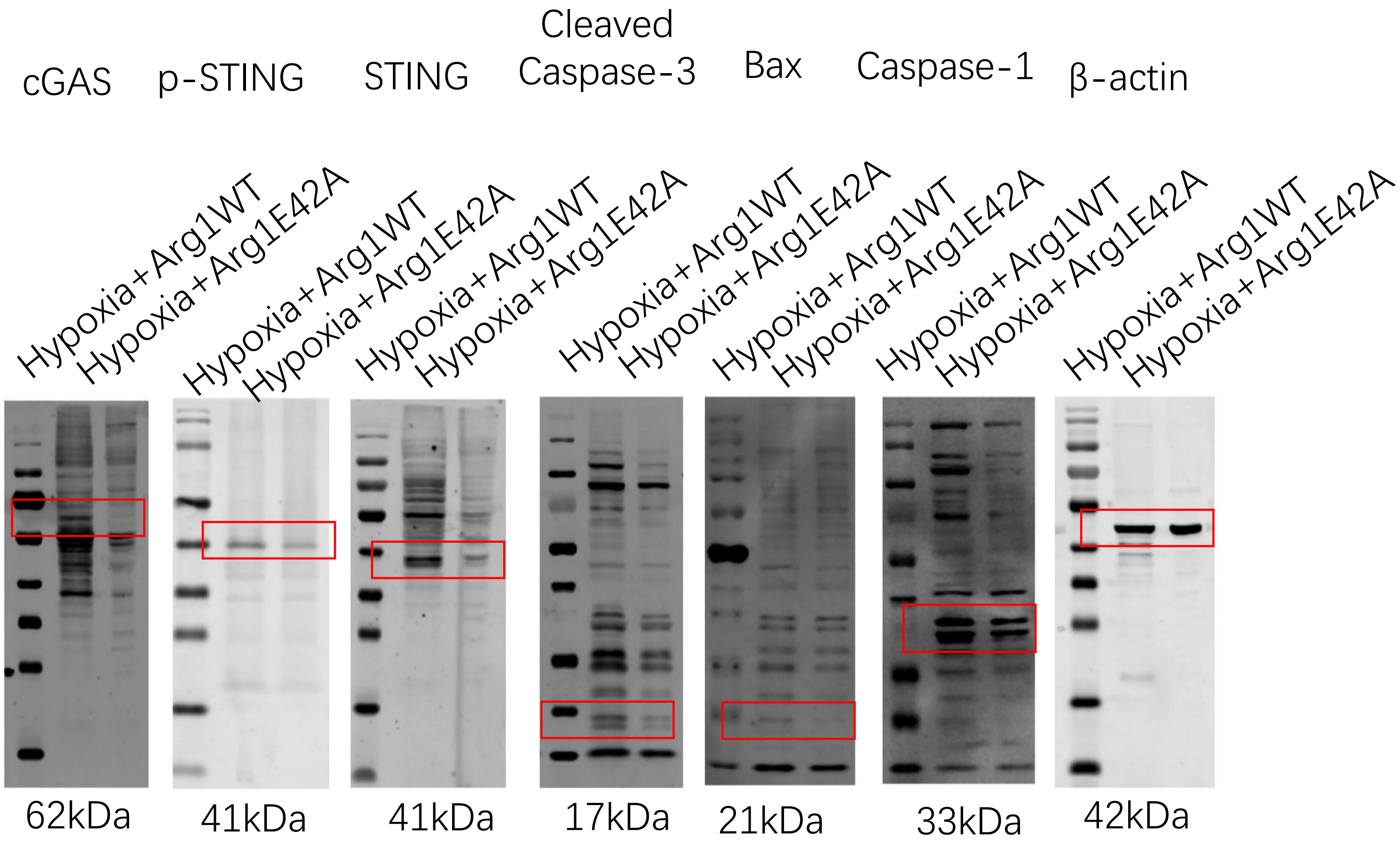
cGAS
p-STING
STING
Cleaved Caspase-3
Bax
Caspase-1
β-actin
Hypoxia+Arg1WT
Hypoxia+Arg1E42A
Hypoxia+Arg1WT
Hypoxia+Arg1E42A
Hypoxia+Arg1WT
Hypoxia+Arg1E42A
Hypoxia+Arg1WT
Hypoxia+Arg1E42A
Hypoxia+Arg1WT
Hypoxia+Arg1E42A
Hypoxia+Arg1WT
Hypoxia+Arg1E42A
Hypoxia+Arg1WT
Hypoxia+Arg1E42A
62kDa
41kDa
41kDa
17kDa
21kDa
33kDa
42kDa

Figure6f

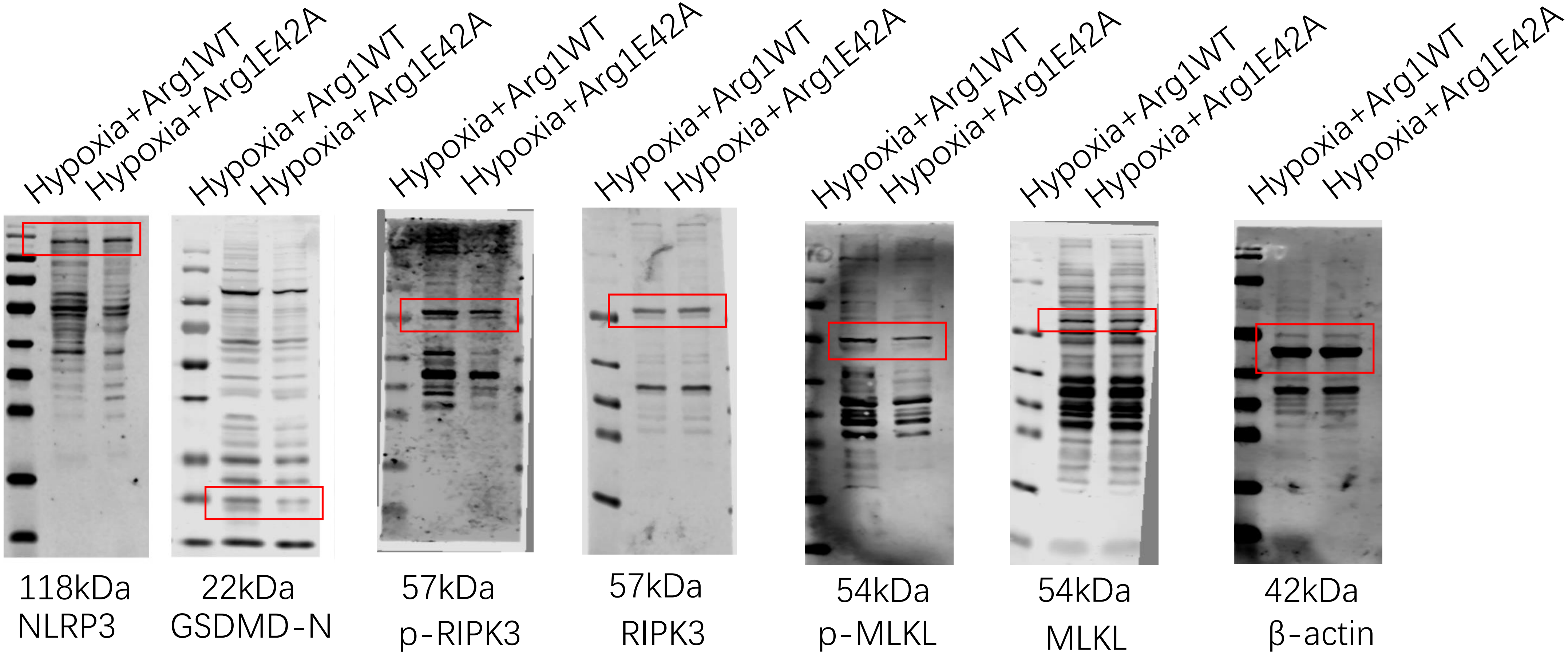
Hypoxia+Arg1WT
Hypoxia+Arg1E42A
Hypoxia+Arg1WT
Hypoxia+Arg1E42A
Hypoxia+Arg1WT
Hypoxia+Arg1E42A
Hypoxia+Arg1WT
Hypoxia+Arg1E42A
Hypoxia+Arg1WT
Hypoxia+Arg1E42A
Hypoxia+Arg1WT
Hypoxia+Arg1E42A
Hypoxia+Arg1WT
Hypoxia+Arg1E42A
118kDa
NLRP3
22kDa
GSDMD-N
57kDa
p-RIPK3
57kDa
RIPK3
54kDa
p-MLKL
54kDa
MLKL
42kDa
β-actin

Figure6i

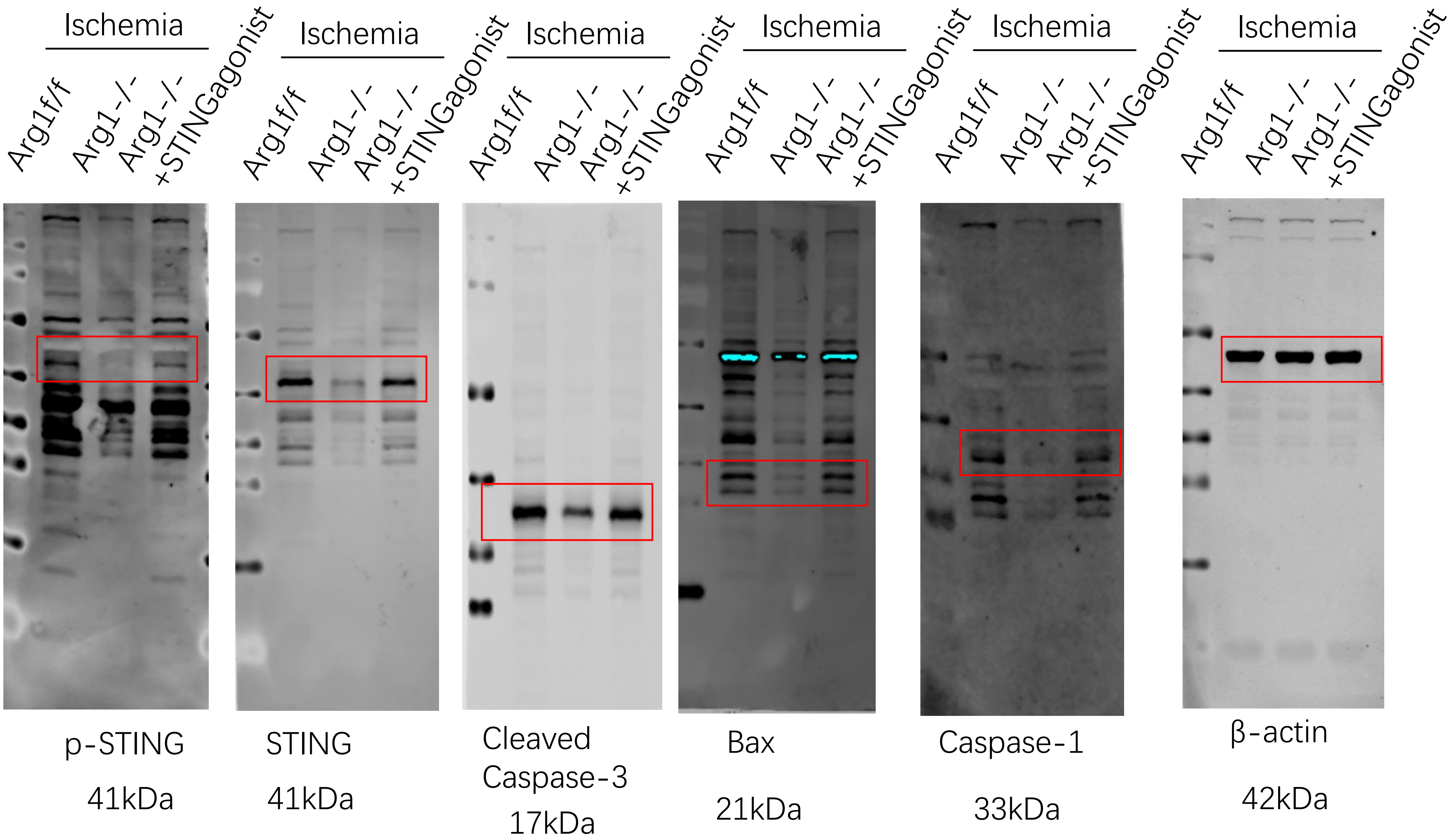

Ischemia
Arg1f/f
Arg1-/-
Arg1-/-
+STINGagonist
p-STING
41kDa
STING
41kDa
Cleaved
Caspase-3
17kDa
Bax
21kDa
Caspase-1
33kDa
β-actin
42kDa

Figure6j

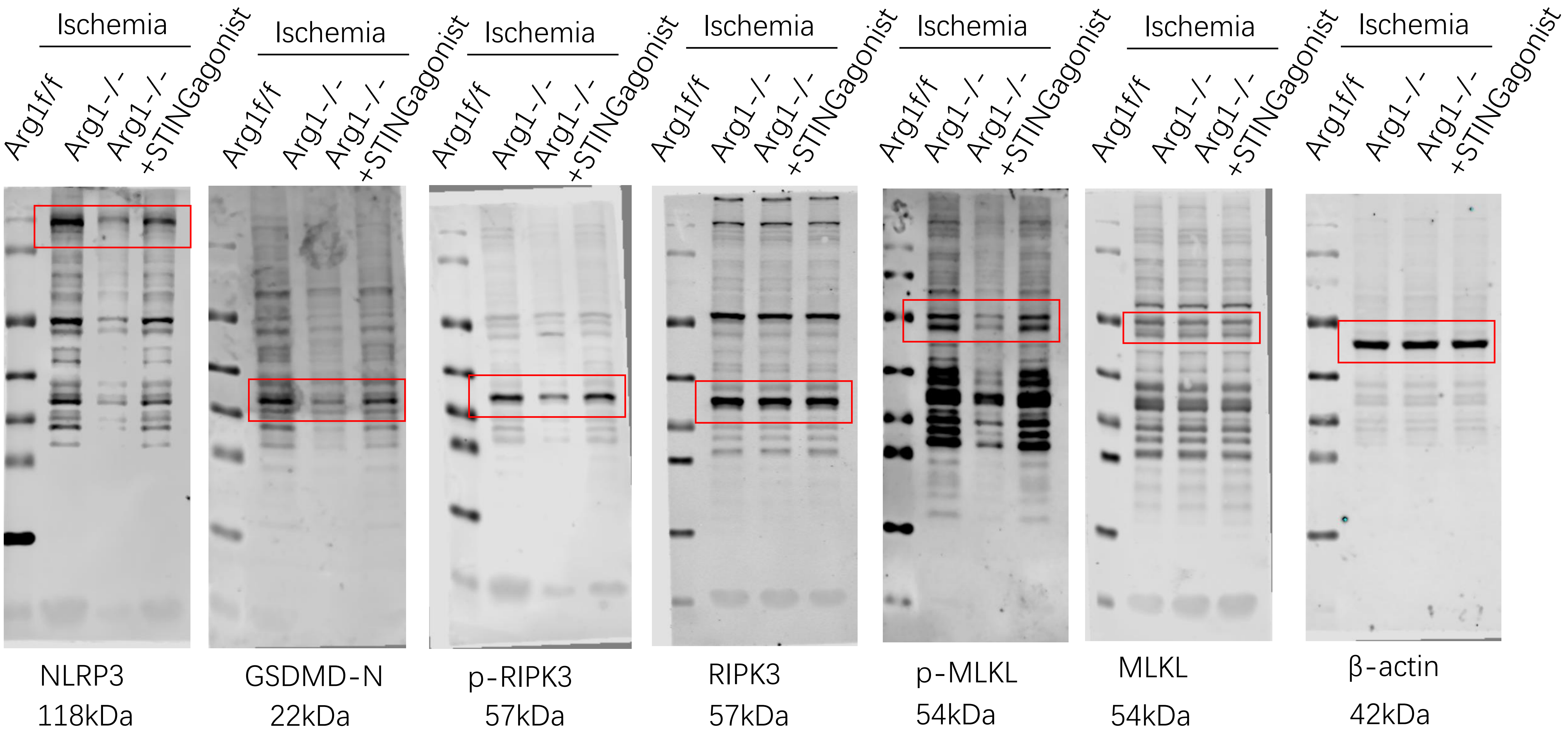
Ischemia
Arg1f/f
Arg1-/-
Arg1-/-
+STINGagonist
NLRP3
118kDa
GSDMD-N
22kDa
p-RIPK3
57kDa
RIPK3
57kDa
p-MLKL
54kDa
MLKL
54kDa
β-actin
42kDa

Figure7b

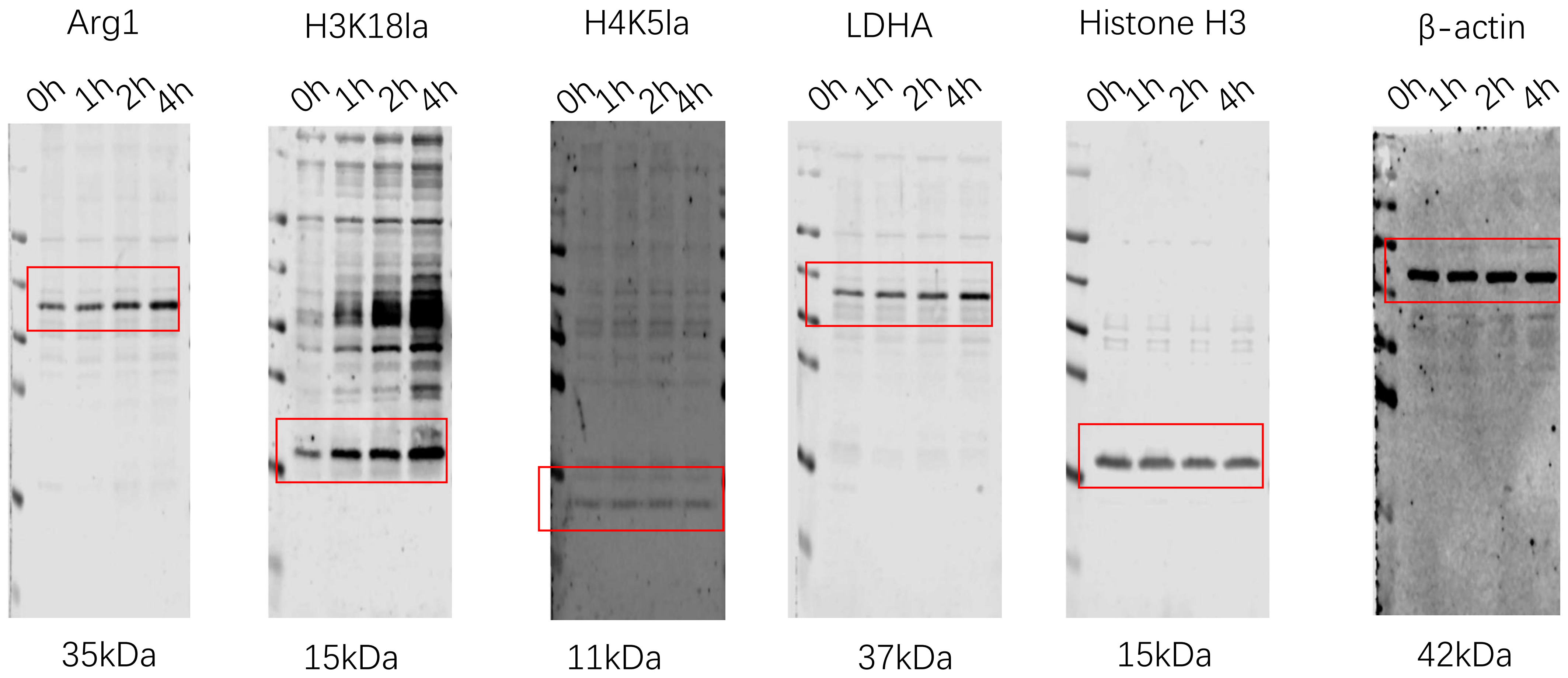
Arg1
H3K18la
H4K5la
LDHA
Histone H3
β-actin
0h 1h 2h 4h
0h 1h 2h 4h
0h 1h 2h 4h
0h 1h 2h 4h
0h 1h 2h 4h
0h 1h 2h 4h
35kDa
15kDa
11kDa
37kDa
15kDa
42kDa

Figure7d

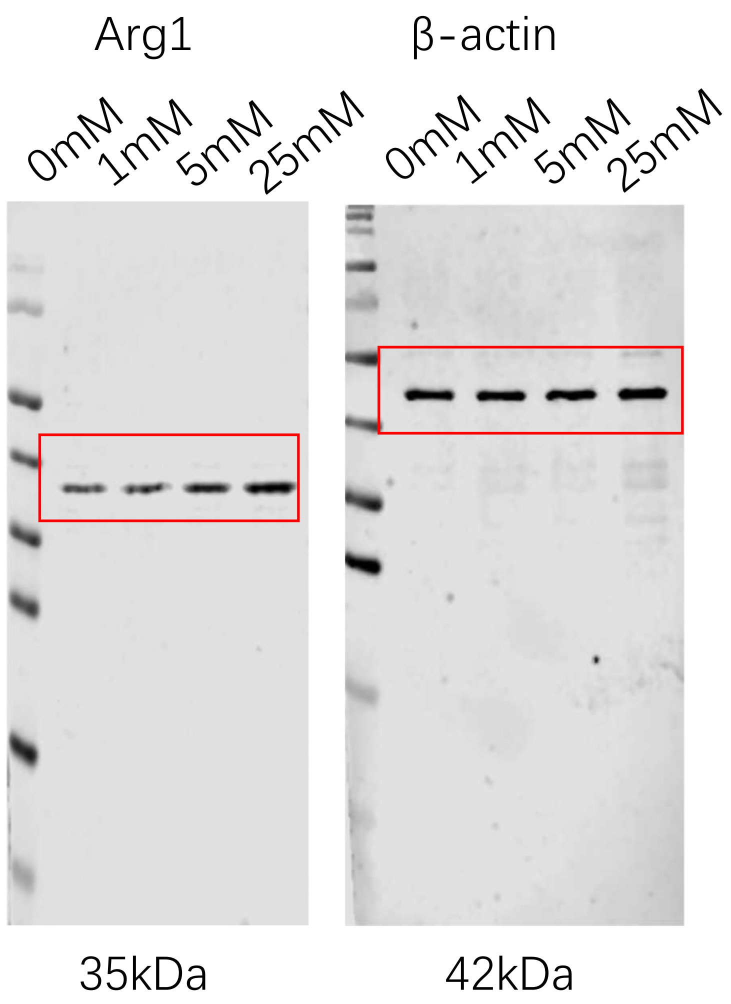

Figure7e

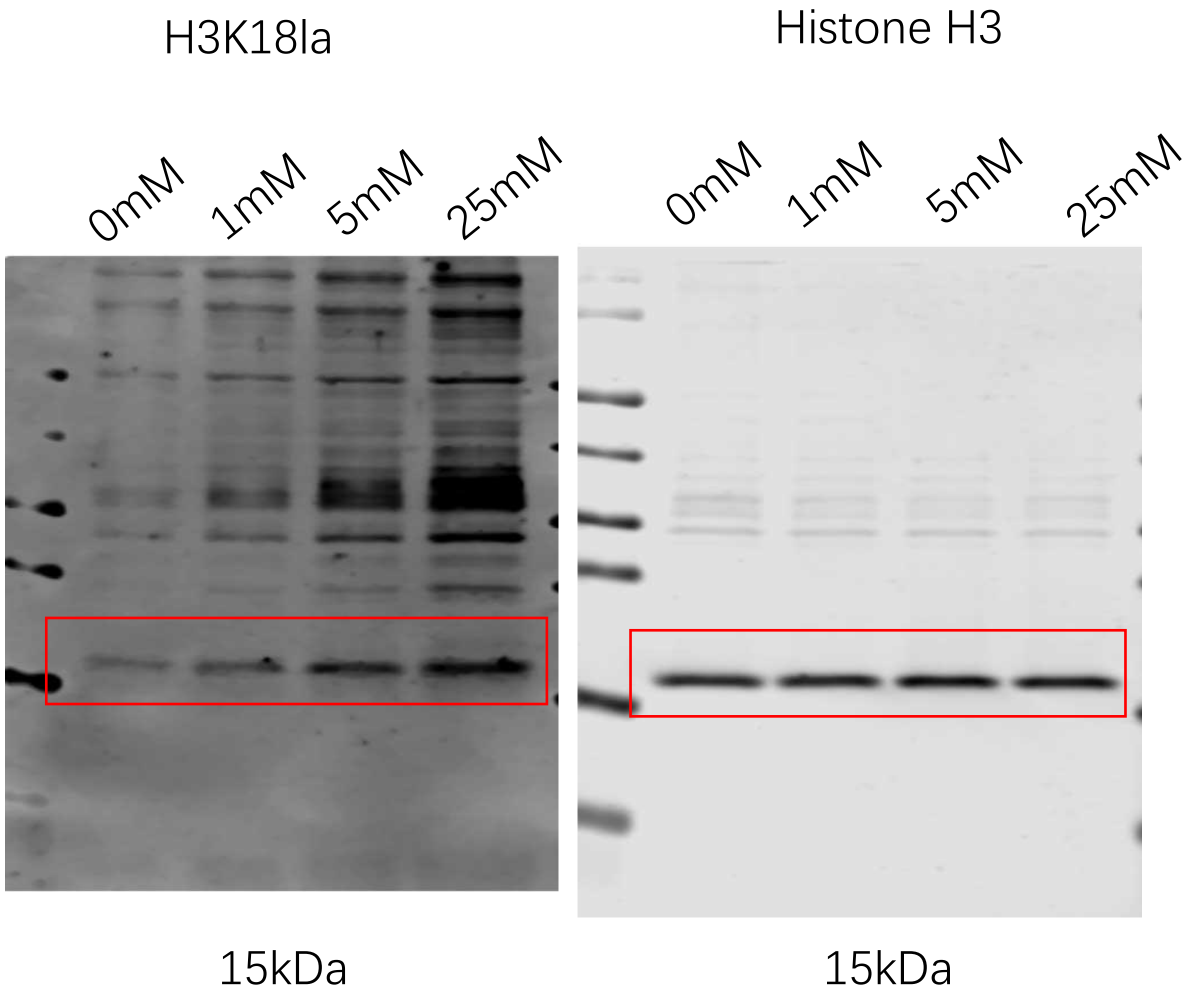
H3K18la
Histone H3
0mM
1mM
5mM
25mM
0mM
1mM
5mM
25mM
15kDa
15kDa

Figure7i

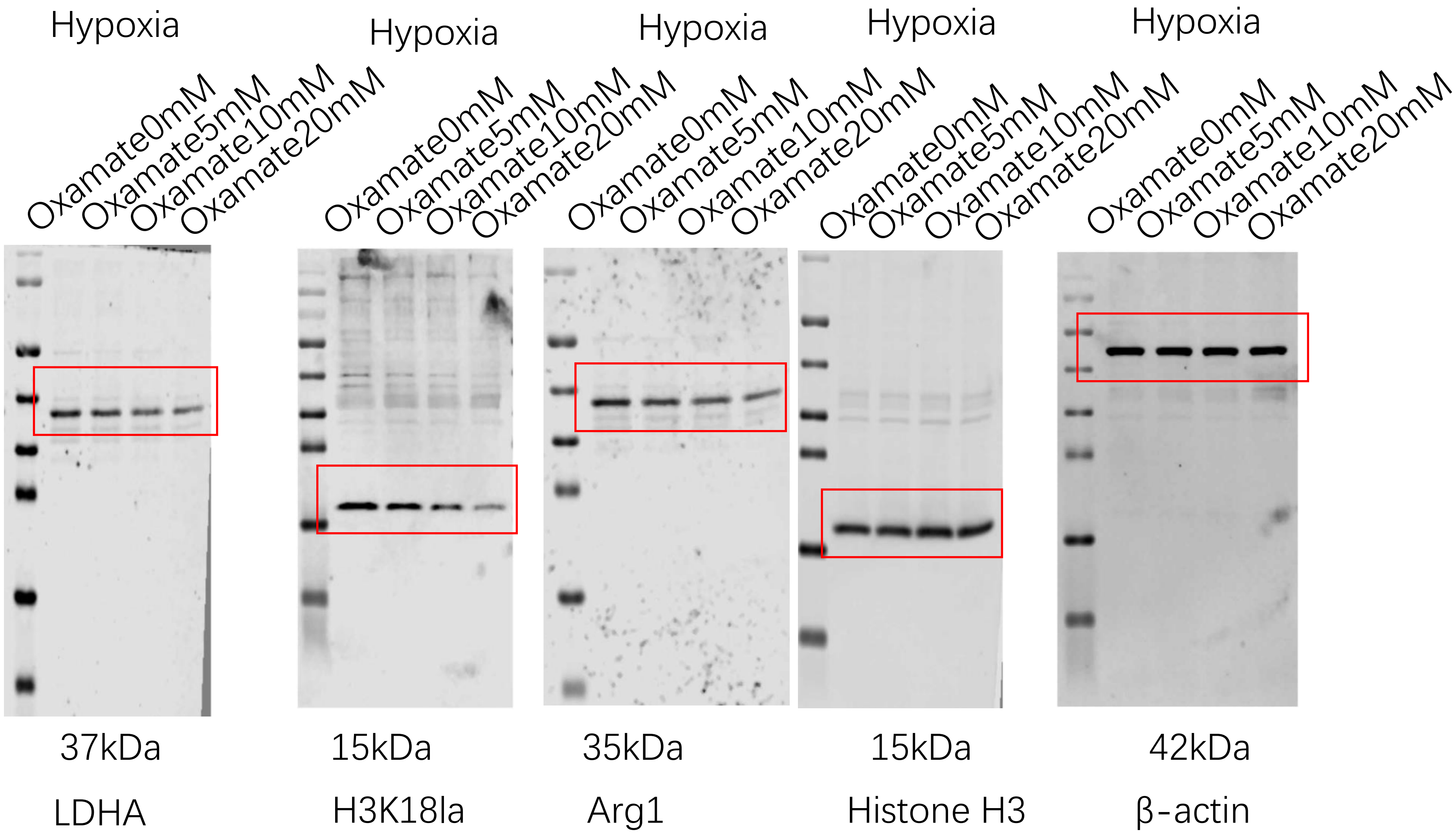
Hypoxia
Oxamate0mM
Oxamate5mM
Oxamate10mM
Oxamate20mM
Hypoxia
Oxamate0mM
Oxamate5mM
Oxamate10mM
Oxamate20mM
Hypoxia
Oxamate0mM
Oxamate5mM
Oxamate10mM
Oxamate20mM
Hypoxia
Oxamate0mM
Oxamate5mM
Oxamate10mM
Oxamate20mM
Hypoxia
Oxamate0mM
Oxamate5mM
Oxamate10mM
Oxamate20mM
37kDa
LDHA
15kDa
H3K18la
35kDa
Arg1
15kDa
Histone H3
42kDa
β-actin

Figure8f

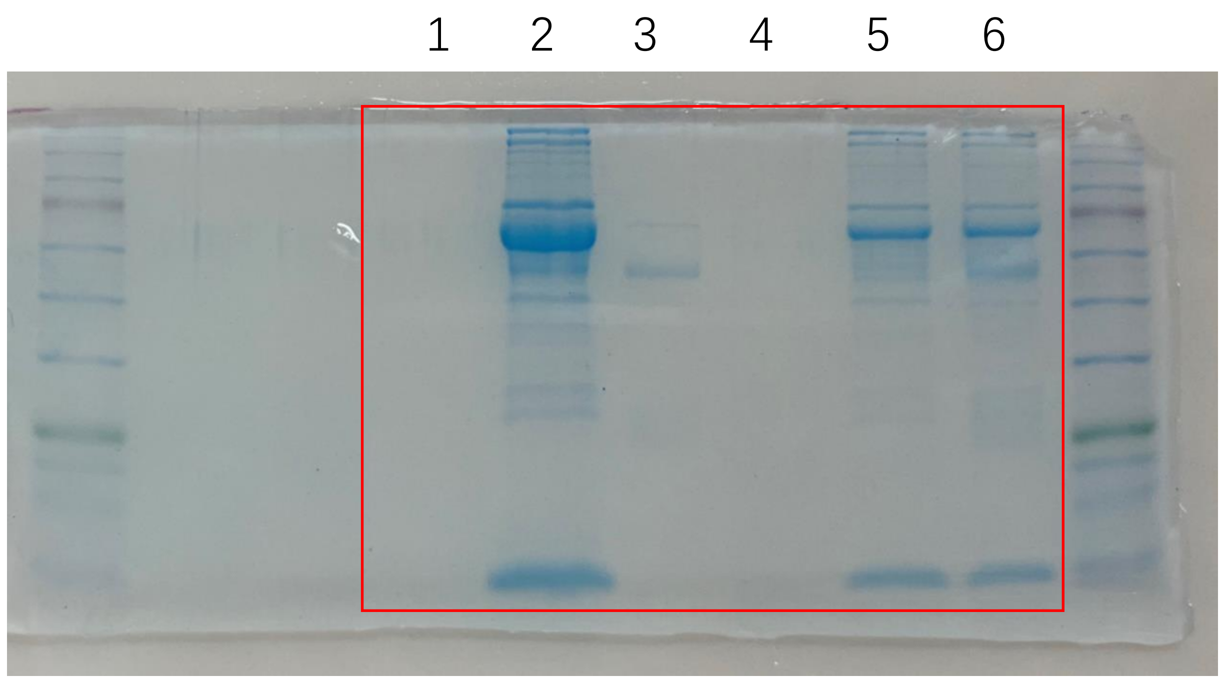

Figure8g

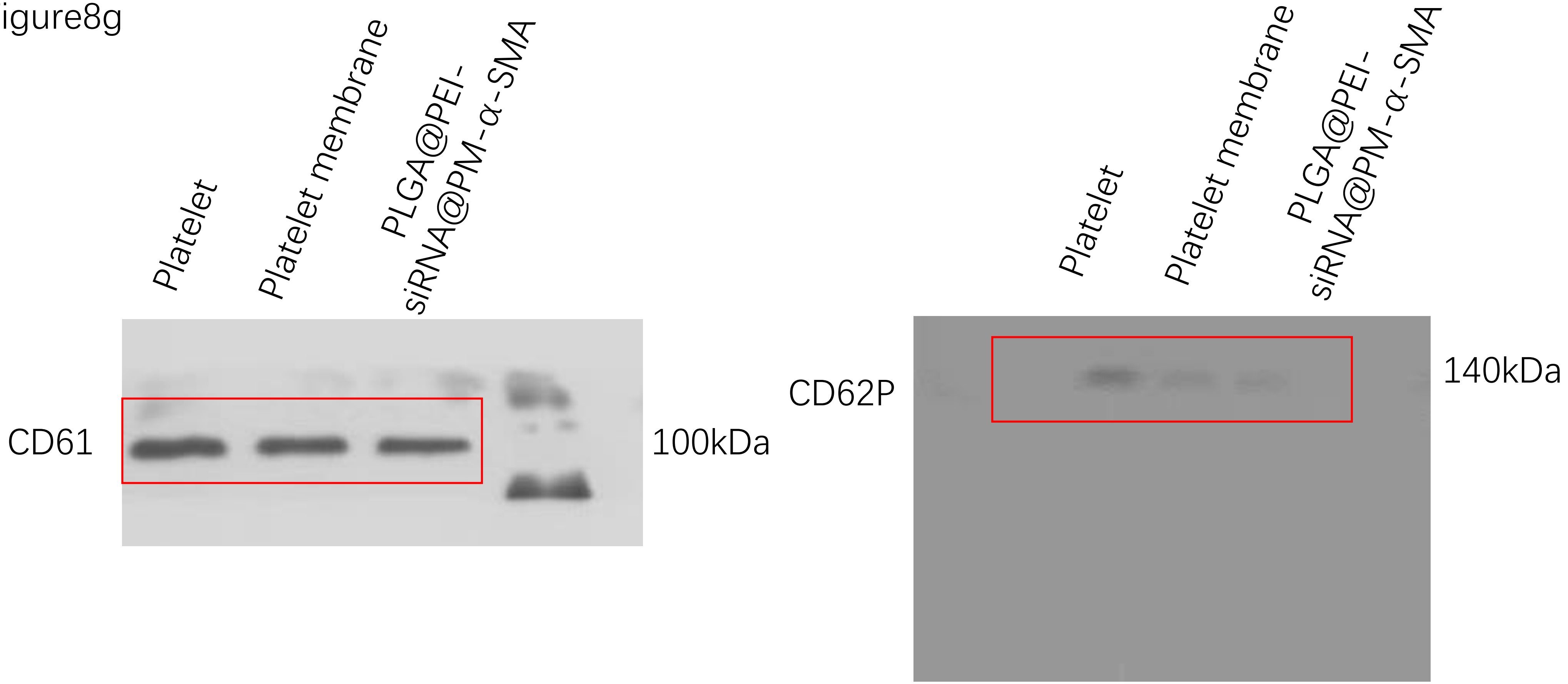
Platelet
Platelet membrane
PLGA@PEI-siRNA@PM-α-SMA
CD61
100kDa
Platelet
Platelet membrane
PLGA@PEI-siRNA@PM-α-SMA
CD62P
140kDa

Figure9a

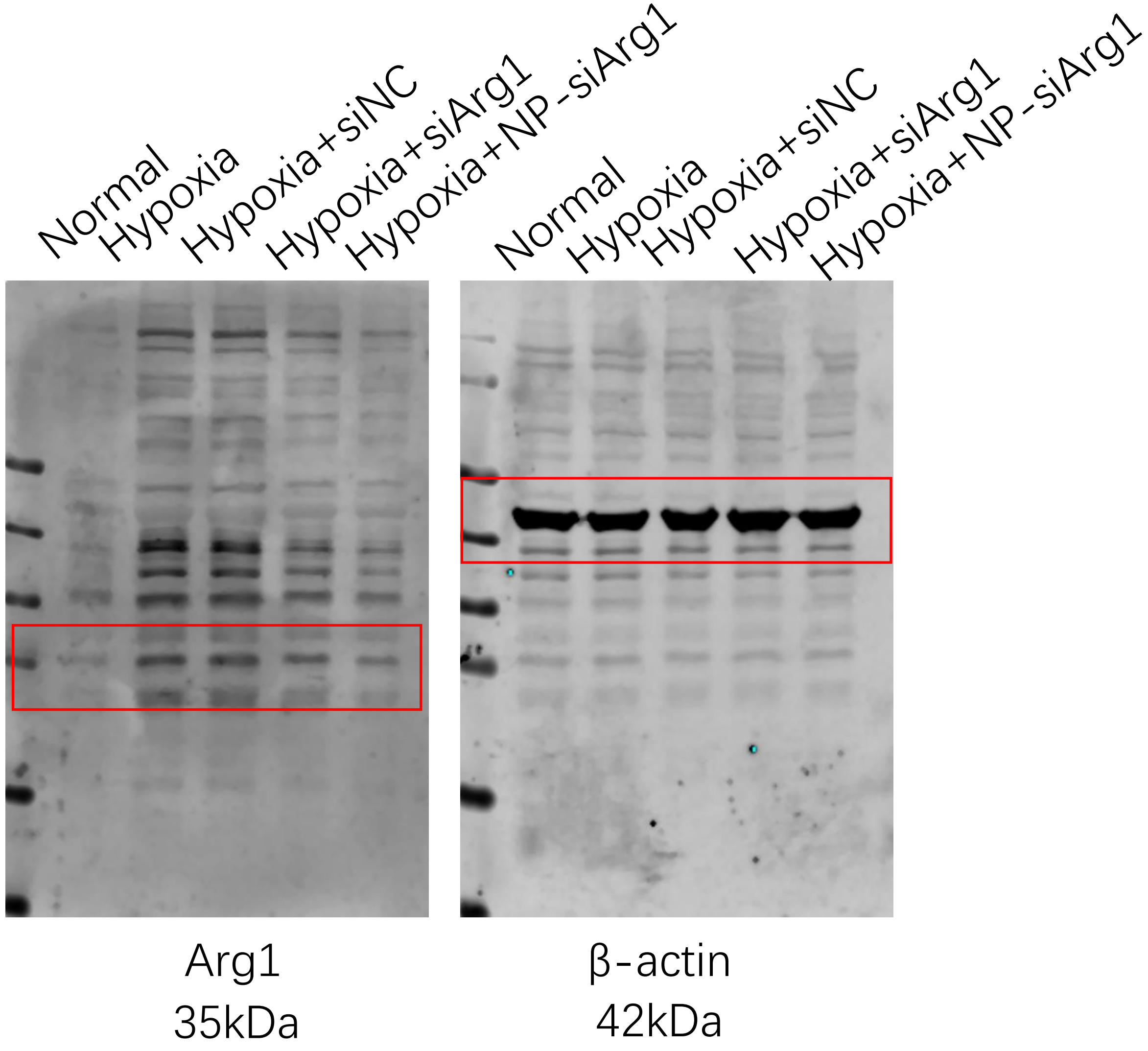
Normal
Hypoxia
Hypoxia+siNC
Hypoxia+siArg1
Hypoxia+NP-siArg1
Normal
Hypoxia
Hypoxia+siNC
Hypoxia+siArg1
Hypoxia+NP-siArg1
Arg1
35kDa
β-actin
42kDa

Figure9p

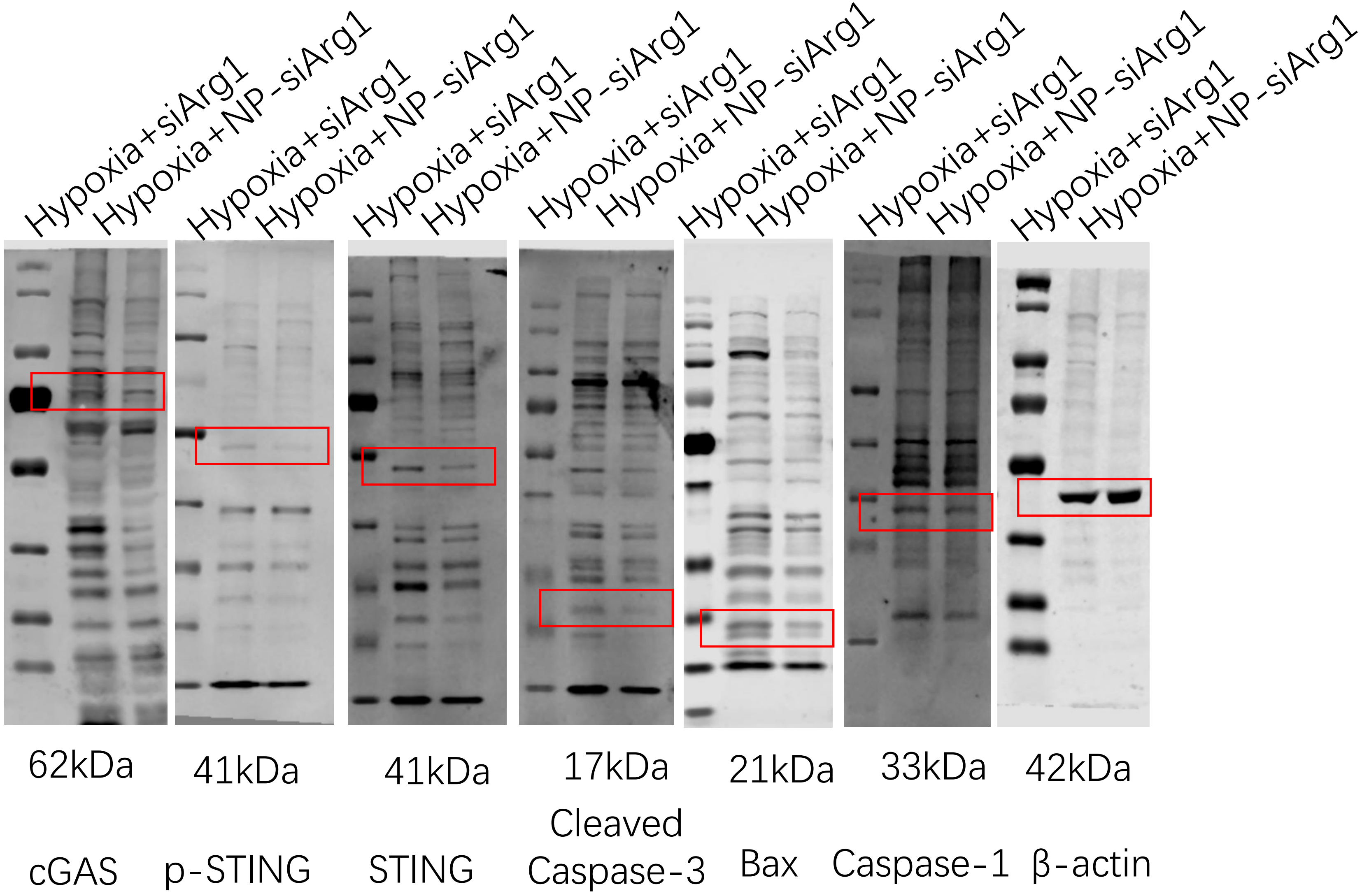
Hypoxia+siArg1
Hypoxia+NP-siArg1
Hypoxia+siArg1
Hypoxia+NP-siArg1
Hypoxia+siArg1
Hypoxia+NP-siArg1
Hypoxia+siArg1
Hypoxia+NP-siArg1
Hypoxia+siArg1
Hypoxia+NP-siArg1
Hypoxia+siArg1
Hypoxia+NP-siArg1
Hypoxia+siArg1
Hypoxia+NP-siArg1
62kDa
41kDa
41kDa
17kDa
21kDa
33kDa
42kDa
cGAS
p-STING
STING
Cleaved Caspase-3
Bax
Caspase-1
β-actin

Figure9p

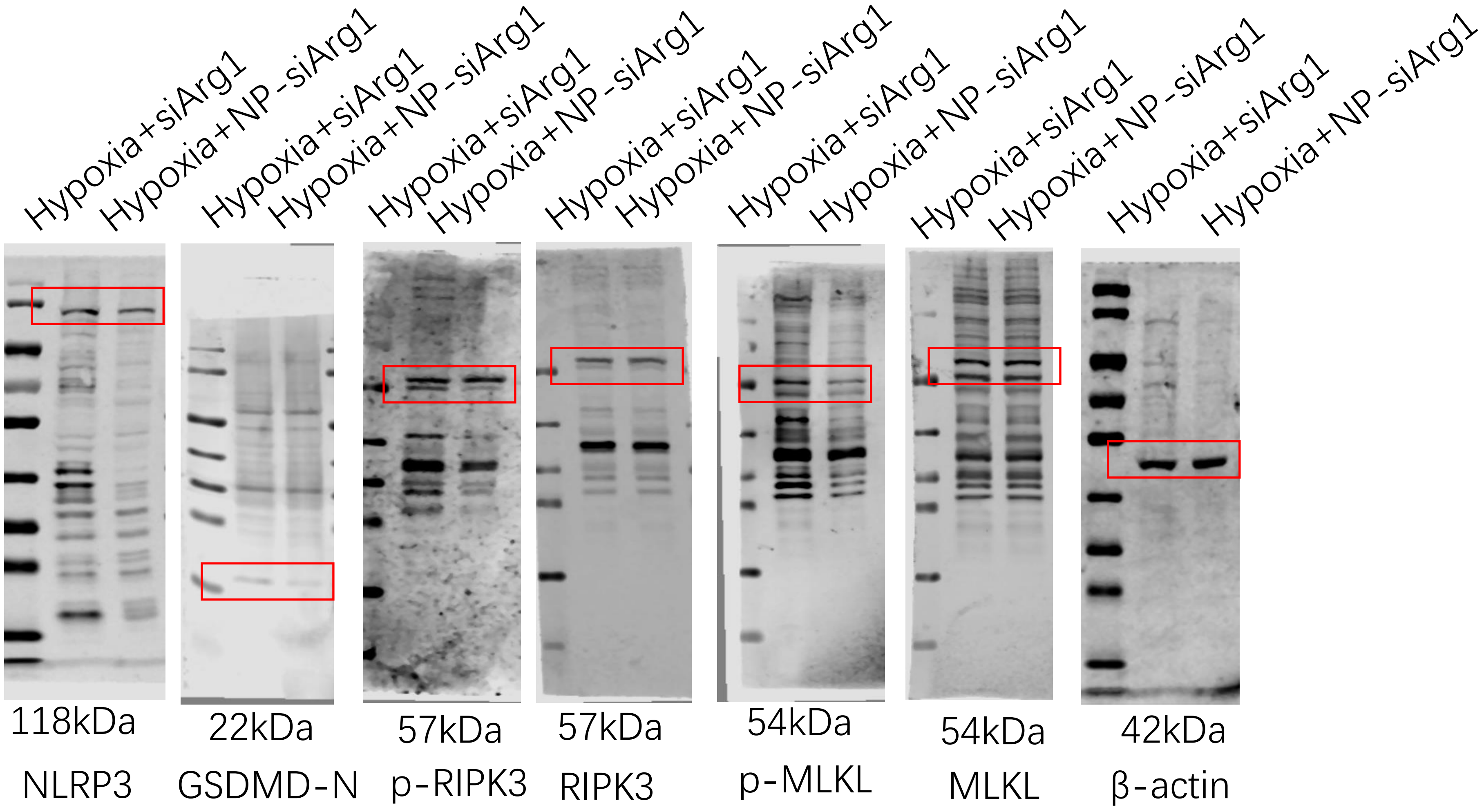
Hypoxia+siArg1
Hypoxia+NP-siArg1
Hypoxia+siArg1
Hypoxia+NP-siArg1
Hypoxia+siArg1
Hypoxia+NP-siArg1
Hypoxia+siArg1
Hypoxia+NP-siArg1
Hypoxia+siArg1
Hypoxia+NP-siArg1
Hypoxia+siArg1
Hypoxia+NP-siArg1
Hypoxia+siArg1
Hypoxia+NP-siArg1
118kDa
NLRP3
22kDa
GSDMD-N
57kDa
p-RIPK3
57kDa
RIPK3
54kDa
p-MLKL
54kDa
MLKL
42kDa
β-actin

Supplementary Figure1b

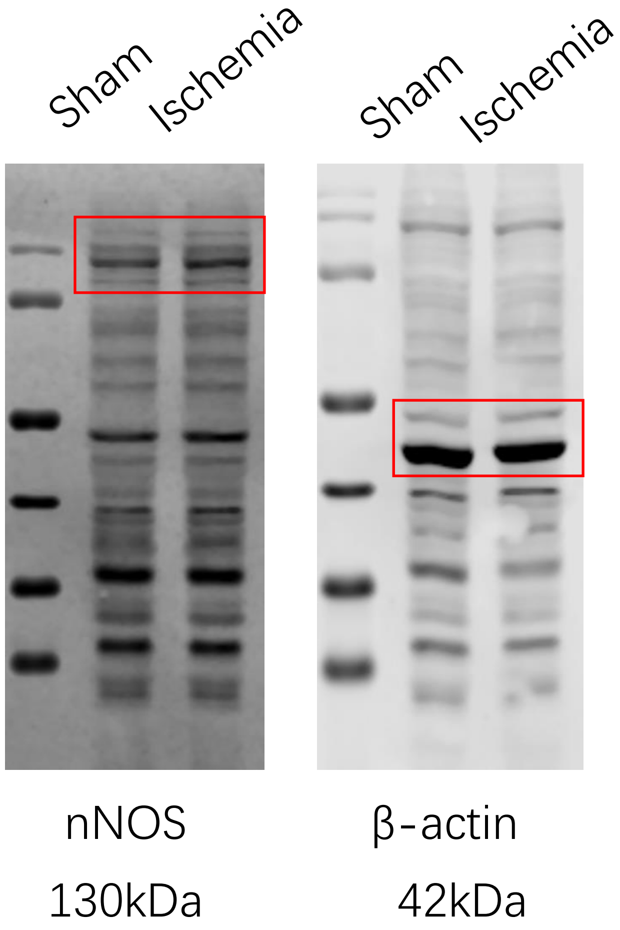

Supplementary Figure4a

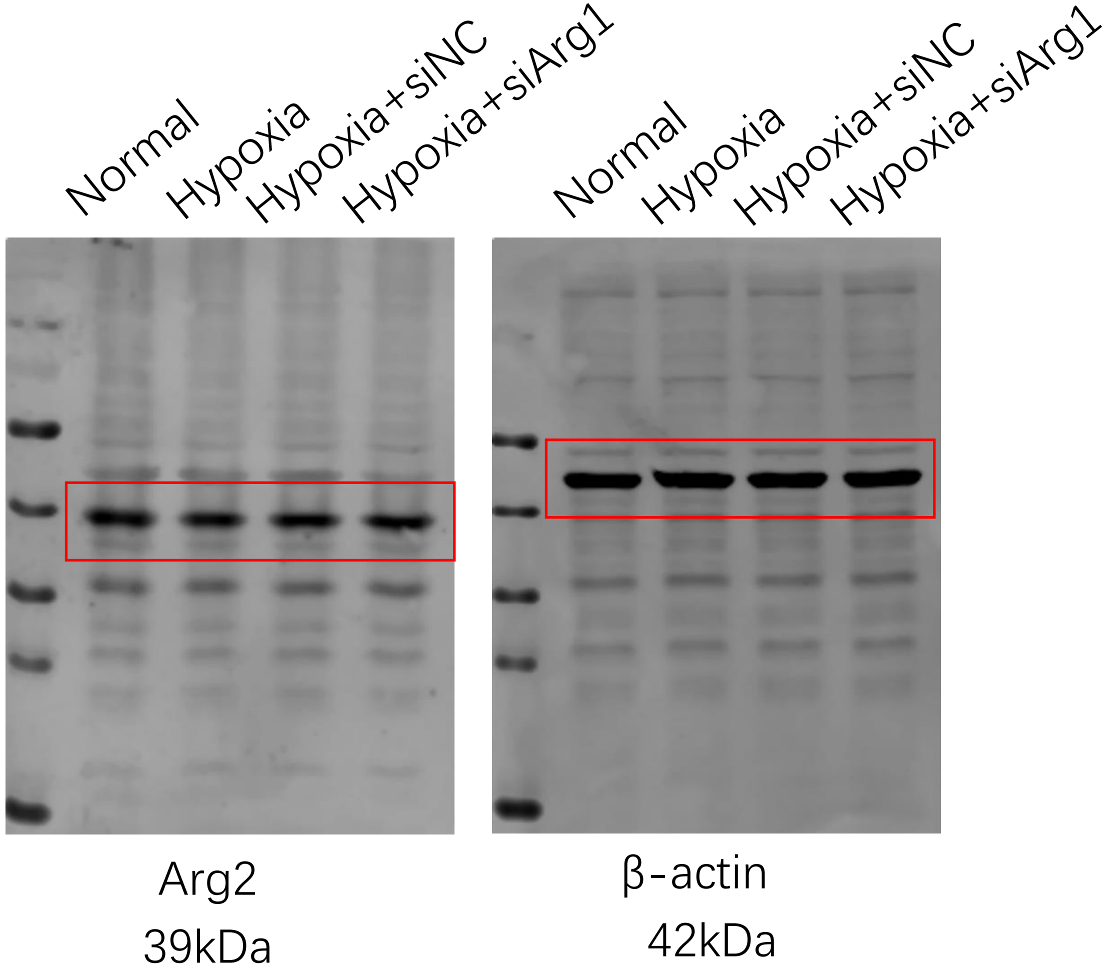

Supplementary Figure5c

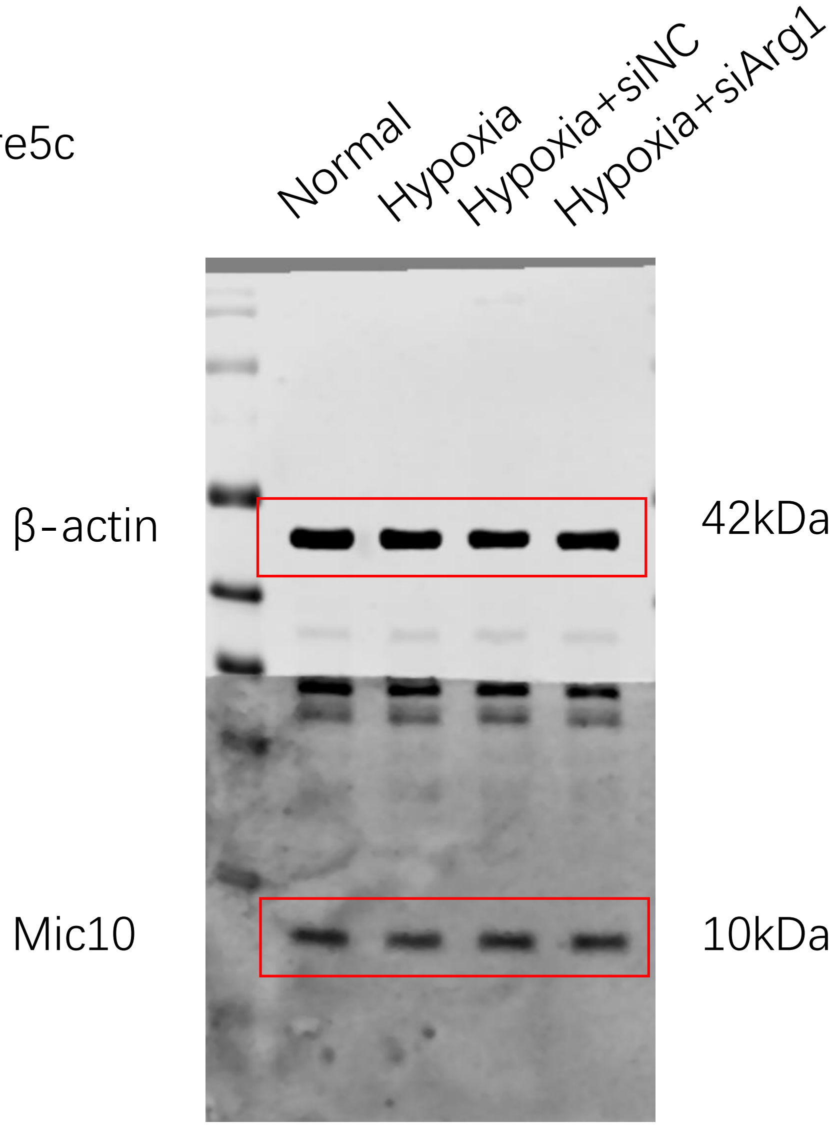

Supplementary Figure8d

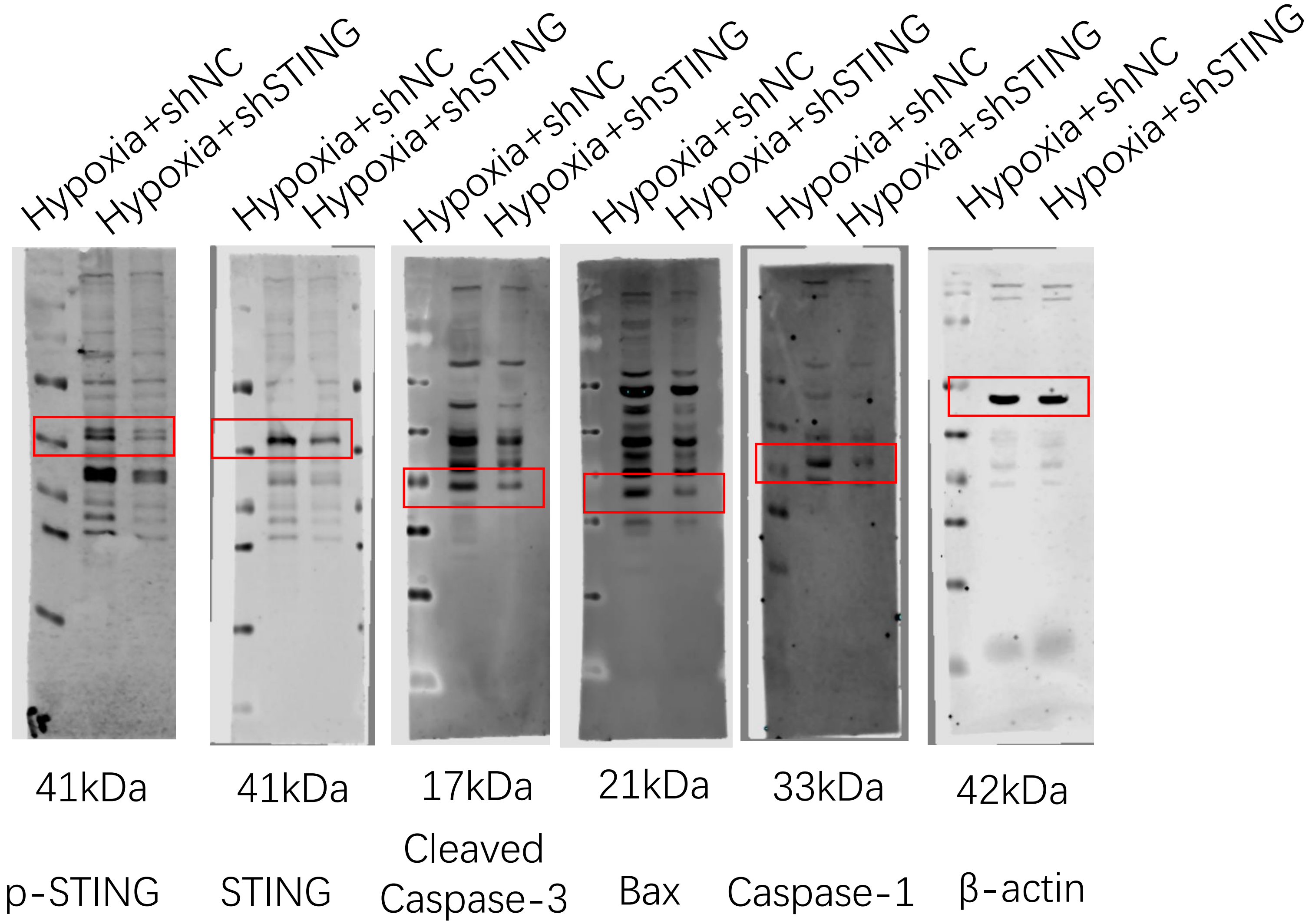

Supplementary Figure8d

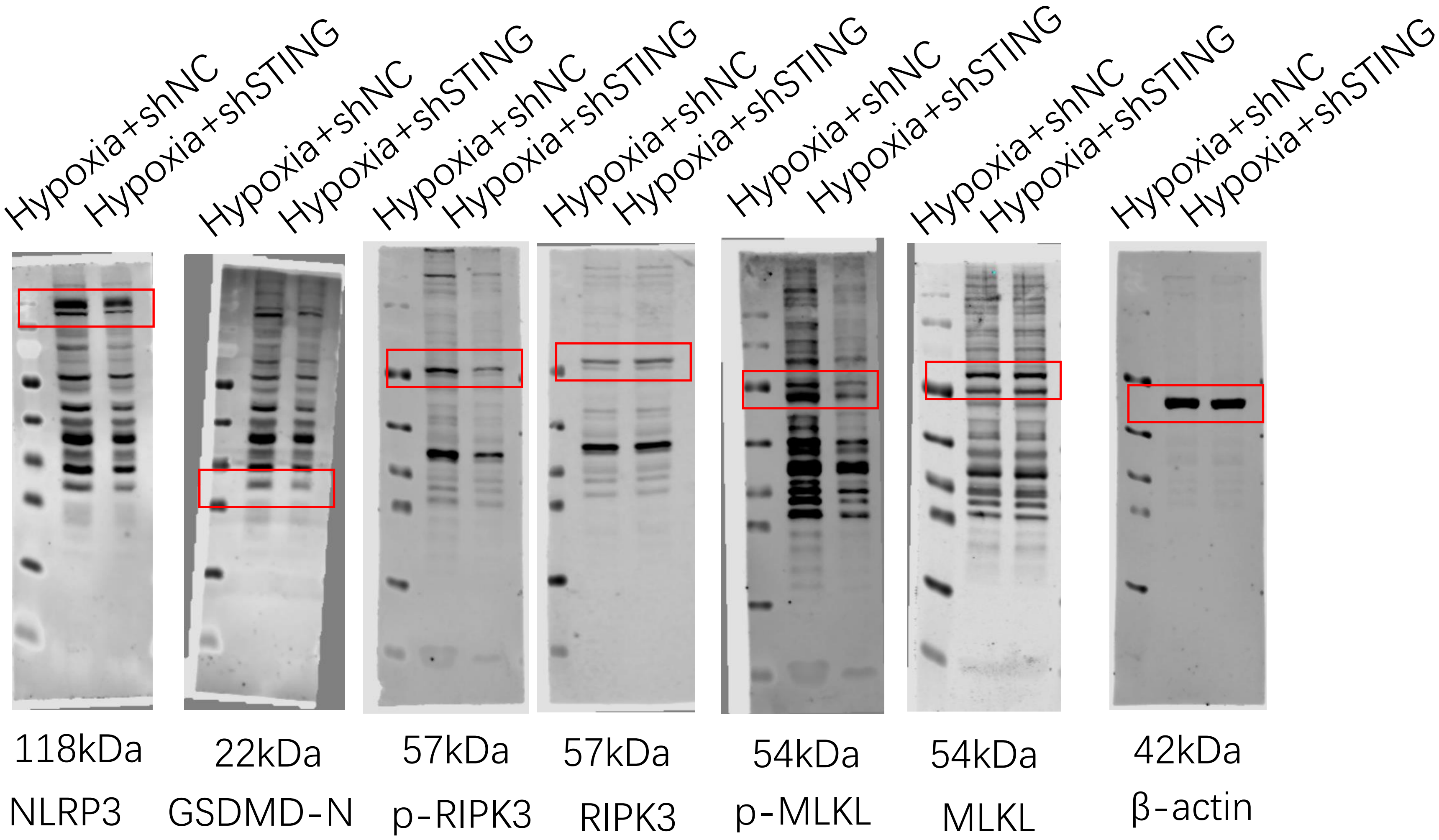

Supplementary Figure9a

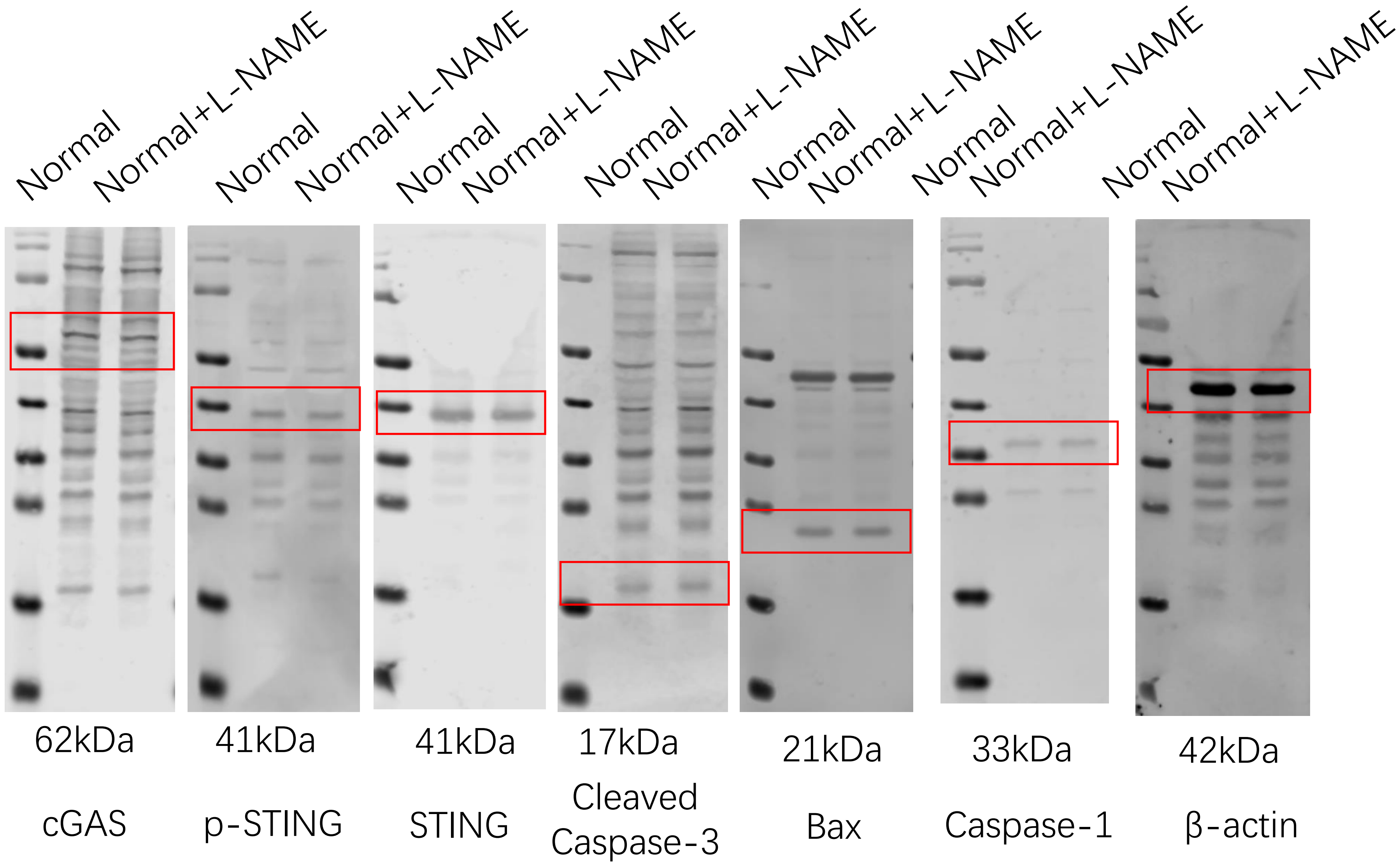

Supplementary Figure9a

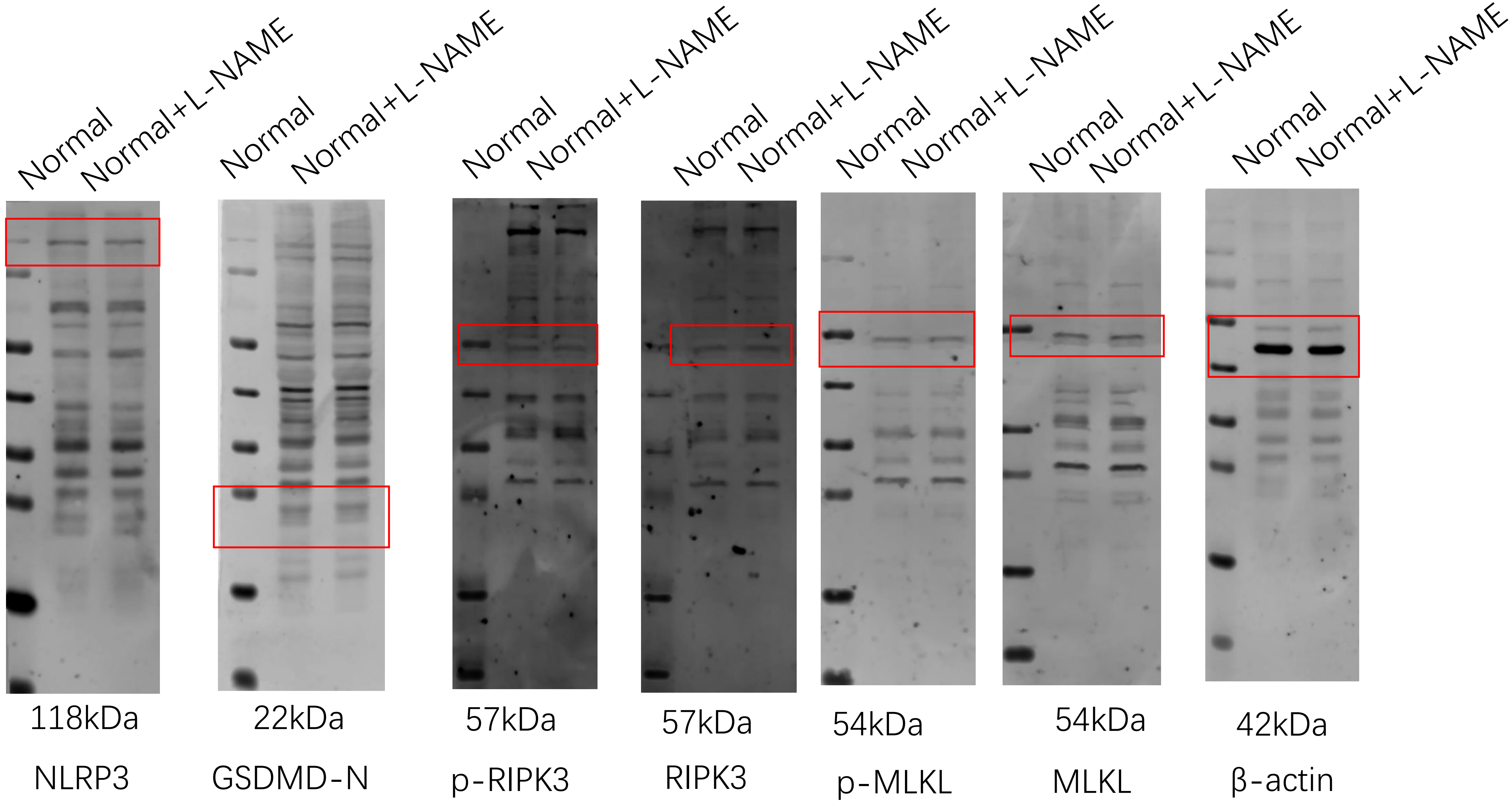

Supplementary Figure9c

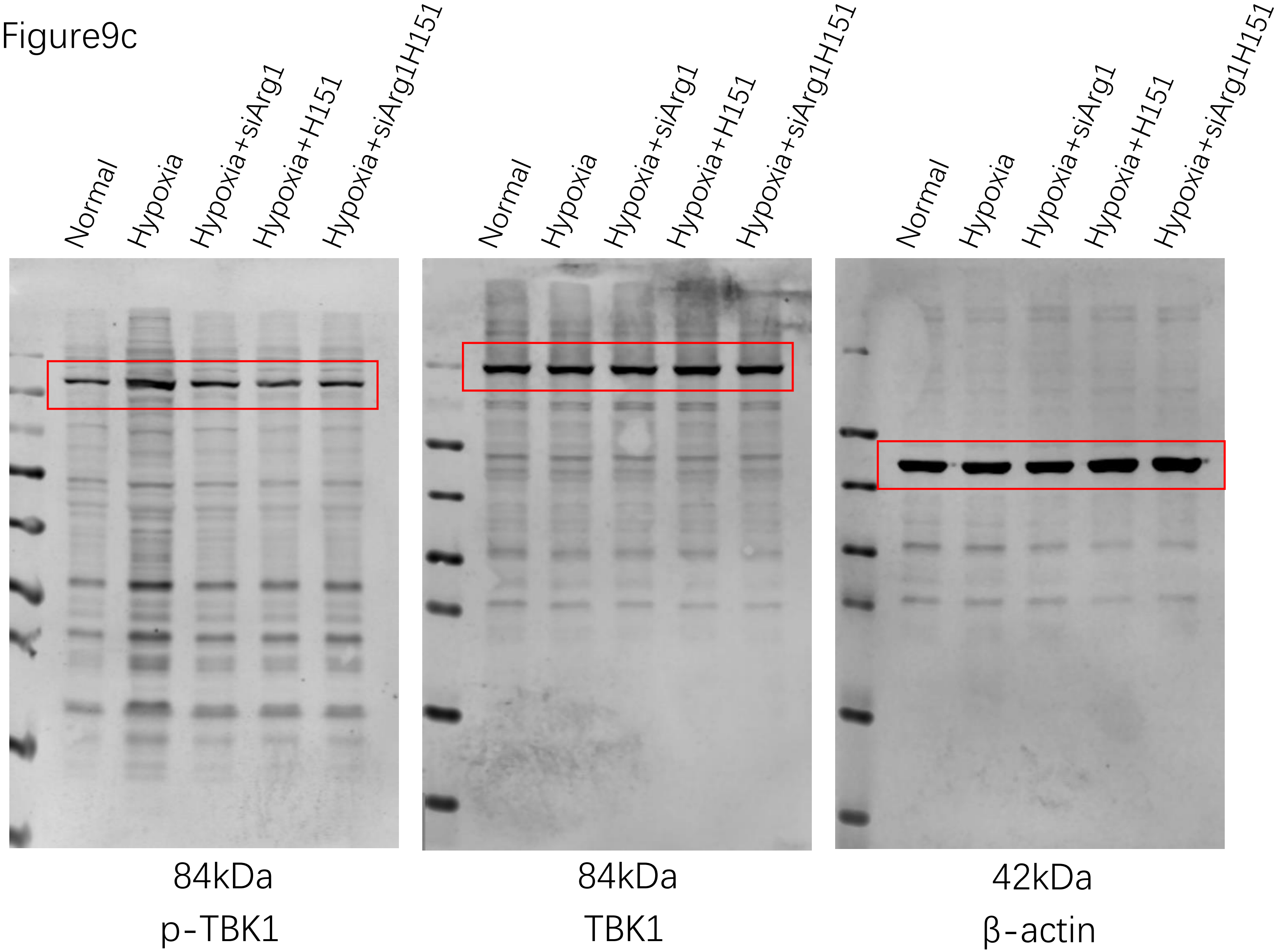

Supplementary Figure10d

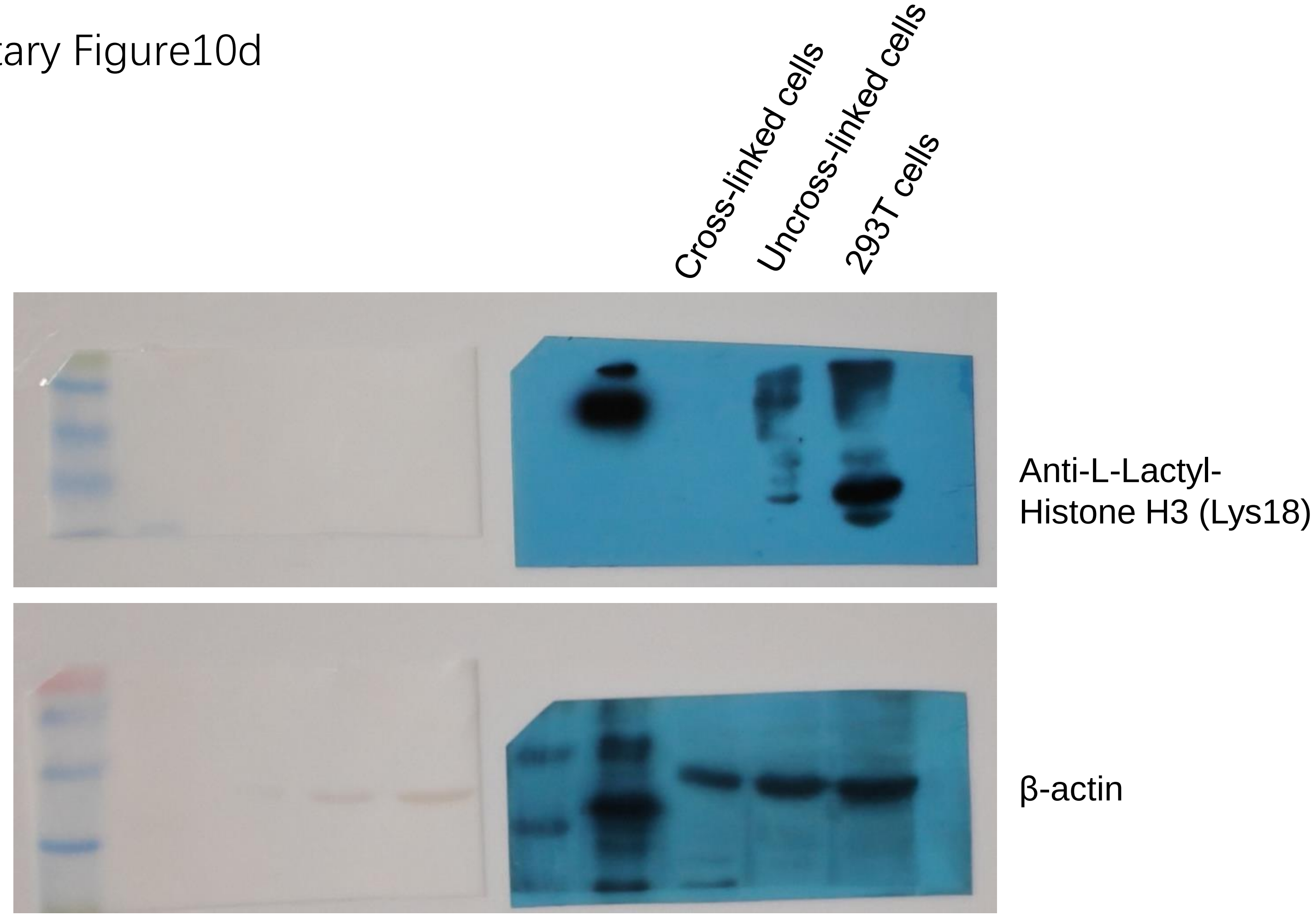

Supplementary Figure10e

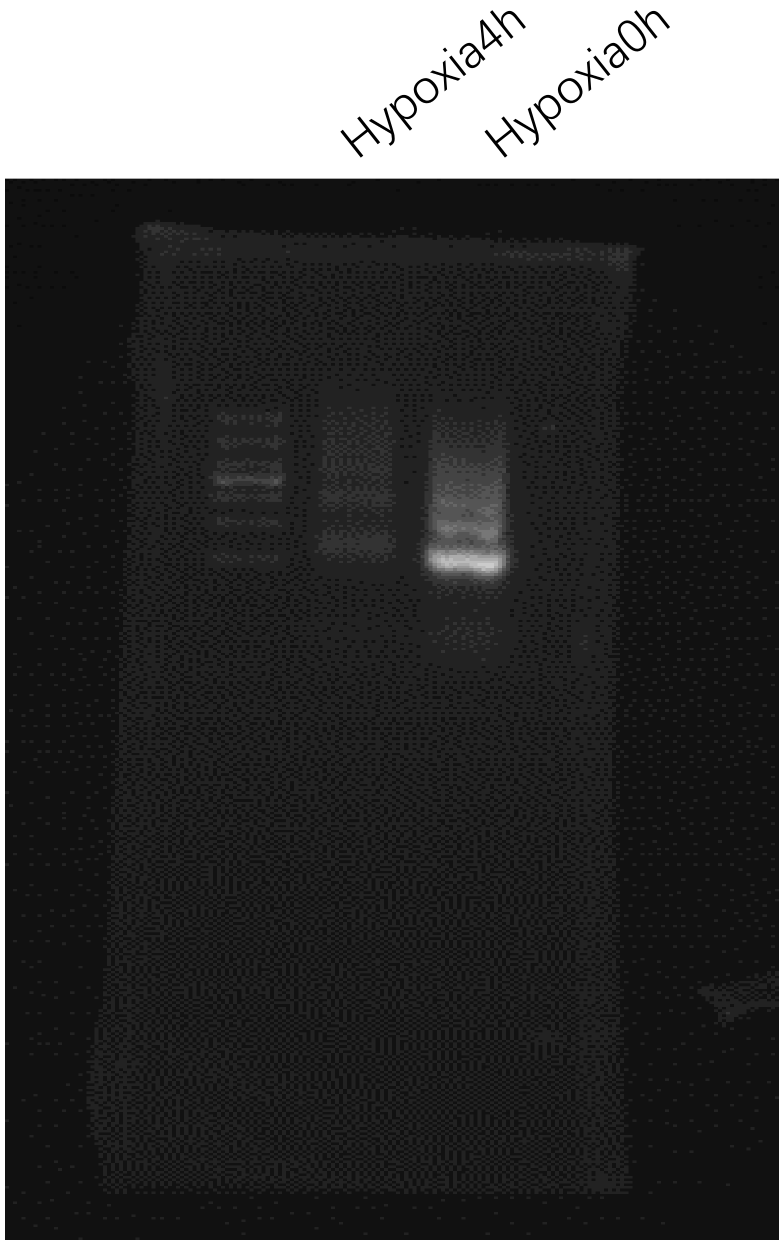

Supplementary Figure11a

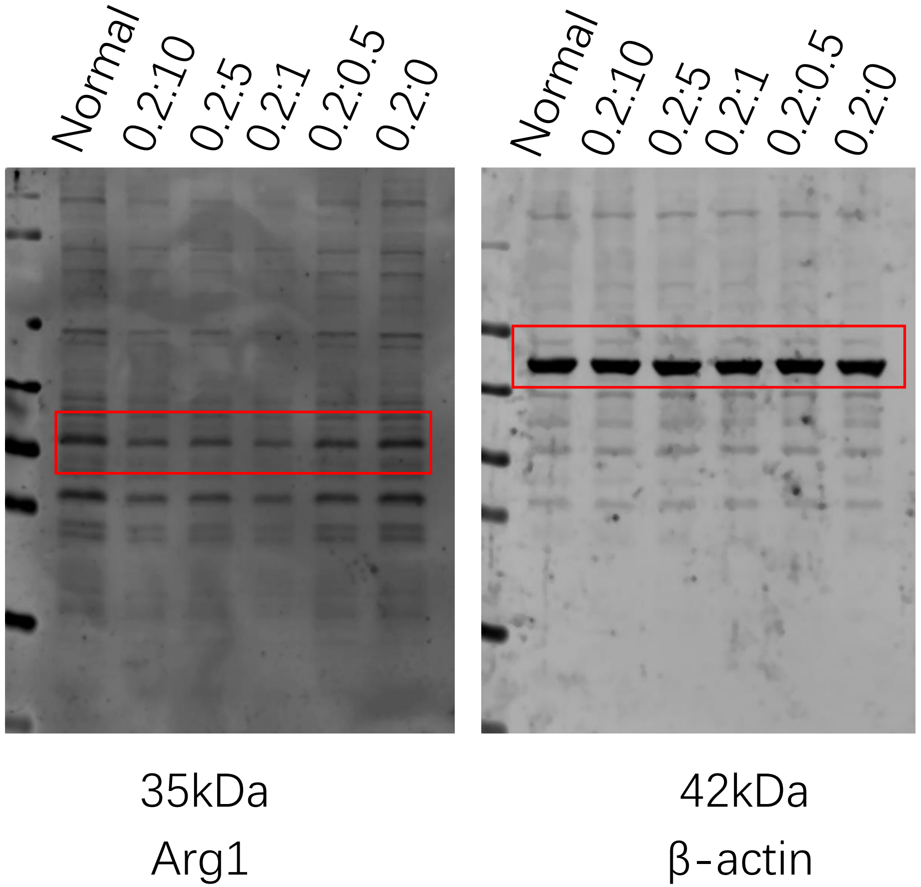

Supplementary Figure11e

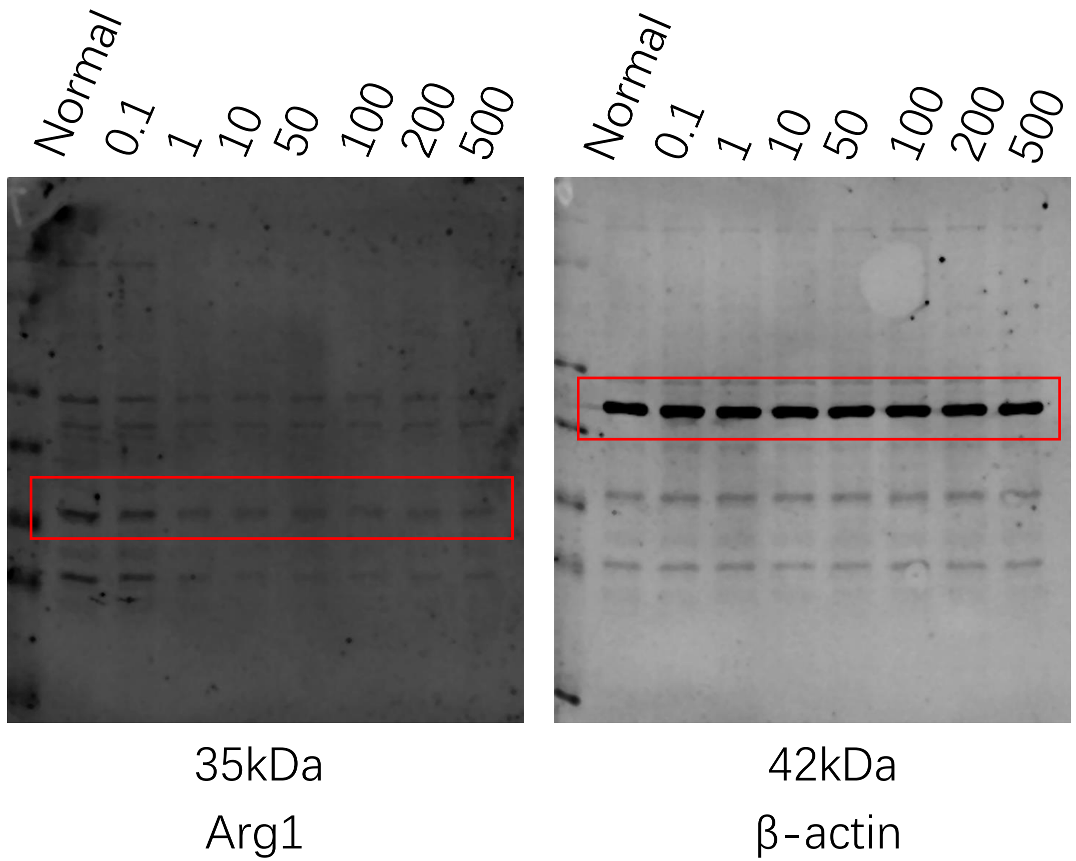

Supplement: Supplementary file 2 — Original images of Western blots [file 41392_2025_2255_MOESM2_ESM.pdf]
