# Supplementary material for: Arginase 1 drives mitochondrial cristae remodeling and PANoptosis in ischemia/hypoxia-induced vascular dysfunction
Source: Signal Transduct Target Ther. 2025 May 28;10:167. doi: 10.1038/s41392-025-02255-2 (PMC12117058; doi:10.1038/s41392-025-02255-2)
Supplement: Supplementary file 3 — Author Checklist for Research Article (20250219) [file 41392_2025_2255_MOESM3_ESM.pdf]

Author Checklist for Research Article（STTT）

Caution:

1. Please carefully check all figures and tables to ensure no mistakes, duplicates and inconsistency with main text and corresponding legends.

2. Please add the statement “All authors confirm that there are no mistakes, duplicates and inconsistency with main text and corresponding legends in the figures and tables” in the Rebuttal Letter of the final round revision.

3. Any data misuse or figure manipulation found after formal publication will be considered as misconduct and processed as retraction. Please pay attention to this.

Contents

Article structure: 2

Title page: 3

Supplementary data 4

Data availability statements, Acknowledgements, Conflict of interests, Contributions, References. 5

Figures: 6

Others tips: 14

For synthesized compounds 15

For crystal structures: 16

For Clinical trials: 17

Article structure:

- Please note that **Articles** must contain the following components in the order stated. Please see below for further details. **Title page, Abstract, Introduction, Results, Discussion, Materials and methods, Data availability statements, Acknowledgements, Conflict of interests, Contributions, References.**
- **No abbreviation list** in the manuscript. Abbreviations and acronyms must be defined the first time they are used and then used throughout the remainder of the manuscript. **Please avoid using uncommon abbreviations throughout full text.**
- The **title** is suggested to be in no more than **15** words.
- The **title** should avoid use of jargon, abbreviations, and punctuation; **if abbreviation is indispensable, please put full name first and abbreviation in the brackets**.
- **Abstract** should be unstructured and **200-250** words.
- **Introduction** should include **4** paragraphs (around 800 words): (1) Discuss previous and/or current research in the field. (2) Establish the importance of the current topic. (3) Identify the problem and explain the approach taken to address it. (4) Briefly describe the present paper, highlighting why your study is new in the subject field and why it is important.
- **Discussion** should include **5** paragraphs (around 1000 words): (1) Address the problem stated in your introduction. (2) Review your findings in the context of supporting literature and existing knowledge. (3) Critically analyze your results. (4) Discuss potential applications in targeted therapy or future research directions. (5) Derive conclusions.
- Please confirm that the supplementary data follows our template. You may find the supplementary template on our website: https://www.nature.com/sigtrans/authors-and-referees/online-submission

Title page:

- **No Graphic Abstract.**
- **Authorship (**no more than 3 corresponding authors and no more than 5 co-first authors).
- Please **double check** the authorship (including but not limited to the **spellings of all authors' names**, the **order of the author list**, and the **markings of all co-first and co-corresponding authors**) in both the manuscript files uploaded and the online submission system, to **ensure no mistakes**.

  **Any changes to authorship will not be allowed once the manuscript has been accepted for publication. Correction Note regarding authorship changes will not be allowed either after formal publication.
- Please upload the "**Change of Authorship Request Form**" when submitting the revision if any changes concerning the authorship (including but not limited to: **removal** and/or **addition** of any authors, **change of authors' order**, etc.) are made. You can download this form at: https://resource-cms.springernature.com/springer-cms/rest/v1/content/7454878/data/v5
- **Affiliations including ones in Hong Kong, Macau/Macao and Taiwan/Taipei**: pay attention that the last **must be** "Hong Kong, China" or "Macau/Macao, China" or "Taiwan/Taipei, China" (**Caution: The Comma is NECESSARY!**).

Supplementary data

- **Supplementary data** should be cited as "supplementary Fig or Table" in the main text. Please re-confirm that the supplementary data follows our template. You may find the supplementary template on our website: https://www.nature.com/sigtrans/authors-and-referees/online-submission

Data availability statements, Acknowledgements, Conflict of interests, Contributions, References.

- **Data availability statements** section should be placed after the Materials and Methods section.
- **Mandated data types.** For the following data types: **DNA/RNA/Protein sequence data, Genome assembly data, Proteomics data, Genetic variation data, Functional genomics data, Macromolecular structure data, Gene expression data, Crystallographic data for small molecules**, submission to a community-endorsed, public repository is mandatory. Persistent identifiers (DOIs and accession numbers) assigned to the data by the repository must be appropriately cited and referenced in the published article. You may find the research-data-policy here:
  https://www.springernature.com/gp/authors/research-data-policy/repositories-socsci/19540364
- **All Animal experiments** should be approved by an ethical committee.
- **Ethics approval and consent to participate**: this study collected serial blood and urine samples from the human subjects. Please declare if this study was approved by an ethics committee (**full name needed**), and whether informed consents were obtained from the participants before the study.
- **All the ethics approval statements** related to human and/or animal experiments should be put in the **main-text Materials and Methods** section (it is **strongly recommended** to establish a separate part at the beginning of **Materials and Methods** for **all these ethics statements**).
- Please add the statement "All authors have read and approved the article" in the **Contributions** part.
- **Reference style** (All authors should be listed for papers with up to five authors; for papers with more than five authors, the first only should be listed, followed by et al. Abbreviations for titles of medical periodicals should conform to those used in the latest edition of Index Medicus. The first and last page numbers for each reference should be provided.) DOI number is not need and the journal names should be abbreviation.

Figures:

- Please draw all the **histogram, line and dot** plots in color. Particularly, **please change the bars in the bar chart to solid bars instead of hollow ones** (**Please refer to Example 1**). Black and white with patterns are not recommended in our journal.
- **Error bars** must be displayed including distinct dots for each measurement in the bar chart (**Please refer to Example 1**).
- STTT's standard figure sizes are 90 mm (single column) and 180 mm (double column) and the full depth of the page is 170 mm.
- Figure number should be placed in top left corner of each figure.
- **Figures** should be saved in RGB color mode at 300 dpi or higher resolution at publication size. Figures should be uploaded as single JPG or TIFF files. **DO NOT** submit figures in PDF format or embed figures into Word files.
- Please use the same typeface (Arial or Helvetica) for all figures. Use symbol font for Greek letters. Lettering in figures (labelling of axes and so on) should be in lowercase, with the first letter capitalized and no full stop.
- **Figures** are best prepared at the size you would expect them to appear in print. At this size, the optimum font size is 8pt and no lines should be thinner than 0.25 pt (0.09 mm). All words in figures must be clear at publication size. You may print the figures with A4 paper (vertical printing) to check the font size. Please pay attention to the **coordinates**, re-draw the **coordinates if** necessary**. (Please refer to Example 4)**
- **Figures** divided into parts should be labelled with a lower-case, boldface 'a', 'b', etc. in the top left-hand corner.
- In **ALL figures**, **units should have a single space between the number and the unit**.
- **Figure panels** in each figure should be no more than 26.
- All error bars and statistics must be defined in the figure legend (for example, "n=10 per group", "Data are represented as mean ± SEM"). Unusual units and abbreviations should be defined in the legend.

- Scale bar must be clear and mentioned in the figure legends. (**Please refer to Example** 5). Scale bars should be used rather than magnification factors. "**µ**" should not be written as "**u**".
- Image **rotated or scaled** must be the **same in the x and y dimensions**.
- **Figures** (or parts of figures) generated from online tools (e.g., **BioRender, etc**) or including online picture resources (e.g. from **SMART - Servier Medical ART, etc**) should be clearly indicated by **citing the original websites in the figure legend** or following the requirements mentioned in the original websites.
- For **chemical structure**, please follow the Style guide for chemical structures of nature research. You may find the guideline here: https://www.nature.com/documents/nr-chemical-structures-guide.pdf
- **For western blot, molecular weight label should follow the format below and black frame surrounding the blot is required**:

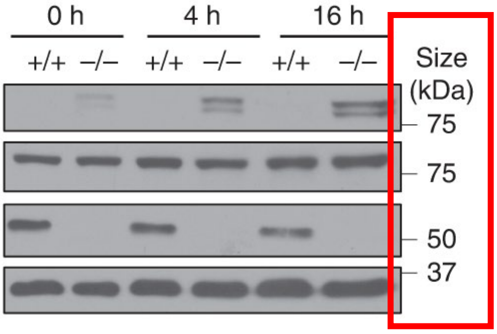

- It is **strongly suggested** to provide **ALL original and uncropped films of Western blots** with labeling of molecular weight, sample names and corresponding figures in the supplementary materials.

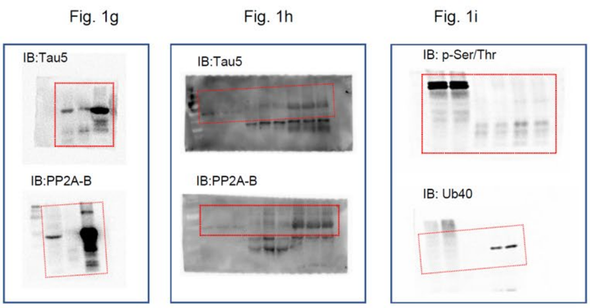

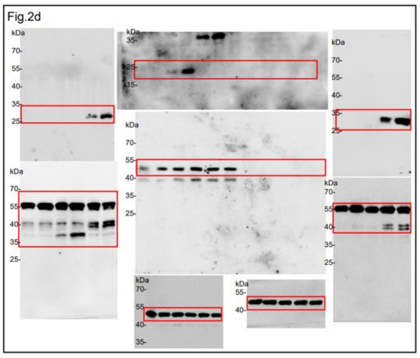

■ **For immunofluorescence images, nucleus staining is necessary for cell localization.**

■ **For Flow cytometry,** 1. Numbers in x- and y-axis should be **re-typed in larger fonts** when you arrange the figure and **should not be deleted**; 2. It is recommended to provide the **gating strategies** in the Supplementary Materials.

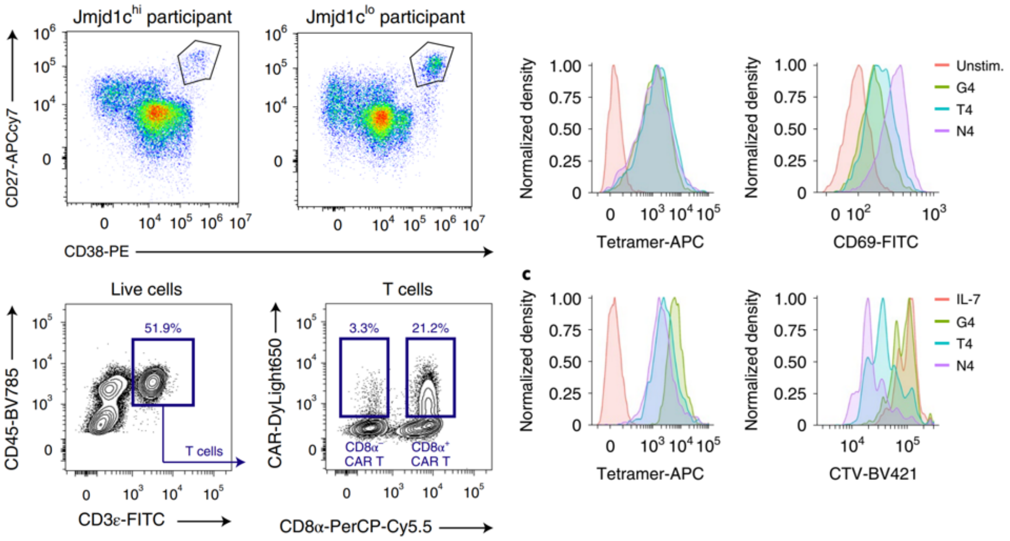

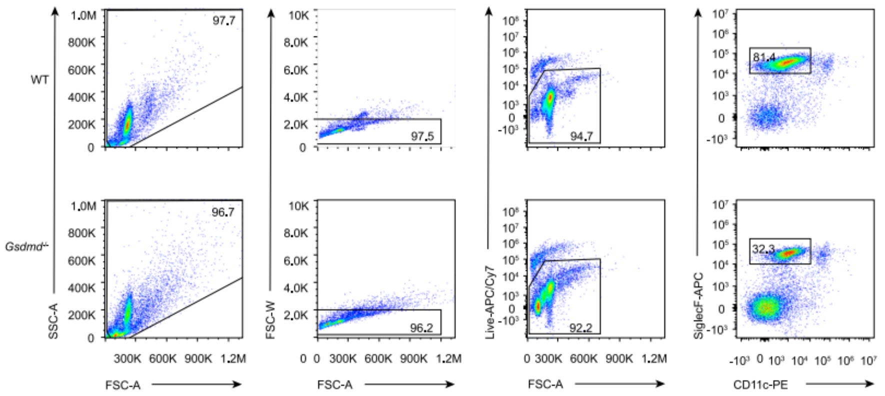

b

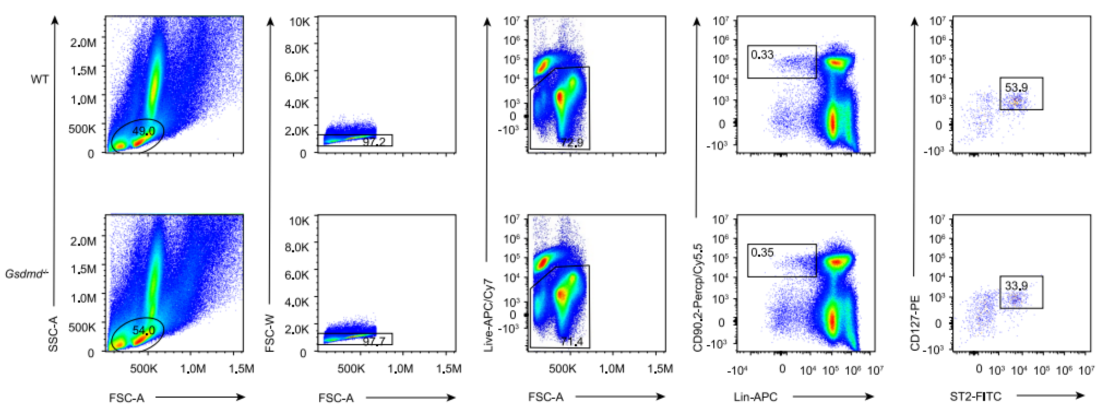

Figure style

Example 1:

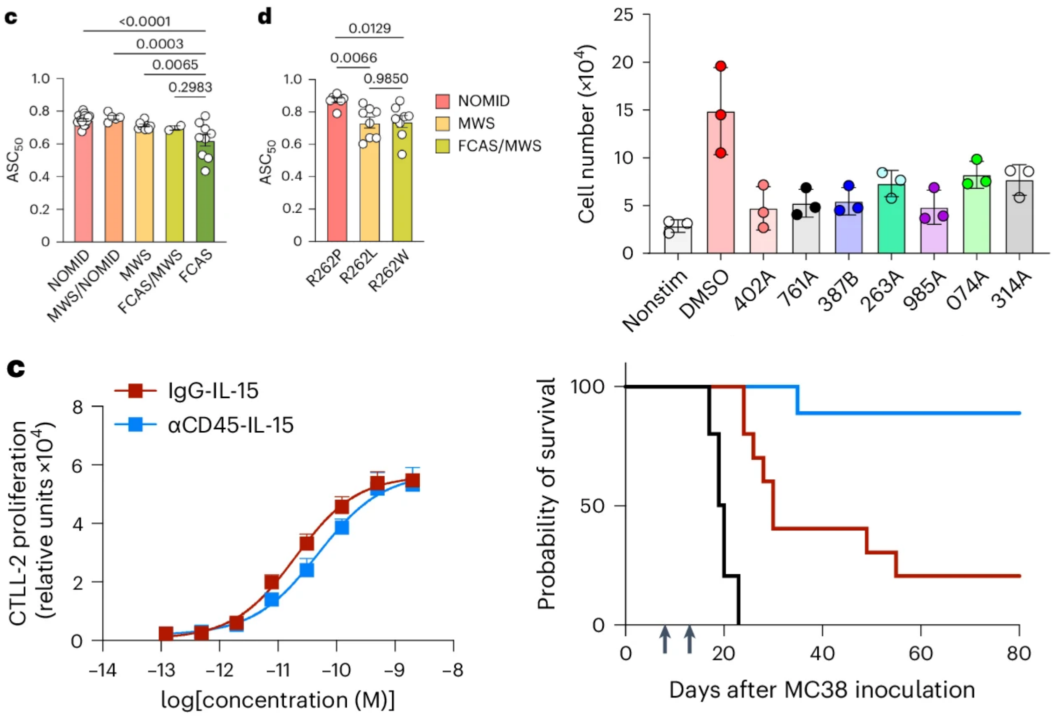

Example 2:

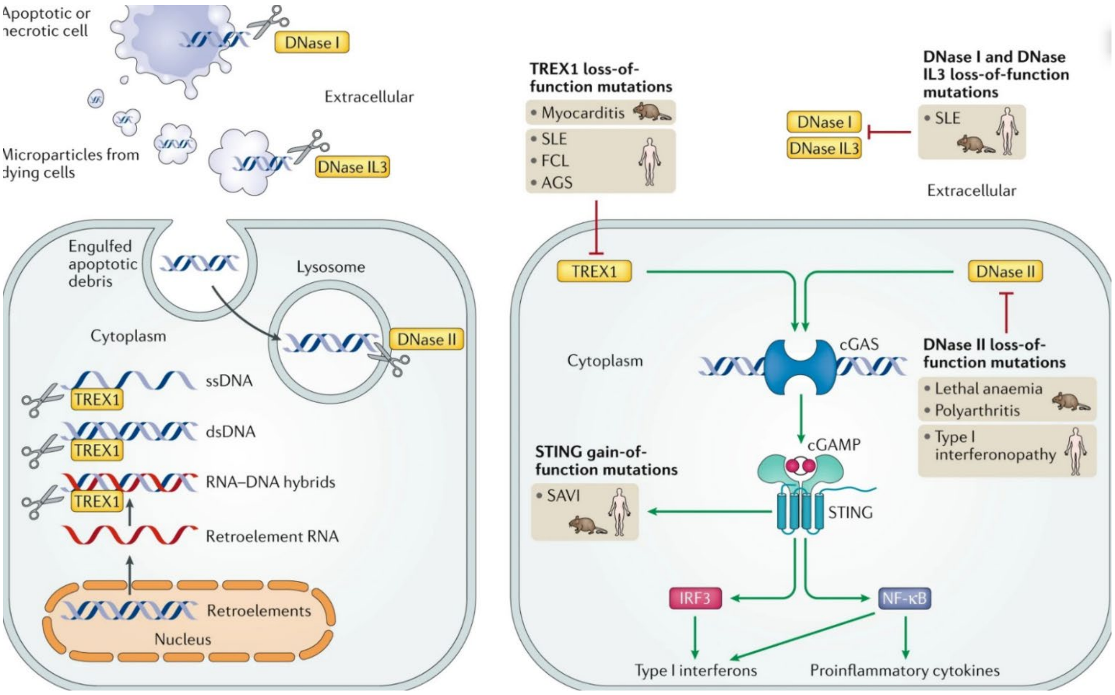

Example 3:

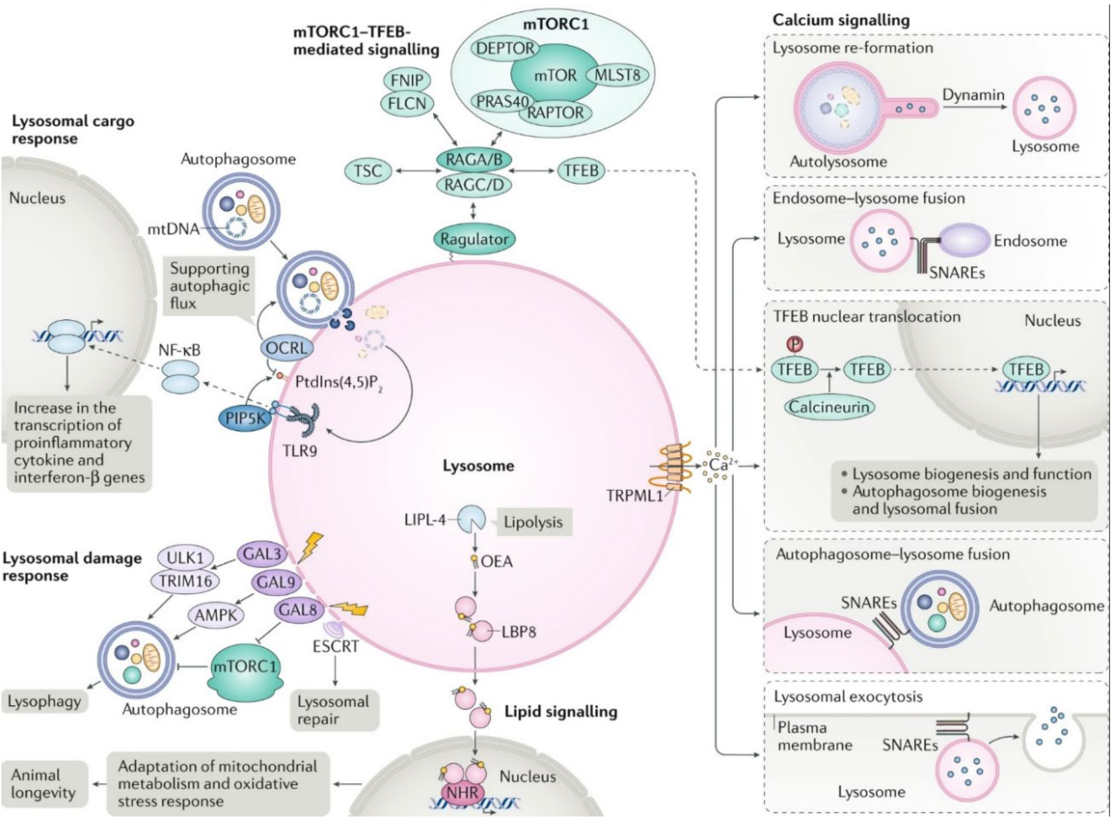

mTORC1–TFEB-mediated signalling
mTORC1
DEPTOR
mTOR
MLST8
PRAS40
RAPTOR
FNIP
FLCN
TSC
RAGA/B
RAGC/D
TFEB
Ragulator
Lysosomal cargo response
Nucleus
Autophagosome
mtDNA
Supporting autophagic flux
NF-κB
OCRL
PtdIns(4,5)P2
PIP5K
TLR9
Increase in the transcription of proinflammatory cytokine and interferon-β genes
Lysosome
LIPL-4
Lipolysis
OEA
LBP8
Lysosomal damage response
ULK1
TRIM16
GAL3
GAL9
AMPK
GAL8
ESCRT
mTORC1
Lysophagy
Autophagosome
Lysosomal repair
Lipid signalling
Nucleus
NHR
Animal longevity
Adaptation of mitochondrial metabolism and oxidative stress response
TRPML1
Ca2+
Calcium signalling
Lysosome re-formation
Dynamin
Autolysosome
Lysosome
Endosome–lysosome fusion
Lysosome
Endosome
SNAREs
TFEB nuclear translocation
Nucleus
P
TFEB
TFEB
TFEB
Calcineurin
• Lysosome biogenesis and function
• Autophagosome biogenesis and lysosomal fusion
Autophagosome–lysosome fusion
SNAREs
Autophagosome
Lysosome
Lysosomal exocytosis
Plasma membrane
SNAREs
Lysosome

Example 4:

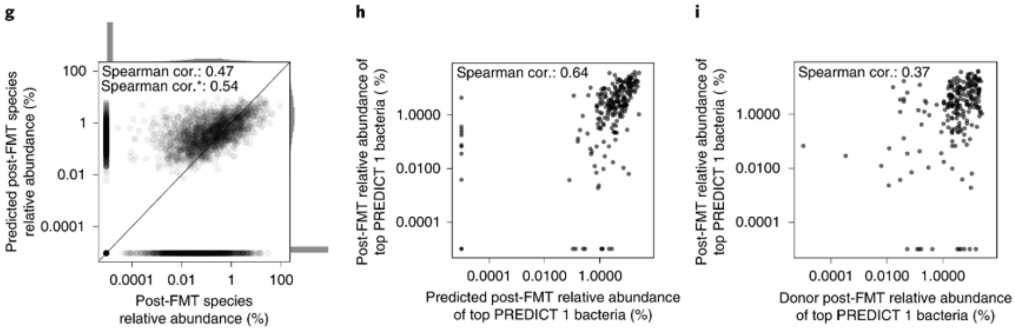
g
Spearman cor.: 0.47
Spearman cor.*: 0.54
Predicted post-FMT species relative abundance (%)
Post-FMT species relative abundance (%)
h
Spearman cor.: 0.64
Post-FMT relative abundance of top PREDICT 1 bacteria (%)
Predicted post-FMT relative abundance of top PREDICT 1 bacteria (%)
i
Spearman cor.: 0.37
Post-FMT relative abundance of top PREDICT 1 bacteria (%)
Donor post-FMT relative abundance of top PREDICT 1 bacteria (%)

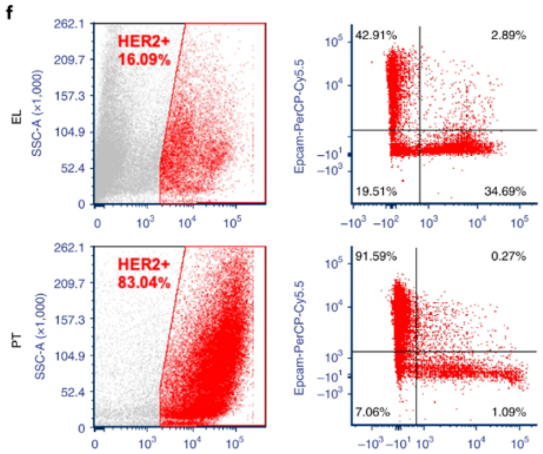
f
EL
PT
SSC-A (×1,000)
HER2+
16.09%
HER2+
83.04%
Epcam-PerCP-Cy5.5
42.91%
2.89%
19.51%
34.69%
91.59%
0.27%
7.06%
1.09%

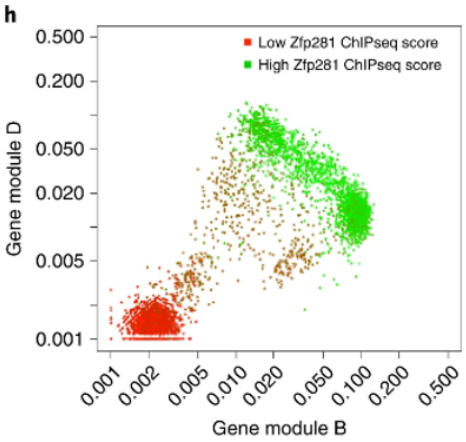
h
Low Zfp281 ChIPseq score
High Zfp281 ChIPseq score
Gene module D
Gene module B

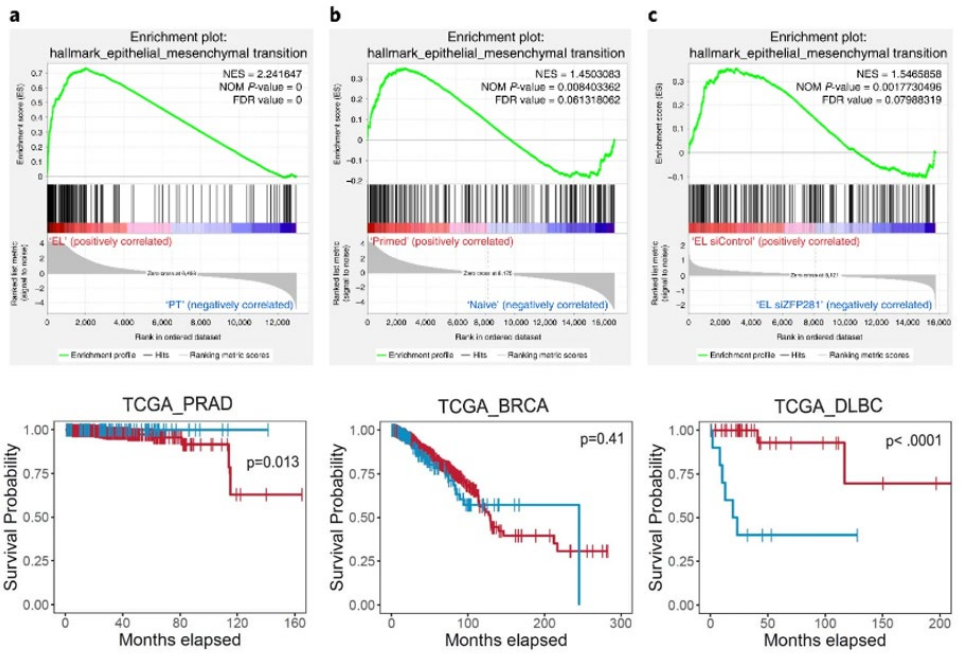
a
b
c
Enrichment plot:
hallmark_epithelial_mesenchymal transition
NES = 2.241647
NOM P-value = 0
FDR value = 0
NES = 1.4503083
NOM P-value = 0.008403362
FDR value = 0.061318062
NES = 1.5465858
NOM P-value = 0.0017730496
FDR value = 0.07988319
'EL' (positively correlated)
'PT' (negatively correlated)
'Primed' (positively correlated)
'Naive' (negatively correlated)
'EL siControl' (positively correlated)
'EL siZFP281' (negatively correlated)
Rank in ordered dataset
TCGA_PRAD
TCGA_BRCA
TCGA_DLBC
p=0.013
p=0.41
p< .0001
Survival Probability
Months elapsed

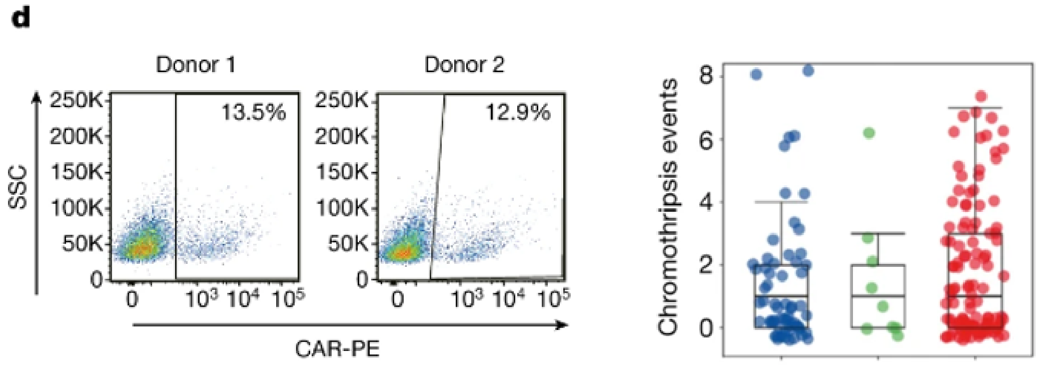
d
Donor 1
Donor 2
13.5%
12.9%
SSC
CAR-PE
Chromothripsis events

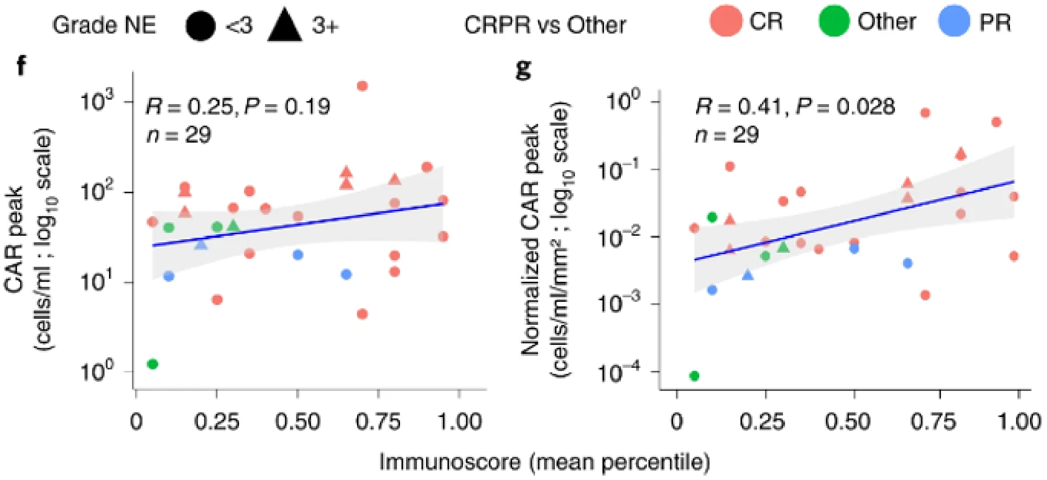
Grade NE
<3
3+
CRPR vs Other
CR
Other
PR
f
g
R = 0.25, P = 0.19
n = 29
R = 0.41, P = 0.028
n = 29
CAR peak
(cells/ml ; log10 scale)
Normalized CAR peak
(cells/ml/mm² ; log10 scale)
Immunoscore (mean percentile)

Example 5:

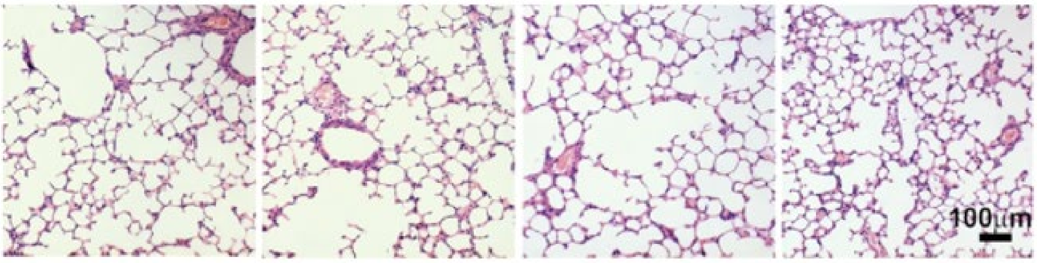

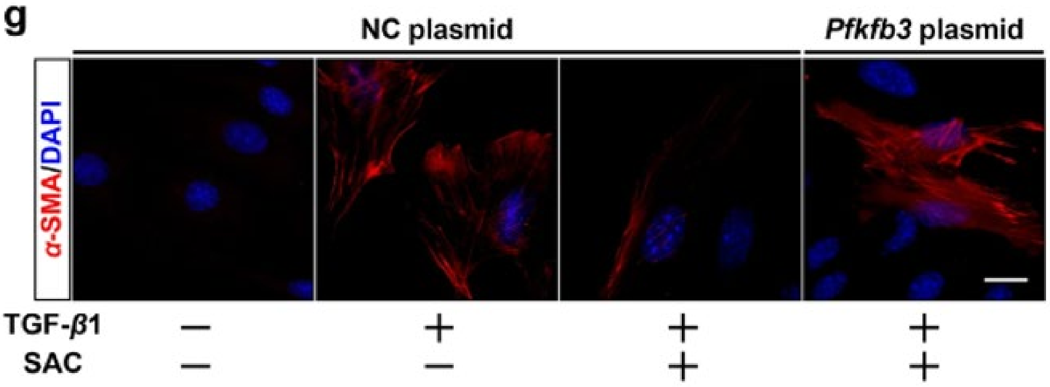

Others tips:

- When describing COVID-19-associated background and results, please avoid using the words such as “Wuhan virus/strain”, “Hubei”, “spread from China”, etc.
- **World map** is strongly **discouraged** from being used in figures.

For synthesized compounds

- For **first-reported new compounds**, please provide the color, melting point, yield, weight, NMR data (^{1}H, ^{13}C) and HRMS data. For the **reported compounds**, please provide the color, melting point, yield, weight, 1H-NMR (at least) and the referred literature. If the compound is used for activity test, the HPLC spectrum should be provided to prove that the purity is greater than 95%.

For crystal structures:

- **Homology Models:** Coordinates of homology models in PDB format should be submitted as Supporting Information for Publication.
- **PDB ID Codes:** Include the PDB ID codes with assigned compound Arabic number. Include the statement "Authors will release the atomic coordinates and experimental data upon article publication."

For Clinical trials:

- Title should be descriptive and include the type of study (e.g., a randomized controlled trial, phase 1/2/3/4, multicenter, double-blind, etc.
- The abstract must contain the study design, the participants, the interventions, the findings and the interpretation.
- The register number, the number of the patients are necessary in the abstract.
- A copy of the approved study protocol including the statistical analysis plan is recommended to be included with the initial submission (in English).
- Currently *STTT* does not publish case reports, case series and Meta-analyses.
- **Please change the flow chart or study design figure in color**. For example:

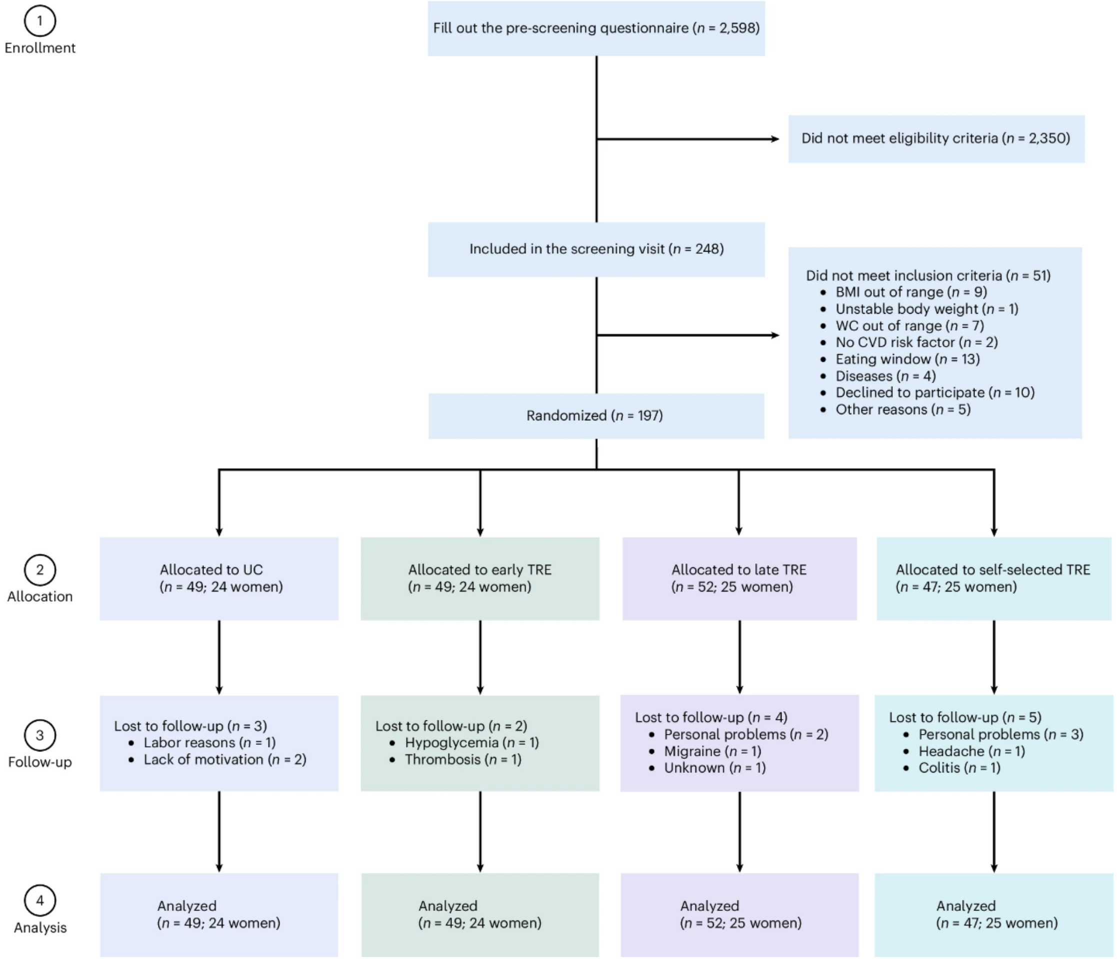

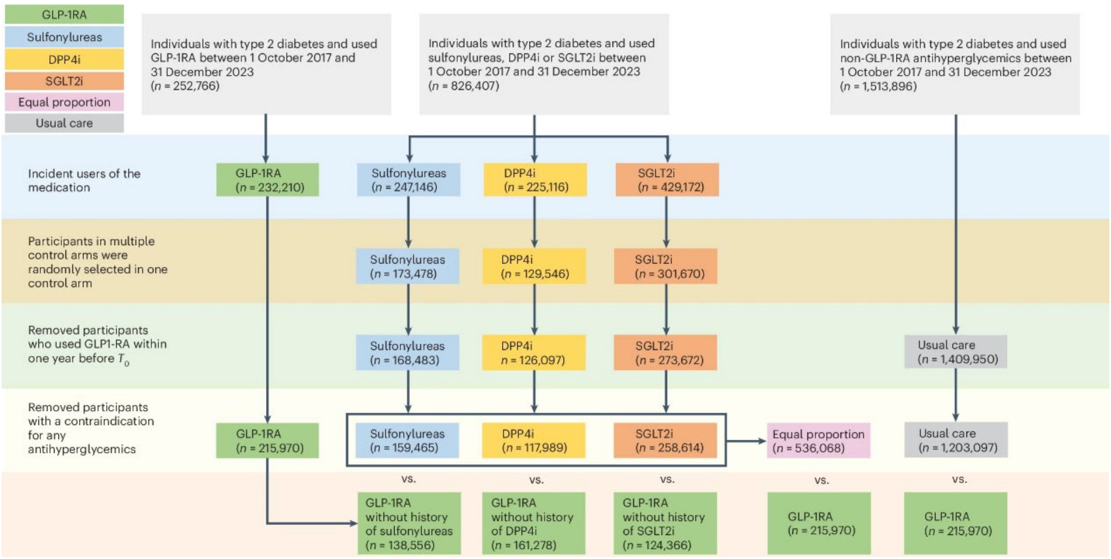
GLP-1RA
Sulfonylureas
DPP4i
SGLT2i
Equal proportion
Usual care
Individuals with type 2 diabetes and used GLP-1RA between 1 October 2017 and 31 December 2023 (n = 252,766)
Individuals with type 2 diabetes and used sulfonylureas, DPP4i or SGLT2i between 1 October 2017 and 31 December 2023 (n = 826,407)
Individuals with type 2 diabetes and used non-GLP-1RA antihyperglycemics between 1 October 2017 and 31 December 2023 (n = 1,513,896)
Incident users of the medication
GLP-1RA (n = 232,210)
Sulfonylureas (n = 247,146)
DPP4i (n = 225,116)
SGLT2i (n = 429,172)
Participants in multiple control arms were randomly selected in one control arm
Sulfonylureas (n = 173,478)
DPP4i (n = 129,546)
SGLT2i (n = 301,670)
Removed participants who used GLP1-RA within one year before T_0
Sulfonylureas (n = 168,483)
DPP4i n = 126,097)
SGLT2i (n = 273,672)
Usual care (n = 1,409,950)
Removed participants with a contraindication for any antihyperglycemics
GLP-1RA (n = 215,970)
Sulfonylureas (n = 159,465)
DPP4i (n = 117,989)
SGLT2i (n = 258,614)
Equal proportion (n = 536,068)
Usual care (n = 1,203,097)
vs.
vs.
vs.
vs.
vs.
GLP-1RA without history of sulfonylureas (n = 138,556)
GLP-1RA without history of DPP4i (n = 161,278)
GLP-1RA without history of SGLT2i (n = 124,366)
GLP-1RA (n = 215,970)
GLP-1RA (n = 215,970)
